# Supplementary material for: Cerebral organoids display dynamic clonal growth and tunable tissue replenishment
Source: Nat Cell Biol. 2024 May 7;26(5):710–8. doi: 10.1038/s41556-024-01412-z (PMC11098754; doi:10.1038/s41556-024-01412-z)
Supplement: Supplementary file 1 — Reporting Summary [file 41556_2024_1412_MOESM1_ESM.pdf]

nature portfolio

Corresponding author(s): Christopher Esk, Jürgen A. Knoblich

Last updated by author(s): Mar 14, 2024

Reporting Summary

Nature Portfolio wishes to improve the reproducibility of the work that we publish. This form provides structure for consistency and transparency in reporting. For further information on Nature Portfolio policies, see our Editorial Policies and the Editorial Policy Checklist.

Statistics

For all statistical analyses, confirm that the following items are present in the figure legend, table legend, main text, or Methods section.

n/a	Confirmed	
☐	☒	The exact sample size (n) for each experimental group/condition, given as a discrete number and unit of measurement
☐	☒	A statement on whether measurements were taken from distinct samples or whether the same sample was measured repeatedly
☐	☒	The statistical test(s) used AND whether they are one- or two-sided<br>*Only common tests should be described solely by name; describe more complex techniques in the Methods section.*
☒	☐	A description of all covariates tested
☒	☐	A description of any assumptions or corrections, such as tests of normality and adjustment for multiple comparisons
☒	☐	A full description of the statistical parameters including central tendency (e.g. means) or other basic estimates (e.g. regression coefficient) AND variation (e.g. standard deviation) or associated estimates of uncertainty (e.g. confidence intervals)
☐	☒	For null hypothesis testing, the test statistic (e.g. F, t, r) with confidence intervals, effect sizes, degrees of freedom and P value noted<br>*Give P values as exact values whenever suitable.*
☒	☐	For Bayesian analysis, information on the choice of priors and Markov chain Monte Carlo settings
☒	☐	For hierarchical and complex designs, identification of the appropriate level for tests and full reporting of outcomes
☒	☐	Estimates of effect sizes (e.g. Cohen's d, Pearson's r), indicating how they were calculated

Our web collection on statistics for biologists contains articles on many of the points above.

Software and code

Policy information about availability of computer code

Data collection	Data was collected using Zeiss (LSM 780, 800, 880, Z1), Olympus (IX83) and Yokogawa (CSU-W1) microscopes and a slidescanner (3DHistech, Panoramic250) with the companies standard softwares. Flow cytometry was performed using BD Fortessa instruments running Diva (V9.0.1) software. Illumina sequencers (HiSeq and NovaSeq) were used for NGS experiments. Single cell RNA-seq experiments were performed using the 10x Genomics Chromium controller.
Data analysis	Data analysis was performed using Microsoft Excel (V. 16.83), Graphpad Prism (V.9 and newer), FlowJo, Seurat in R (V. 4.3.0.1.), Python (V. 3.9.), ImageJ (V. 1.5.3) and CellRanger (V. 7.01) . Code for stochastic modeling is available here: https://github.com/Cibiv/pyrganoid

For manuscripts utilizing custom algorithms or software that are central to the research but not yet described in published literature, software must be made available to editors and reviewers. We strongly encourage code deposition in a community repository (e.g. GitHub). See the Nature Portfolio guidelines for submitting code & software for further information.

Data

Policy information about availability of data

All manuscripts must include a data availability statement. This statement should provide the following information, where applicable:

- Accession codes, unique identifiers, or web links for publicly available datasets
- A description of any restrictions on data availability
- For clinical datasets or third party data, please ensure that the statement adheres to our policy

NGS data used in this work is available under GEO accession no.: NCBI GSE214105, GSE151384. The human reference genome used was GRCh38 2020.

Human research participants

Policy information about studies involving human research participants and Sex and Gender in Research.

Reporting on sex and gender	Not applicable.
Population characteristics	Not applicable.
Recruitment	Not applicable.
Ethics oversight	Not applicable.

Note that full information on the approval of the study protocol must also be provided in the manuscript.

Field-specific reporting

Please select the one below that is the best fit for your research. If you are not sure, read the appropriate sections before making your selection.

☒ Life sciences ☐ Behavioural & social sciences ☐ Ecological, evolutionary & environmental sciences

For a reference copy of the document with all sections, see nature.com/documents/nr-reporting-summary-flat.pdf

Life sciences study design

All studies must disclose on these points even when the disclosure is negative.

Sample size	Samples sizes for microscopy, organoid size measurements and flow cytometry analysis and were chosen based on previous experience and literature. Lineage tracing studies were performed at least in triplicates for all timepoints in all conditions. This approach is in line with standards in the field (Lancaster et al, Nature Biotechnology, 2017; Bagley et al, Nature Methods, 2017; Esk et al, Science, 2020; Eichmüller et al, Science, 2020).
Data exclusions	20 lineage tracing samples (out of 476) were discarded due to low / no PCR amplicon generation. In those cases duplicate (instead of planned triplicate) measurements were used.
Replication	At least two independent biological experiments were performed including multiple replicates for most experiments. Details are provided in figure legends. Exceptions include some whole organoid lineage tracing experiments, which were conducted only once with three individual organoids per timepoint and conditions and four mice per timepoint. Barcoded scRNA-seq experiments were performed from one batch with one organoid per 10X library in organoid duplicates per two timepoints.
Randomization	Individual organoids analyzed were randomly chosen from a running batch for each type of experiment. Randomization of subjects was not relevant in this study.
Blinding	The experimenters were not blinded. Analyses were performed predominantly using unbiased computers and results reported are mostly descriptive.

Reporting for specific materials, systems and methods

We require information from authors about some types of materials, experimental systems and methods used in many studies. Here, indicate whether each material, system or method listed is relevant to your study. If you are not sure if a list item applies to your research, read the appropriate section before selecting a response.

Materials & experimental systems

n/a	Involved in the study
☐	☒ Antibodies
☐	☒ Eukaryotic cell lines
☒	☐ Palaeontology and archaeology
☐	☒ Animals and other organisms
☒	☐ Clinical data
☒	☐ Dual use research of concern

Methods

n/a	Involved in the study
☒	☐ ChIP-seq
☐	☒ Flow cytometry
☒	☐ MRI-based neuroimaging

Antibodies

Antibodies used

The following primary antibodies were used:

anti-SOX2 (Abcam, ab97959, 1:500),
anti-NEUN (Millipore, MAB377, 1:500),
anti-DCX (Santa Cruz, sc8066, 1:1000),
anti-MAP2 (Abcam, ab5392, 1:1000),
anti-Nestin (BD, 611658, 1:1000),
anti bIII-tubulin (Abcam, ab18207, 1:1000),
anti-BrdU (ThermoFisher, B35128, 1:500),
anti phospho-vimentin(Ser82)(MBL, D095-3S, 1:250),
anti-TTR (AbD Serotec, ahp1837, 1:500),
anti-aPKC (SantaCruz, sc-17781, 1:100),
anti nestin (Abcam, ab105389, 1:1000),
anti-b-catenin (Sigma, C2206, 1:250),
anti-n-cadherin (BD, 610920, 1:250),
anti-TBR1 (Abcam, ab31940, 1:500),
anti-TBR2 (Abcam, ab23345, 1:250),
anti-ASPM (Bethyl Laboratories, IHC-00058, 1:100),
anti-EOMES (R&D Sytems AF6166, 1:200),
chicken anti-GFP (Abcam Ab13970, 1:500),
mouse anti-Sox2 (Abcam Ab79351, 1:500),
rabbit anti-NeuroD2 (Abcam Ab104430, 1:500),
anti-LaminB1 (1:2000, GeneTex, cat #103292),
anti-PAX6 (1:1000, Biolegend, cat #901301),
anti-TP53 (1:1000, AbCam, #ab32389),
anti-NCAM (BD Biosciences, cat.: 564058, 1:500),
anti-CXCR4 (BD Biosciences, cat.: 560936, 1:250),
anti-TRA-1-60 (BD Biosciences, cat.: 563188, 1:250).

Secondary antibodies raised in donkey or goat were purchased from Invitrogen. If possible, all secondary antibodies were of highly cross-adsorbed quality.

Donkey anti-Mouse IgG (H+L), Alexa Fluor™ 488 (Thermo Fisher Scientific, cat. A-21202, 1:1000),
Donkey anti-Rabbit IgG (H+L), Alexa Fluor™ 488 (Thermo Fisher Scientific, cat. A-21206, 1:1000),
Donkey anti-Goat IgG (H+L), Alexa Fluor™ 488, (Thermo Fisher Scientific, cat. A-11055, 1:1000),
Donkey anti-Sheep IgG (H+L), Alexa Fluor™ 488 (Thermo Fisher Scientific, cat. A-11015, 1:1000),
Goat anti-Rabbit IgG (H+L), Alexa Fluor™ 488 (Thermo Fisher Scientific, cat. A-11008, 1:1000),
Goat anti-Mouse IgG (H+L), Alexa Fluor™ 488 (Thermo Fisher Scientific, cat. A-11001, 1:1000),
Goat anti-Mouse IgG1, Alexa Fluor™ 488 (Thermo Fisher Scientific, cat. A-21121, 1:1000),
Goat anti-Rabbit IgG (H+L), Alexa Fluor™ 568 (Thermo Fisher Scientific, cat. A-11011, 1:1000),
Goat anti-Mouse IgG (H+L), Alexa Fluor™ 568 (Thermo Fisher Scientific, cat. A-11004, 1:1000),
Donkey anti-Goat IgG (H+L), Alexa Fluor™ 568 (Thermo Fisher Scientific, cat. A-11057, 1:1000),
Donkey anti-Mouse IgG (H+L), Alexa Fluor™ 568 (Thermo Fisher Scientific, cat. A-11037, 1:1000),
Goat anti-Mouse IgG2b, Alexa Fluor™ 568 (Thermo Fisher Scientific, cat. A-21144, 1:1000),
Donkey anti-Chicken IgY (H+L), Alexa Fluor™ 568 (Thermo Fisher Scientific, cat. A-78950, 1:1000),
Donkey anti-Rabbit IgG (H+L), Alexa Fluor™ 647 (Thermo Fisher Scientific, cat. 31573, 1:1000),
Donkey anti-Goat IgG (H+L), Alexa Fluor™ 647 (Thermo Fisher Scientific, cat. 21447, 1:1000),
Goat anti-Rabbit IgG (H+L), Alexa Fluor™ 647 (Thermo Fisher Scientific, cat. 21245, 1:1000),
Goat anti-Rabbit IgG (H+L), Alexa Fluor™ 647 (Thermo Fisher Scientific, cat. 21244, 1:1000).

Validation

anti-SOX2 (Abcam, ab97959) has been validated by the company and referenced in 656 publications.
anti-NEUN (Millipore, MAB377) has been validated by the company and referenced in more than 100 publications.
anti-DCX (Santa Cruz, sc8066) has been validated by the company and referenced in 226 publications.
anti-MAP2 (Abcam, ab5392) has been validated by the company and referenced in 511 publications.
anti-Nestin (BD, 611658) has been validated by the company and referenced in 5 publications.
anti bIII-tubulin (Abcam, ab18207) has been validated by the company and referenced in 379 publications.
anti-BrdU (ThermoFisher, B35128) has been validated by the company and referenced in 60 publications.
anti phospho-vimentin(Ser82)(MBL, D095-3S) has been validated by the company and referenced in 10 publications.

anti-TTR (AbD Serotec, ahp1837) has been validated by the company and referenced in 9 publications.
anti-aPKC (SantaCruz, sc-17781) has been validated by the company and referenced in 138 publications.
anti nestin (Abcam, ab105389) has been validated by the company and referenced in 40 publications.
anti-b-catenin (Sigma, C2206) has been validated by the company and referenced in 370 publications.
anti-n-cadherin (BD, 610920) has been validated by the company and referenced in 5 publications.
anti-TBR1 (Abcam, ab31940) has been validated by the company and referenced in 322 publications.
anti-TBR2 (Abcam, ab23345) has been validated by the company and referenced in 392 publications.
anti-ASPM (Bethyl Laboratories, IHC-00058) has been validated by the company and referenced in 7 publications.
anti-EOMES (R&D Sytems AF6166) has been validated by the company and referenced in 11publications.
chicken anti-GFP (Abcam Ab13970) has been validated by the company and referenced in 2531 publications.
mouse anti-Sox2 (Abcam Ab79351) has been validated by the company and referenced in 49 publications.
rabbit anti-NeuroD2 (Abcam Ab104430) has been validated by the company and referenced in 14 publications.
anti-LaminB1 (GeneTex, cat #103292) has been validated by the company and referenced in 39 publications.
anti-PAX6 (Biolegend, cat #901301) has been validated by the company and referenced in 308 publications.
anti-TP53 (AbCam, #ab32389) has been validated by the company and referenced in 53 publications.
anti-NCAM (BD Biosciences, cat.: 564058) has been validated by the company and referenced in 14 publications.
anti-CXCR4 (BD Biosciences, cat.: 560936) has been validated by the company and referenced in 5 publications.
anti-TRA-1-60 (BD Biosciences, cat.: 563188) has been validated by the company and referenced in 6 publications.

Eukaryotic cell lines

Policy information about cell lines and Sex and Gender in Research

Cell line source(s)
The HEK293 line was purchased from ATCC. The hESC line WA09 (H9) was obtained from WiCell. All other lines used in the study were generated in the lab.

Authentication
The hESC line WA09 (H9) obtained from WiCell was not independently authenticated. The line HEK293 purchased from ATCC was not independently authenticated.

Mycoplasma contamination
Cell cultures were routinely bi-monthly tested and confirmed negative for mycoplasma.

Commonly misidentified lines
(See ICLAC register)
No commonly misidentified lines have been used in this study.

Animals and other research organisms

Policy information about studies involving animals; ARRIVE guidelines recommended for reporting animal research, and Sex and Gender in Research

Laboratory animals
10-12 week old timed pregnant C57BL/6J mice (The Jackson Laboratory) were used in this study. Mice were kept at 22C +/-1C, 55% +/- 5% humidity in a 14h light / 10h dark cycle under the care of the IMBA's animal care facility.

Wild animals
No wild animals were used in this study.

Reporting on sex
Mouse embryos analyzed at day E17.5 were not tested for sex.

Field-collected samples
No field collected samples were used in this study.

Ethics oversight
All experimental procedures were approved by the Austrian Federal Ministry of Education, Science and Research under the animal experiment license BMWF.66.015/0023.WF/3b/2017. 3R principles were followed and monitored by the IMBA Ethics and Biosafety department.

Note that full information on the approval of the study protocol must also be provided in the manuscript.

Flow Cytometry

Plots

Confirm that:

☒ The axis labels state the marker and fluorochrome used (e.g. CD4-FITC).

☒ The axis scales are clearly visible. Include numbers along axes only for bottom left plot of group (a 'group' is an analysis of identical markers).

☐ All plots are contour plots with outliers or pseudocolor plots.

☒ A numerical value for number of cells or percentage (with statistics) is provided.

Methodology

Sample preparation
For flow cytometry analysis organoids were dissociated by incubating on a Thermo Shaker at 37 °C in a 10:1 mixture of Accutase (Sigma Aldrich, cat # A6954) and 10x Trypsin (Gibco, cat # 15400) to generate a single cell suspension, followed by filtering through a 35µm cell strainer.

Instrument	BD Fortessa
Software	FACS Diva
Cell population abundance	A minimum of 20000 single cells was analyzed for each condition at each timepoint.
Gating strategy	Single cells were gated using forward and side scatters. Amplifier settiings were chosen to clearly display negative and positive populations. Gating strategies are provided in the Extended Data.

☒ Tick this box to confirm that a figure exemplifying the gating strategy is provided in the Supplementary Information.
