# Supplementary figures and images for: Cerebral organoids display dynamic clonal growth and tunable tissue replenishment (part 1 of 2)
Source: Nat Cell Biol. 2024 May 7;26(5):710–8. doi: 10.1038/s41556-024-01412-z (PMC11098754; doi:10.1038/s41556-024-01412-z)

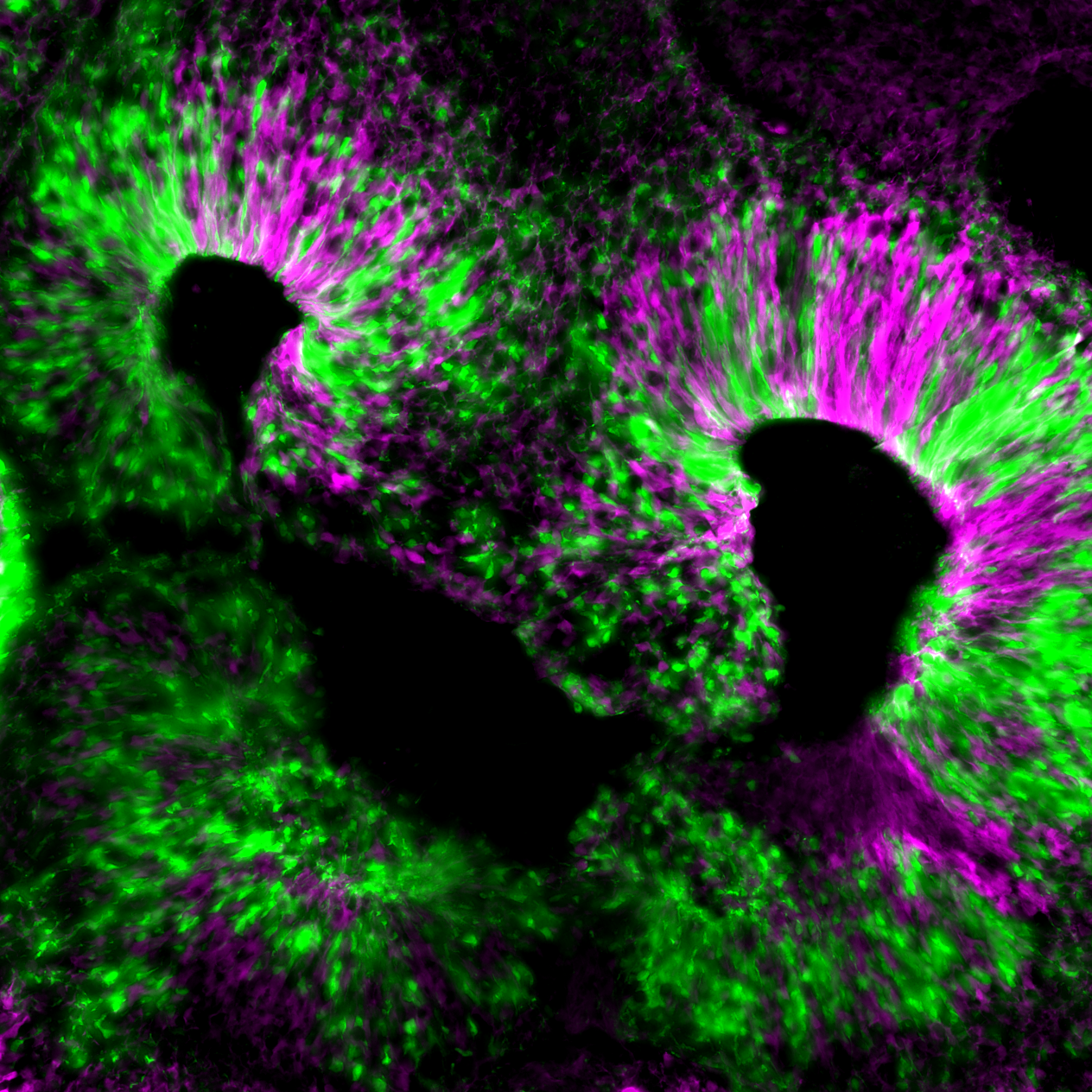

Supplement: Supplementary file 8 — Source data. [file 41556_2024_1412_MOESM8_ESM.zip › Lindenhoferetal-Fig-5-sourcedata-NCB/Lindenhoferetal-Fig-5-images-NCB/Lindenhoferetal-Fig-5-d-RFPWT-GFPWT-GFP-RFP.tif]

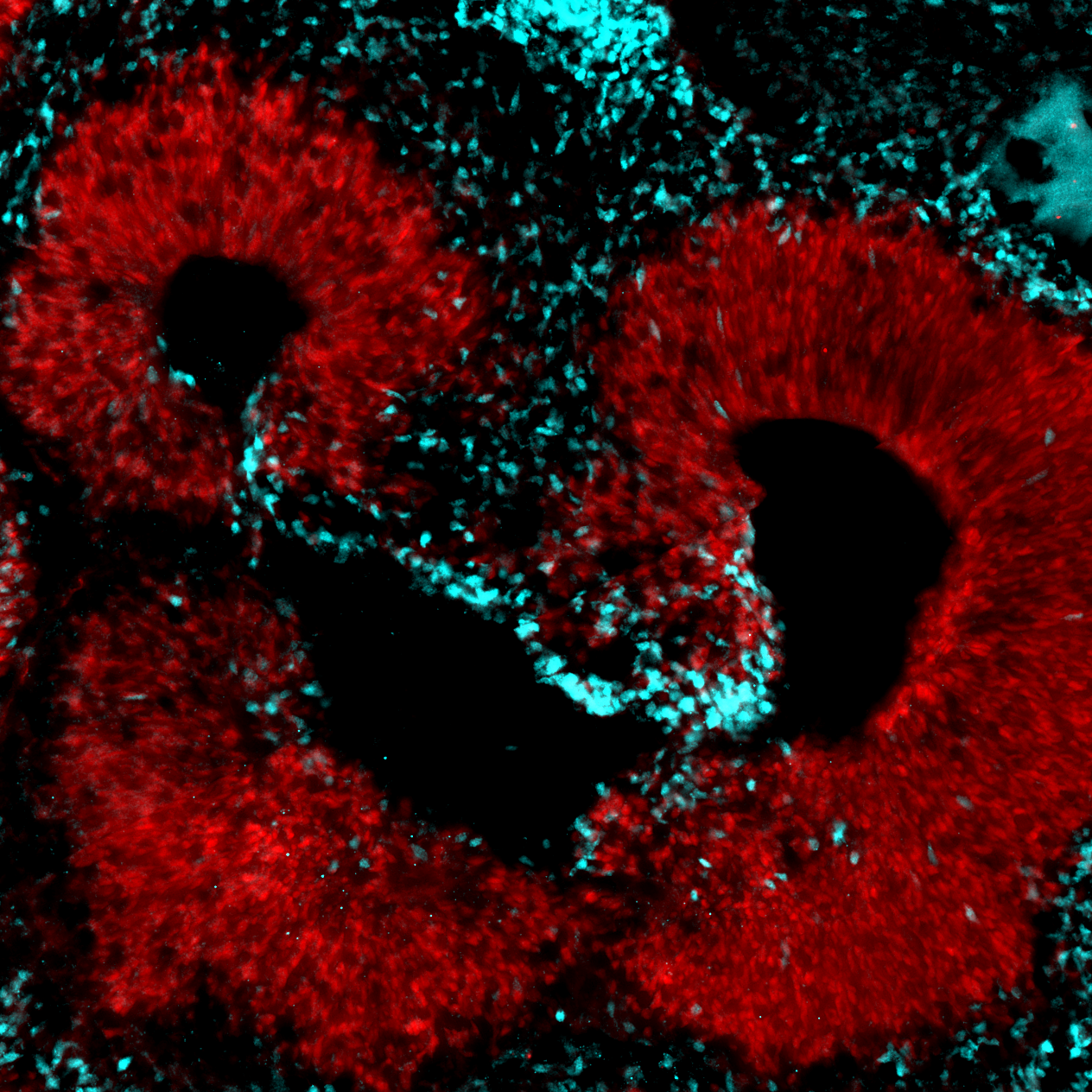

Supplement: Supplementary file 8 — Source data. [file 41556_2024_1412_MOESM8_ESM.zip › Lindenhoferetal-Fig-5-sourcedata-NCB/Lindenhoferetal-Fig-5-images-NCB/Lindenhoferetal-Fig-5-d-RFPWT-GFPWT-Sox2-NeuN.tif]

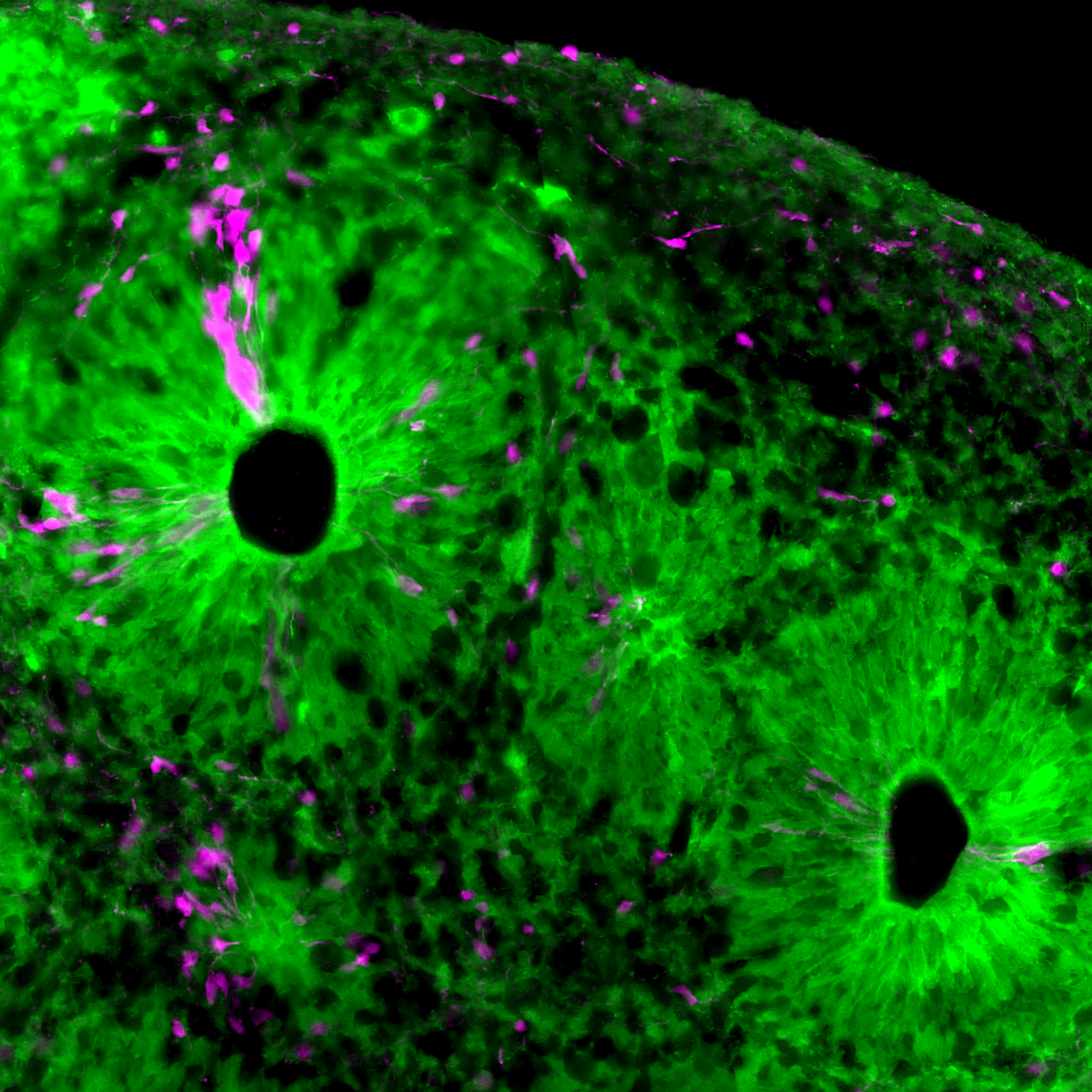

Supplement: Supplementary file 8 — Source data. [file 41556_2024_1412_MOESM8_ESM.zip › Lindenhoferetal-Fig-5-sourcedata-NCB/Lindenhoferetal-Fig-5-images-NCB/Lindenhoferetal-Fig-5-d-RFPWT-GFPKOTP53-GFP-RFP.tif]

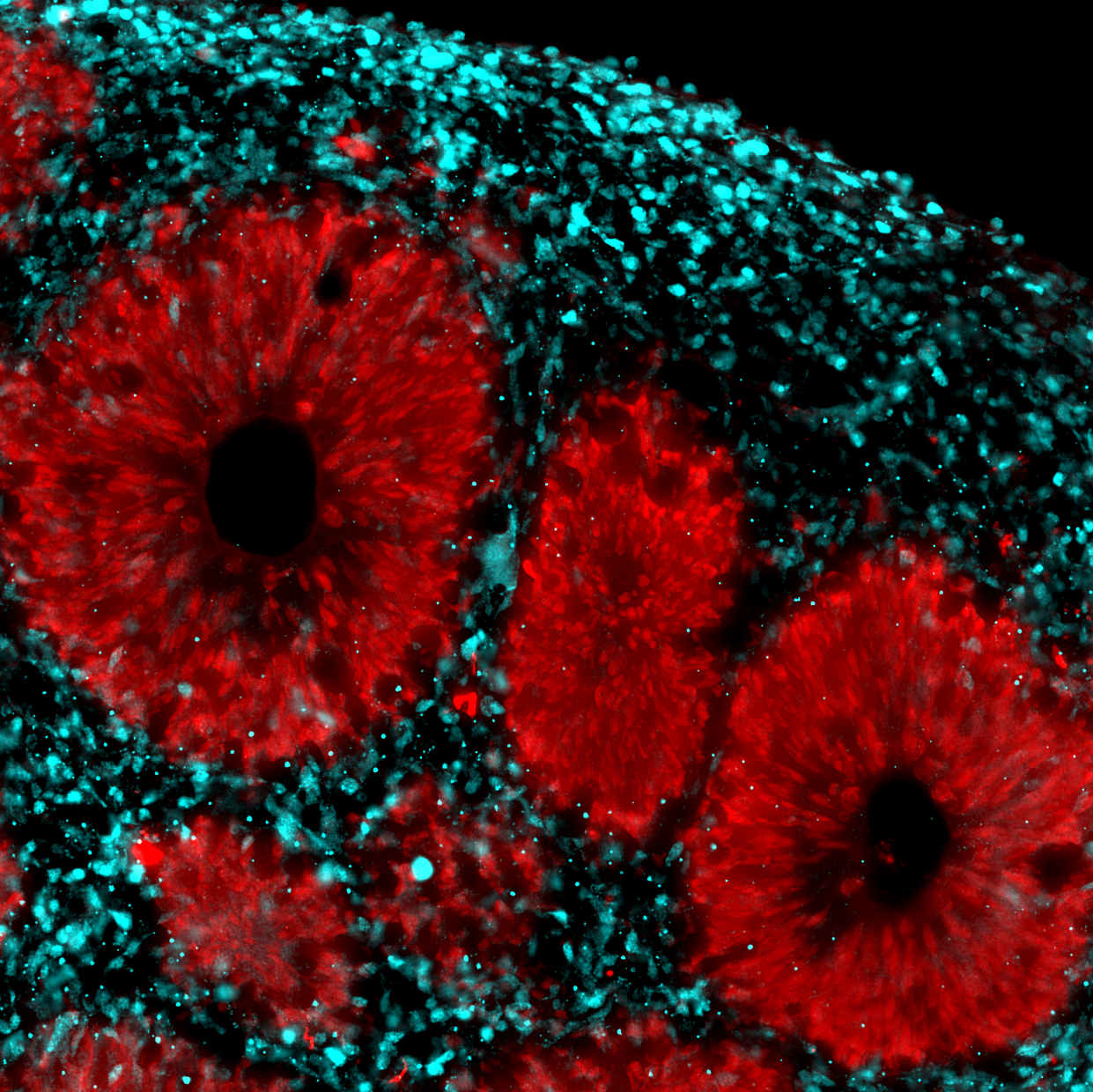

Supplement: Supplementary file 8 — Source data. [file 41556_2024_1412_MOESM8_ESM.zip › Lindenhoferetal-Fig-5-sourcedata-NCB/Lindenhoferetal-Fig-5-images-NCB/Lindenhoferetal-Fig-5-d-RFPWT-GFPKOTP53-Sox2-NeuN.tif]

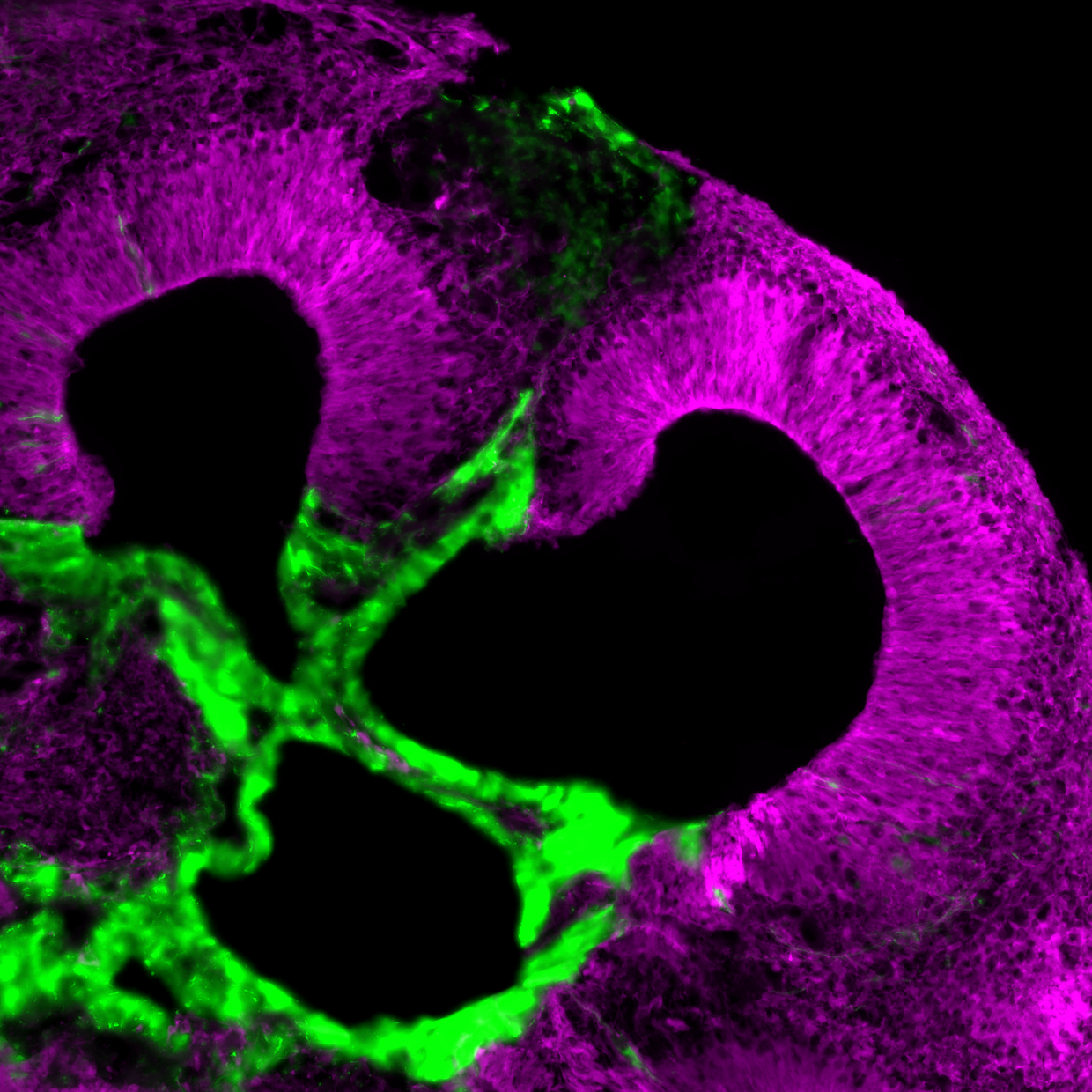

Supplement: Supplementary file 8 — Source data. [file 41556_2024_1412_MOESM8_ESM.zip › Lindenhoferetal-Fig-5-sourcedata-NCB/Lindenhoferetal-Fig-5-images-NCB/Lindenhoferetal-Fig-5-d-RFTWT-GFPKOPAX6-GFP-RFP.tif]

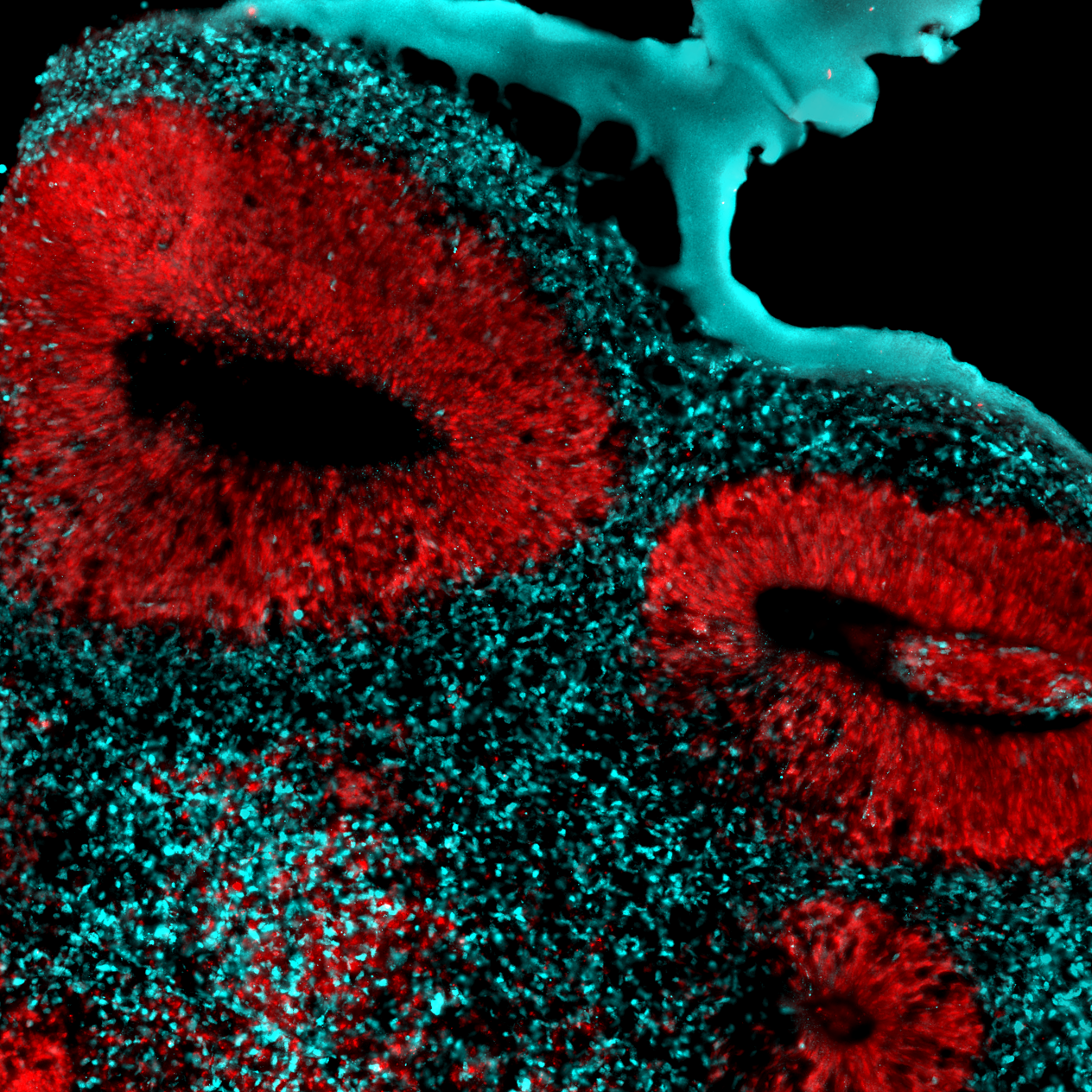

Supplement: Supplementary file 8 — Source data. [file 41556_2024_1412_MOESM8_ESM.zip › Lindenhoferetal-Fig-5-sourcedata-NCB/Lindenhoferetal-Fig-5-images-NCB/Lindenhoferetal-Fig-5-d-RFTWT-GFPKOASPM-Sox2-NeuN.tif]

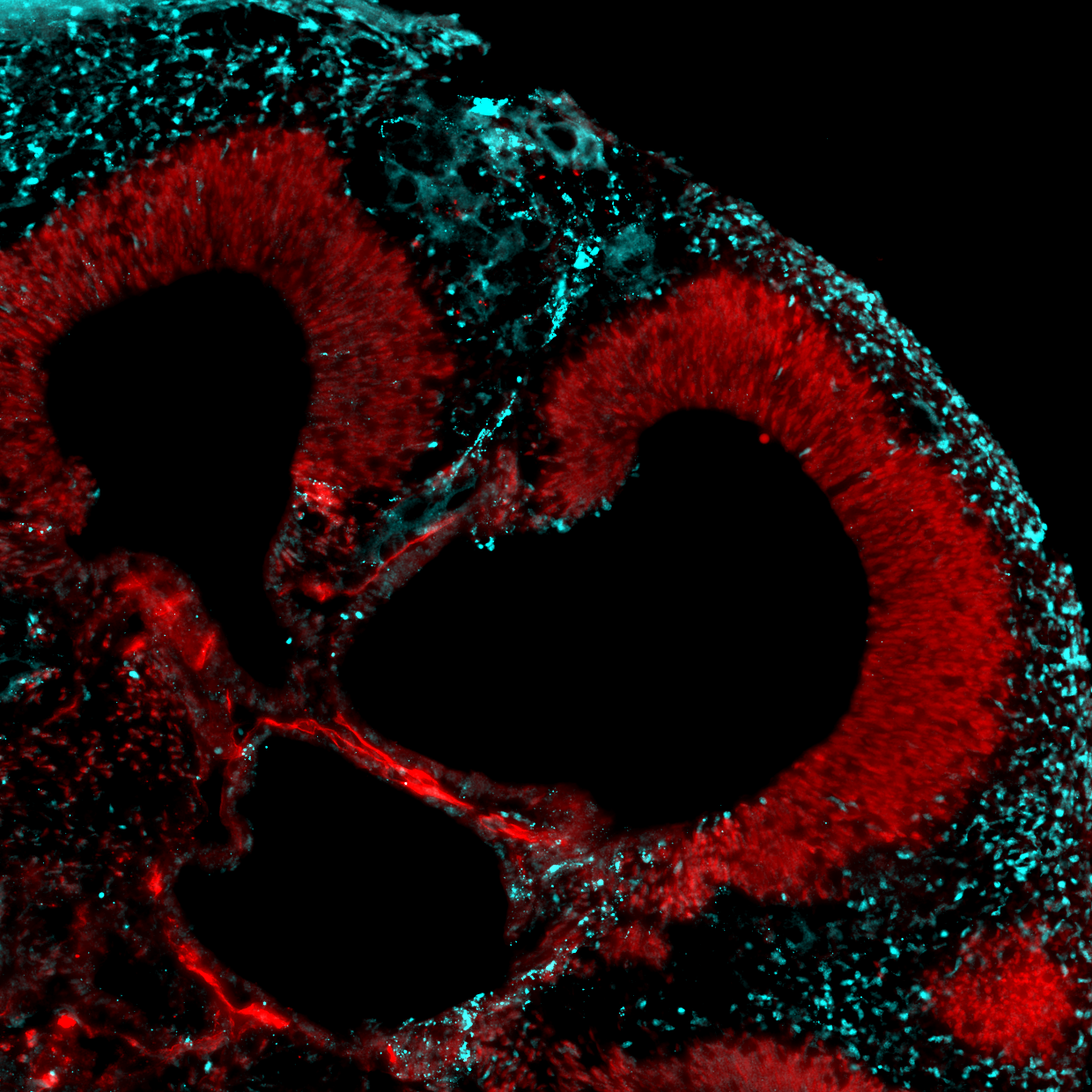

Supplement: Supplementary file 8 — Source data. [file 41556_2024_1412_MOESM8_ESM.zip › Lindenhoferetal-Fig-5-sourcedata-NCB/Lindenhoferetal-Fig-5-images-NCB/Lindenhoferetal-Fig-5-d-RFTWT-GFPKOPAX6-Sox2-NeuN.tif]

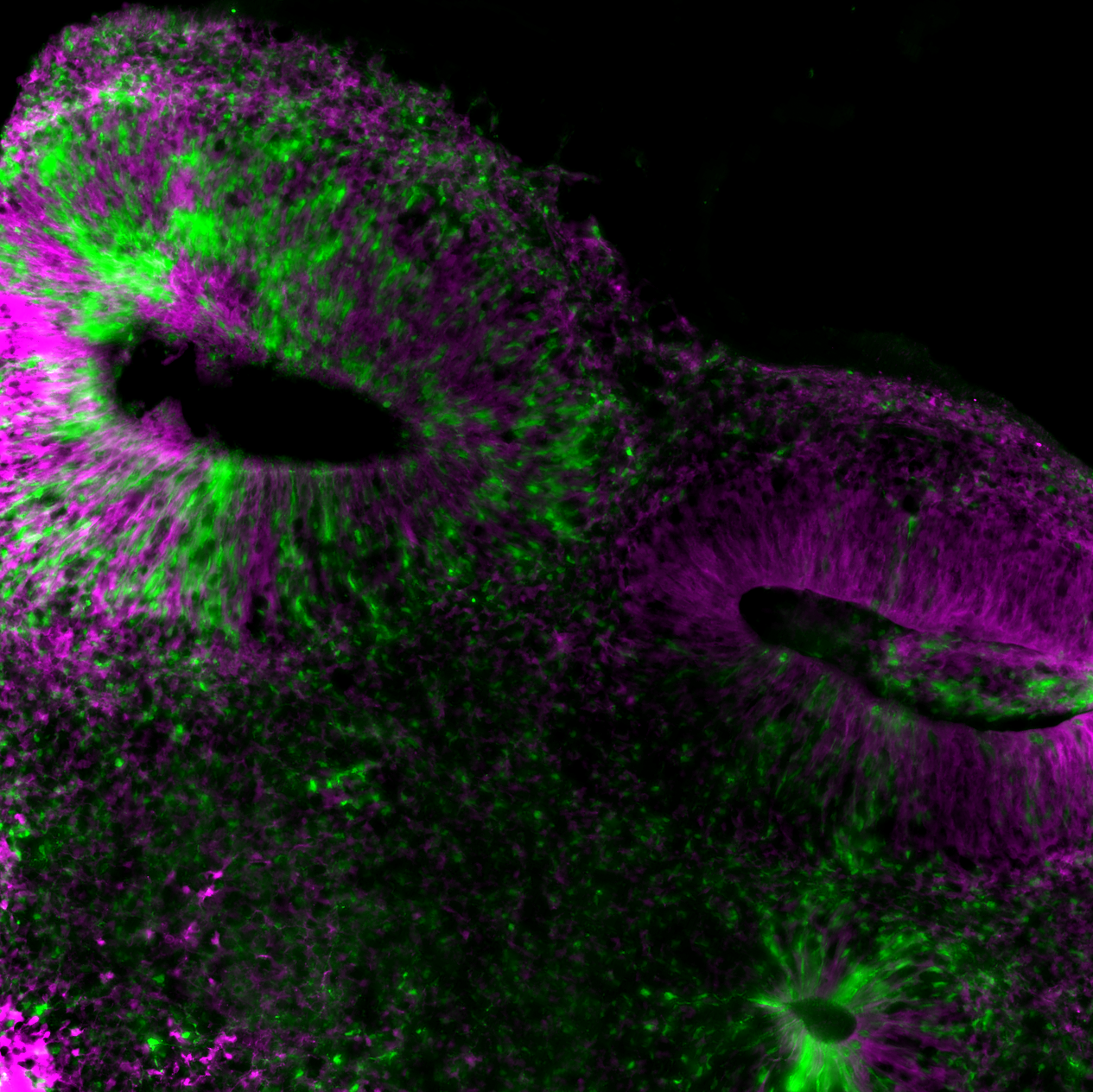

Supplement: Supplementary file 8 — Source data. [file 41556_2024_1412_MOESM8_ESM.zip › Lindenhoferetal-Fig-5-sourcedata-NCB/Lindenhoferetal-Fig-5-images-NCB/Lindenhoferetal-Fig-5-d-RFTWT-GFPKOASPM-GFP-RFP.tif]

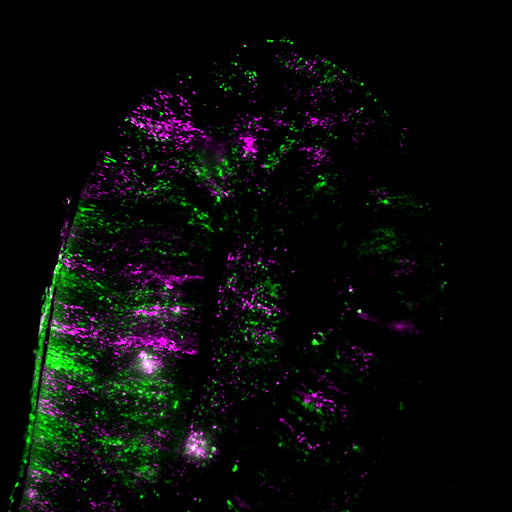

Supplement: Supplementary file 10 — Source data. [file 41556_2024_1412_MOESM10_ESM.zip › Lindenhoferetal-Fig-ED2-sourcedata-NCB/Lindenhoferetal-Fig-ED2f-images-NCB/E10toE17-RSFGGTT-z500.tif]

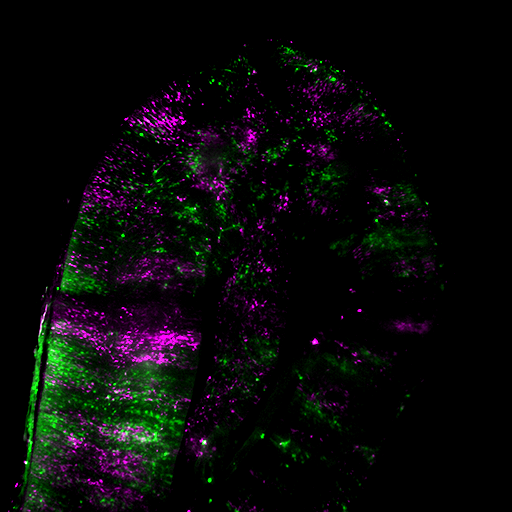

Supplement: Supplementary file 10 — Source data. [file 41556_2024_1412_MOESM10_ESM.zip › Lindenhoferetal-Fig-ED2-sourcedata-NCB/Lindenhoferetal-Fig-ED2f-images-NCB/E10toE17-RSFGGTT-z460.tif]

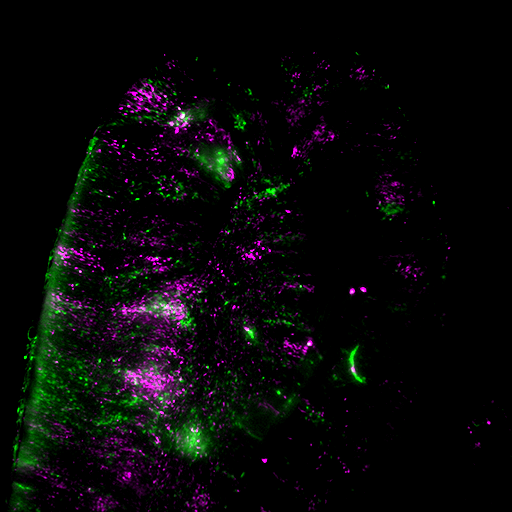

Supplement: Supplementary file 10 — Source data. [file 41556_2024_1412_MOESM10_ESM.zip › Lindenhoferetal-Fig-ED2-sourcedata-NCB/Lindenhoferetal-Fig-ED2f-images-NCB/E10toE17-RSFGGTT-z700.tif]

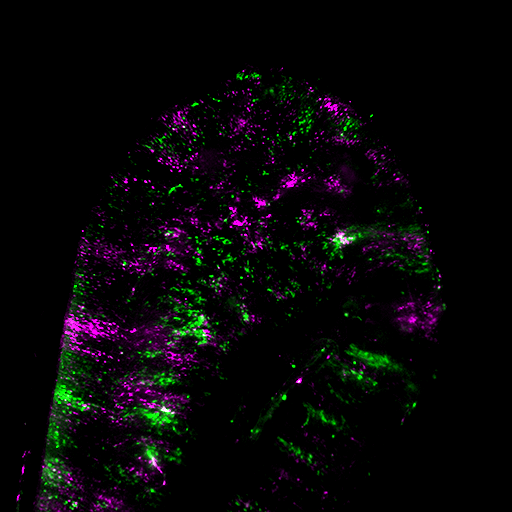

Supplement: Supplementary file 10 — Source data. [file 41556_2024_1412_MOESM10_ESM.zip › Lindenhoferetal-Fig-ED2-sourcedata-NCB/Lindenhoferetal-Fig-ED2f-images-NCB/E10toE17-RSFGGTT-z300.tif]

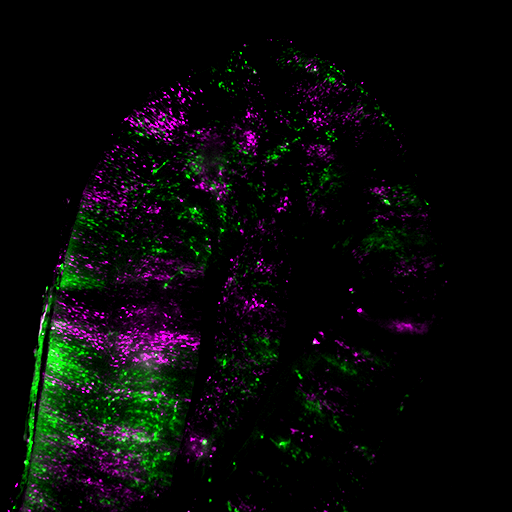

Supplement: Supplementary file 10 — Source data. [file 41556_2024_1412_MOESM10_ESM.zip › Lindenhoferetal-Fig-ED2-sourcedata-NCB/Lindenhoferetal-Fig-ED2f-images-NCB/E10toE17-RSFGGTT-z470.tif]

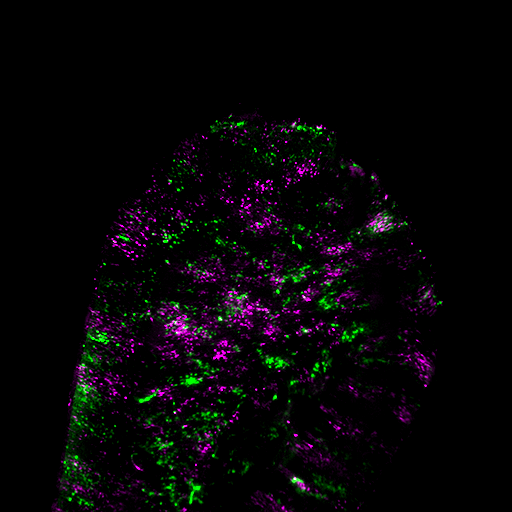

Supplement: Supplementary file 10 — Source data. [file 41556_2024_1412_MOESM10_ESM.zip › Lindenhoferetal-Fig-ED2-sourcedata-NCB/Lindenhoferetal-Fig-ED2f-images-NCB/E10toE17-RSFGGTT-z100.tif]

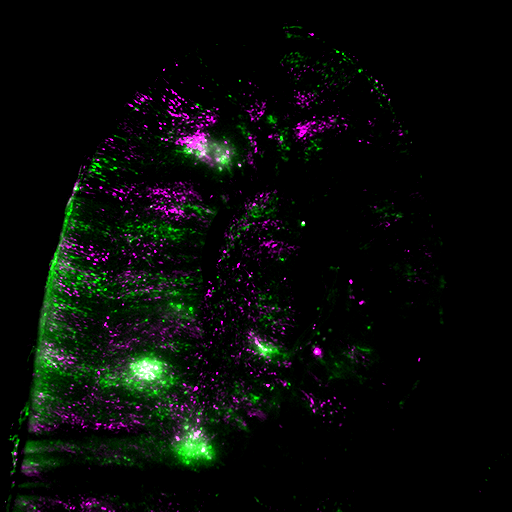

Supplement: Supplementary file 10 — Source data. [file 41556_2024_1412_MOESM10_ESM.zip › Lindenhoferetal-Fig-ED2-sourcedata-NCB/Lindenhoferetal-Fig-ED2f-images-NCB/E10toE17-RSFGGTT-z600.tif]

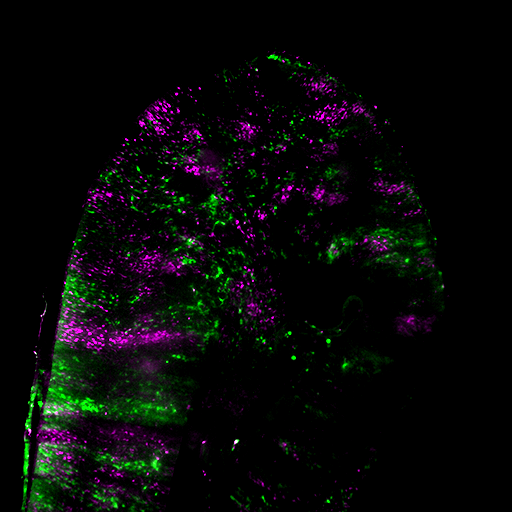

Supplement: Supplementary file 10 — Source data. [file 41556_2024_1412_MOESM10_ESM.zip › Lindenhoferetal-Fig-ED2-sourcedata-NCB/Lindenhoferetal-Fig-ED2f-images-NCB/E10toE17-RSFGGTT-z400.tif]

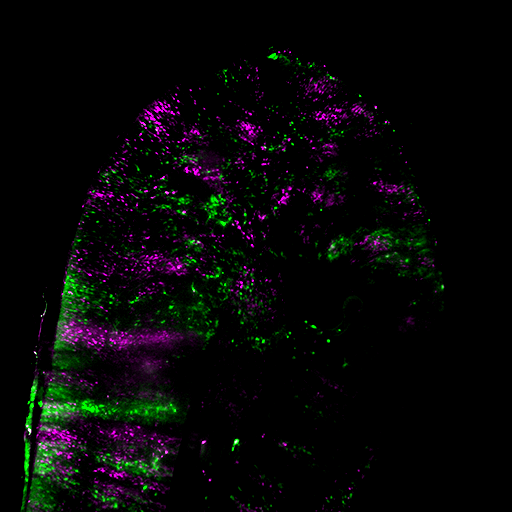

Supplement: Supplementary file 10 — Source data. [file 41556_2024_1412_MOESM10_ESM.zip › Lindenhoferetal-Fig-ED2-sourcedata-NCB/Lindenhoferetal-Fig-ED2f-images-NCB/E10toE17-RSFGGTT-z410.tif]

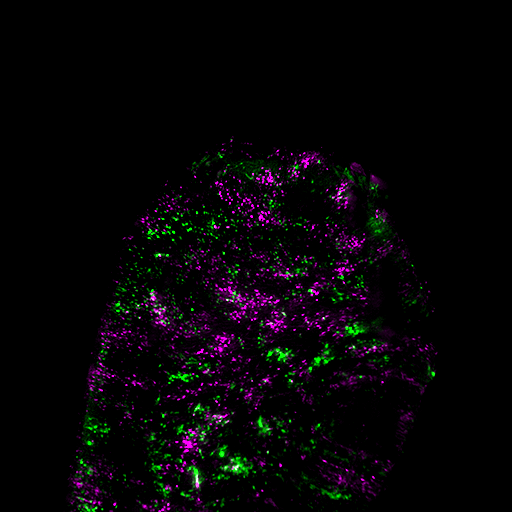

Supplement: Supplementary file 10 — Source data. [file 41556_2024_1412_MOESM10_ESM.zip › Lindenhoferetal-Fig-ED2-sourcedata-NCB/Lindenhoferetal-Fig-ED2f-images-NCB/E10toE17-RSFGGTT-z001.tif]

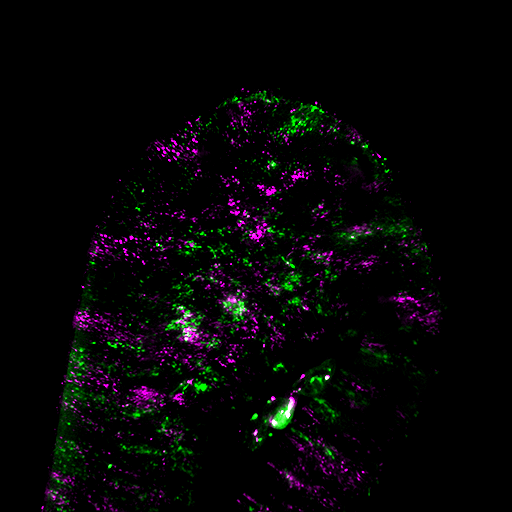

Supplement: Supplementary file 10 — Source data. [file 41556_2024_1412_MOESM10_ESM.zip › Lindenhoferetal-Fig-ED2-sourcedata-NCB/Lindenhoferetal-Fig-ED2f-images-NCB/E10toE17-RSFGGTT-z200.tif]

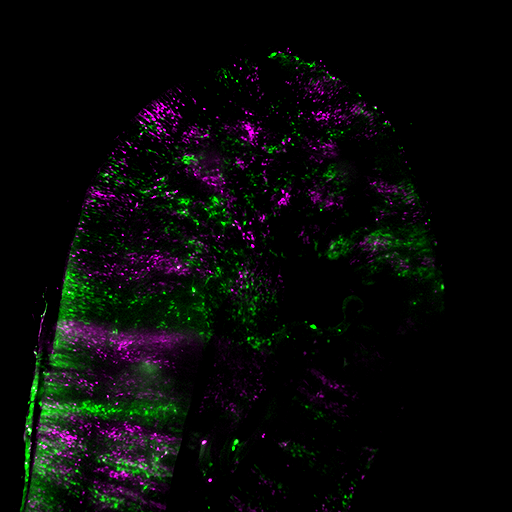

Supplement: Supplementary file 10 — Source data. [file 41556_2024_1412_MOESM10_ESM.zip › Lindenhoferetal-Fig-ED2-sourcedata-NCB/Lindenhoferetal-Fig-ED2f-images-NCB/E10toE17-RSFGGTT-z420.tif]

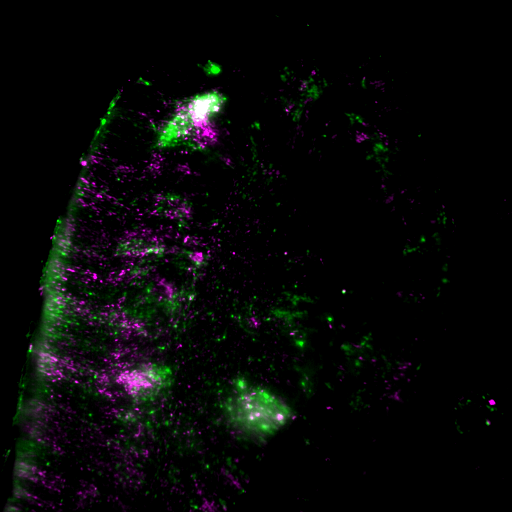

Supplement: Supplementary file 10 — Source data. [file 41556_2024_1412_MOESM10_ESM.zip › Lindenhoferetal-Fig-ED2-sourcedata-NCB/Lindenhoferetal-Fig-ED2f-images-NCB/E10toE17-RSFGGTT-z800.tif]

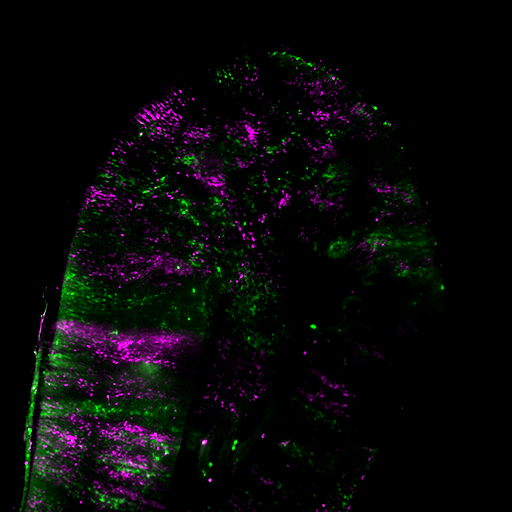

Supplement: Supplementary file 10 — Source data. [file 41556_2024_1412_MOESM10_ESM.zip › Lindenhoferetal-Fig-ED2-sourcedata-NCB/Lindenhoferetal-Fig-ED2f-images-NCB/E10toE17-RSFGGTT-z430.tif]

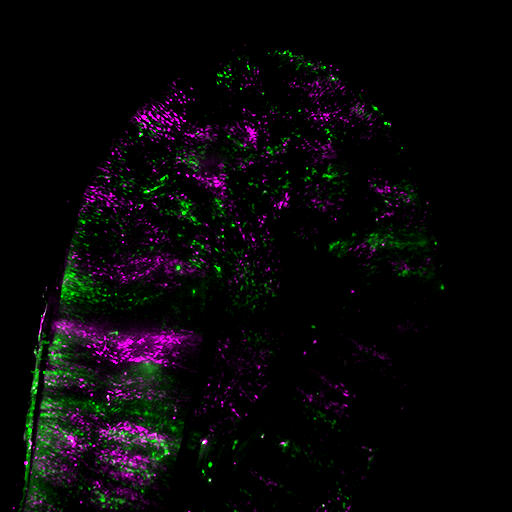

Supplement: Supplementary file 10 — Source data. [file 41556_2024_1412_MOESM10_ESM.zip › Lindenhoferetal-Fig-ED2-sourcedata-NCB/Lindenhoferetal-Fig-ED2f-images-NCB/E10toE17-RSFGGTT-z440.tif]

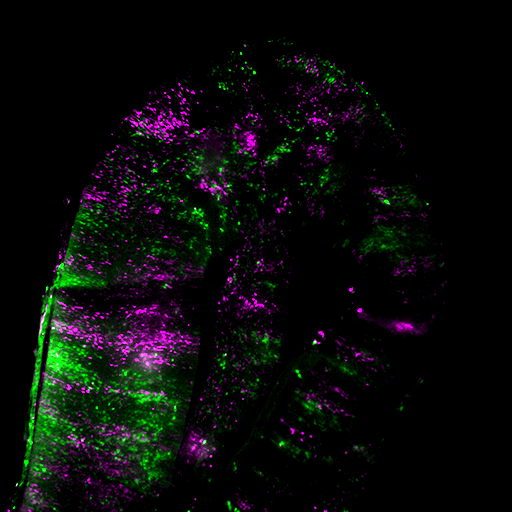

Supplement: Supplementary file 10 — Source data. [file 41556_2024_1412_MOESM10_ESM.zip › Lindenhoferetal-Fig-ED2-sourcedata-NCB/Lindenhoferetal-Fig-ED2f-images-NCB/E10toE17-RSFGGTT-z480.tif]

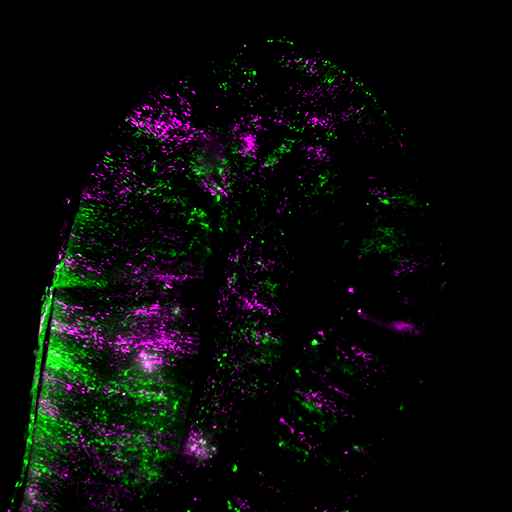

Supplement: Supplementary file 10 — Source data. [file 41556_2024_1412_MOESM10_ESM.zip › Lindenhoferetal-Fig-ED2-sourcedata-NCB/Lindenhoferetal-Fig-ED2f-images-NCB/E10toE17-RSFGGTT-z490.tif]

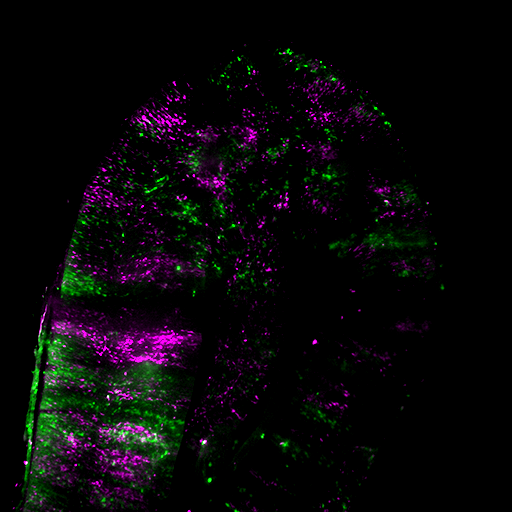

Supplement: Supplementary file 10 — Source data. [file 41556_2024_1412_MOESM10_ESM.zip › Lindenhoferetal-Fig-ED2-sourcedata-NCB/Lindenhoferetal-Fig-ED2f-images-NCB/E10toE17-RSFGGTT-z450.tif]

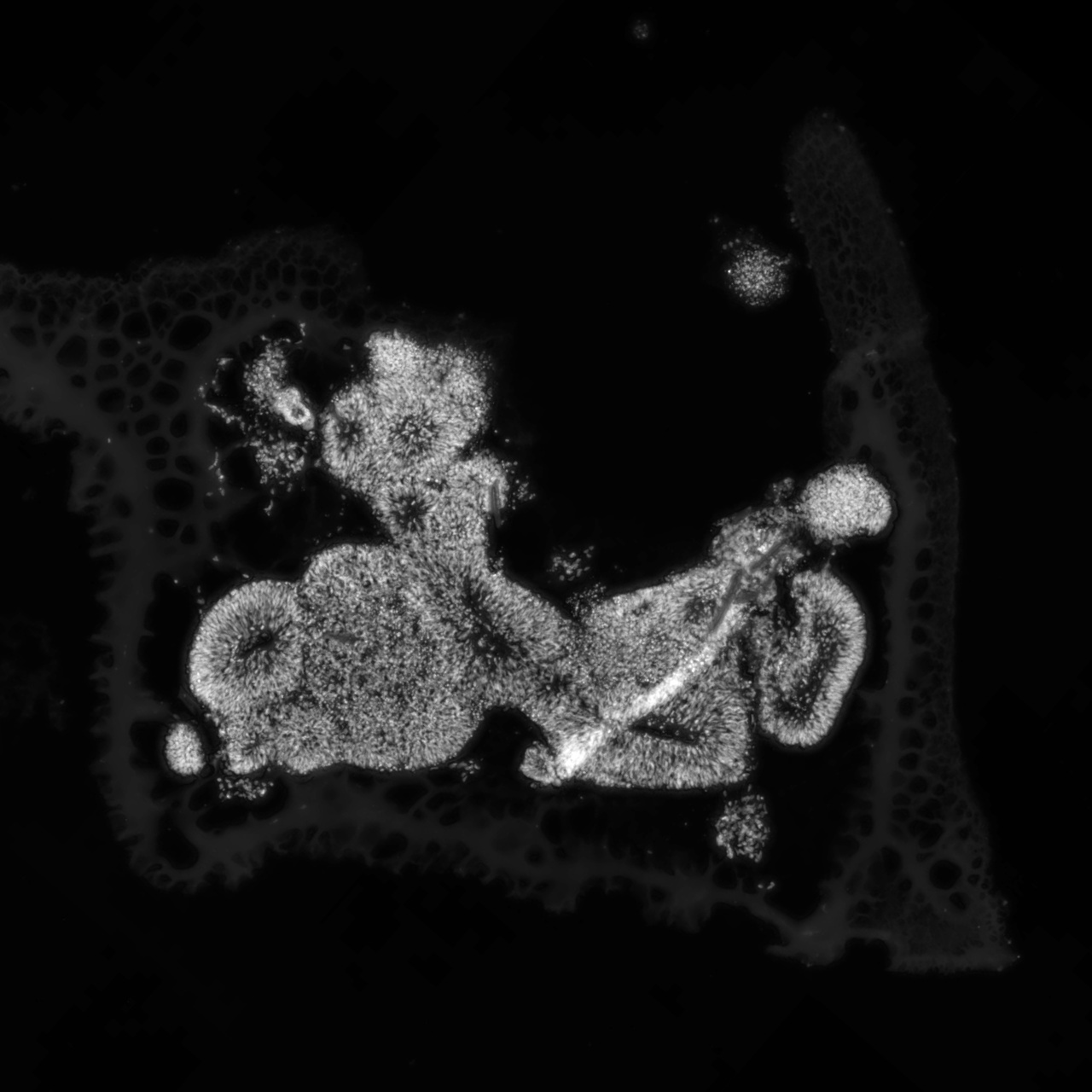

Supplement: Supplementary file 12 — Source data. [file 41556_2024_1412_MOESM12_ESM.zip › Lindenhoferetal-Fig-ED4-sourcedata-NCB/Lindenhoferetal-Fig-ED4-images-NCB/Lindenhoferetal-Fig-ED4c-day16-DAPI.tif]

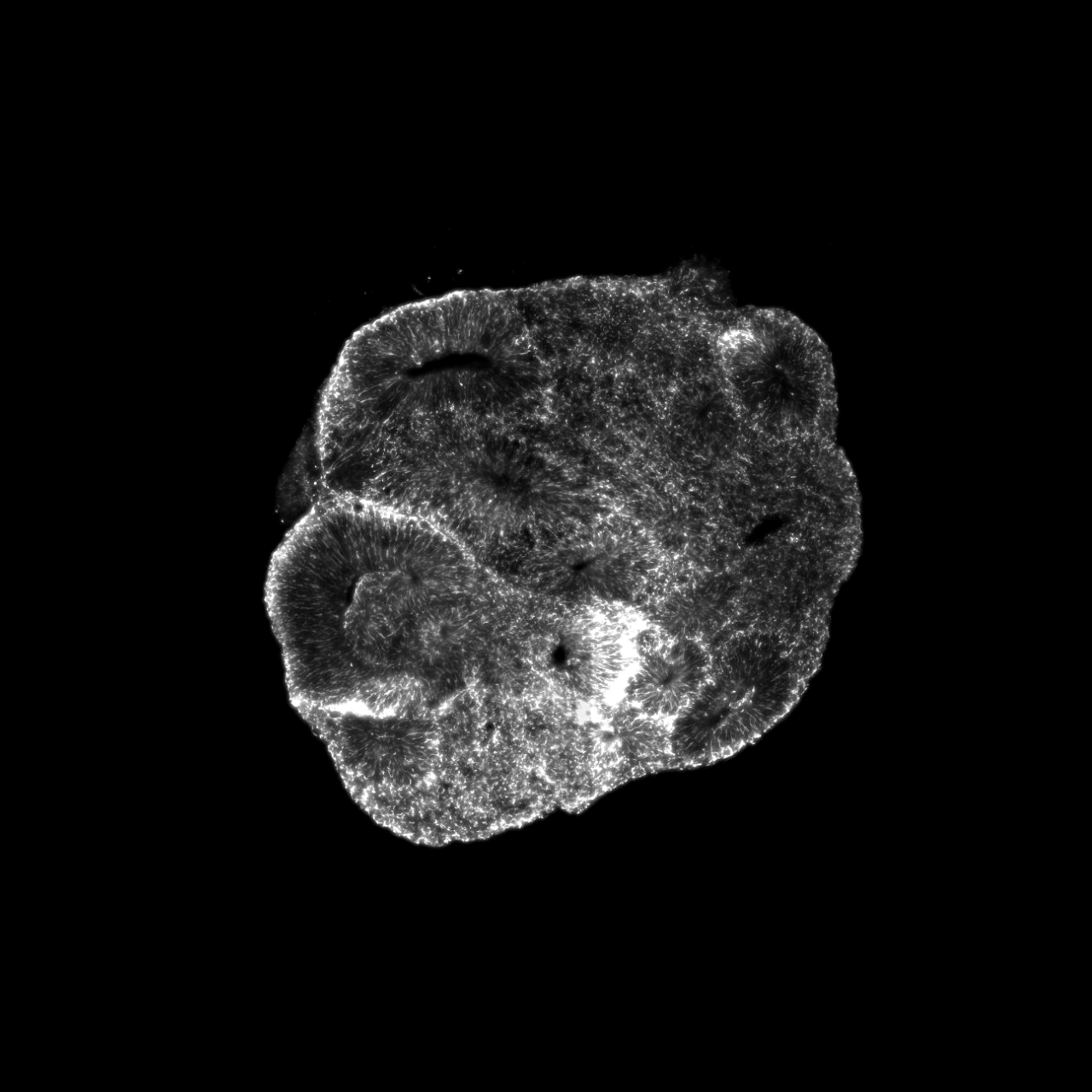

Supplement: Supplementary file 12 — Source data. [file 41556_2024_1412_MOESM12_ESM.zip › Lindenhoferetal-Fig-ED4-sourcedata-NCB/Lindenhoferetal-Fig-ED4-images-NCB/Lindenhoferetal-Fig-ED4c-day25-nestin.tif]

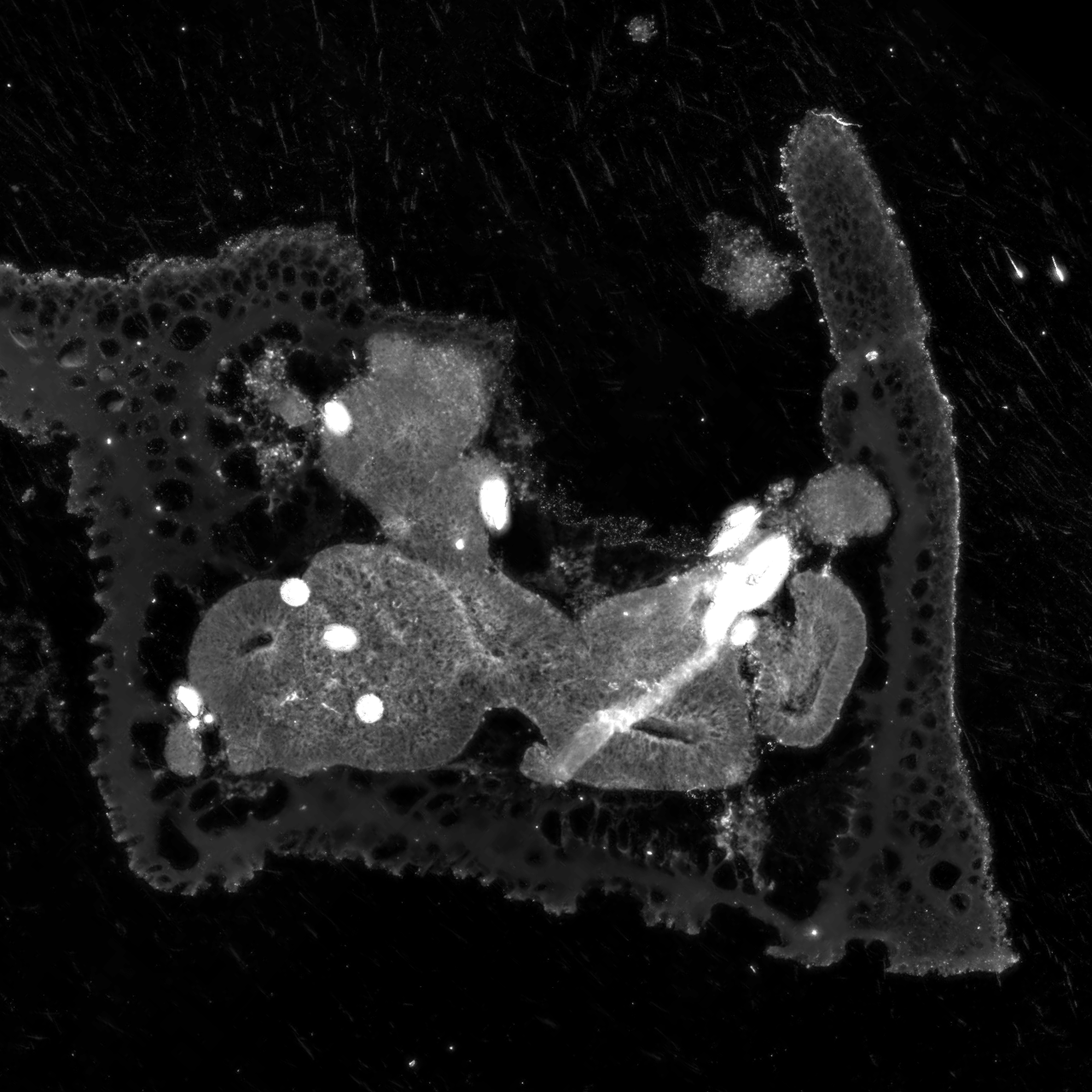

Supplement: Supplementary file 12 — Source data. [file 41556_2024_1412_MOESM12_ESM.zip › Lindenhoferetal-Fig-ED4-sourcedata-NCB/Lindenhoferetal-Fig-ED4-images-NCB/Lindenhoferetal-Fig-ED4c-day16-MAP2.tif]

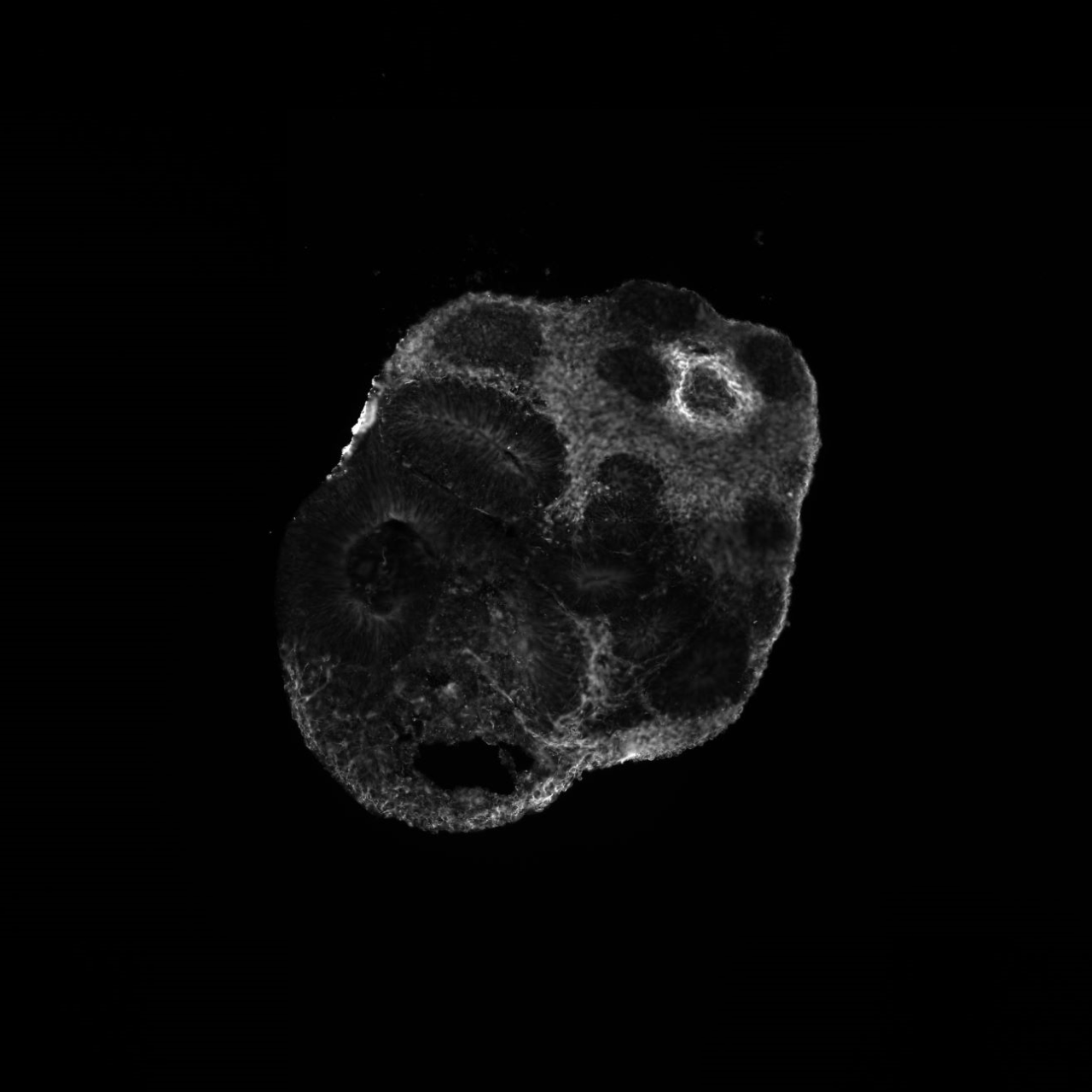

Supplement: Supplementary file 12 — Source data. [file 41556_2024_1412_MOESM12_ESM.zip › Lindenhoferetal-Fig-ED4-sourcedata-NCB/Lindenhoferetal-Fig-ED4-images-NCB/Lindenhoferetal-Fig-ED4c-day25-DCX.tif]

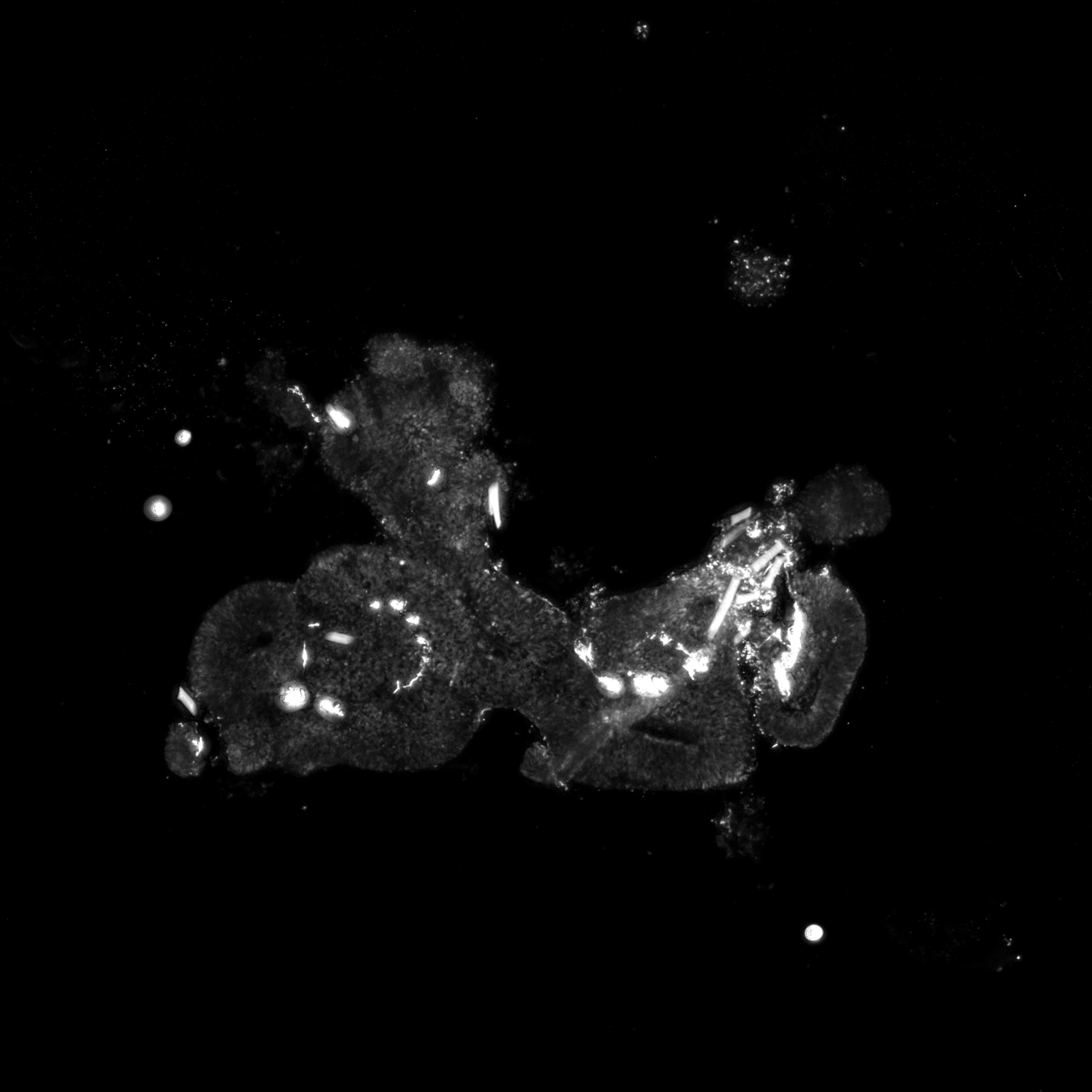

Supplement: Supplementary file 12 — Source data. [file 41556_2024_1412_MOESM12_ESM.zip › Lindenhoferetal-Fig-ED4-sourcedata-NCB/Lindenhoferetal-Fig-ED4-images-NCB/Lindenhoferetal-Fig-ED4c-day16-Sox2.tif]

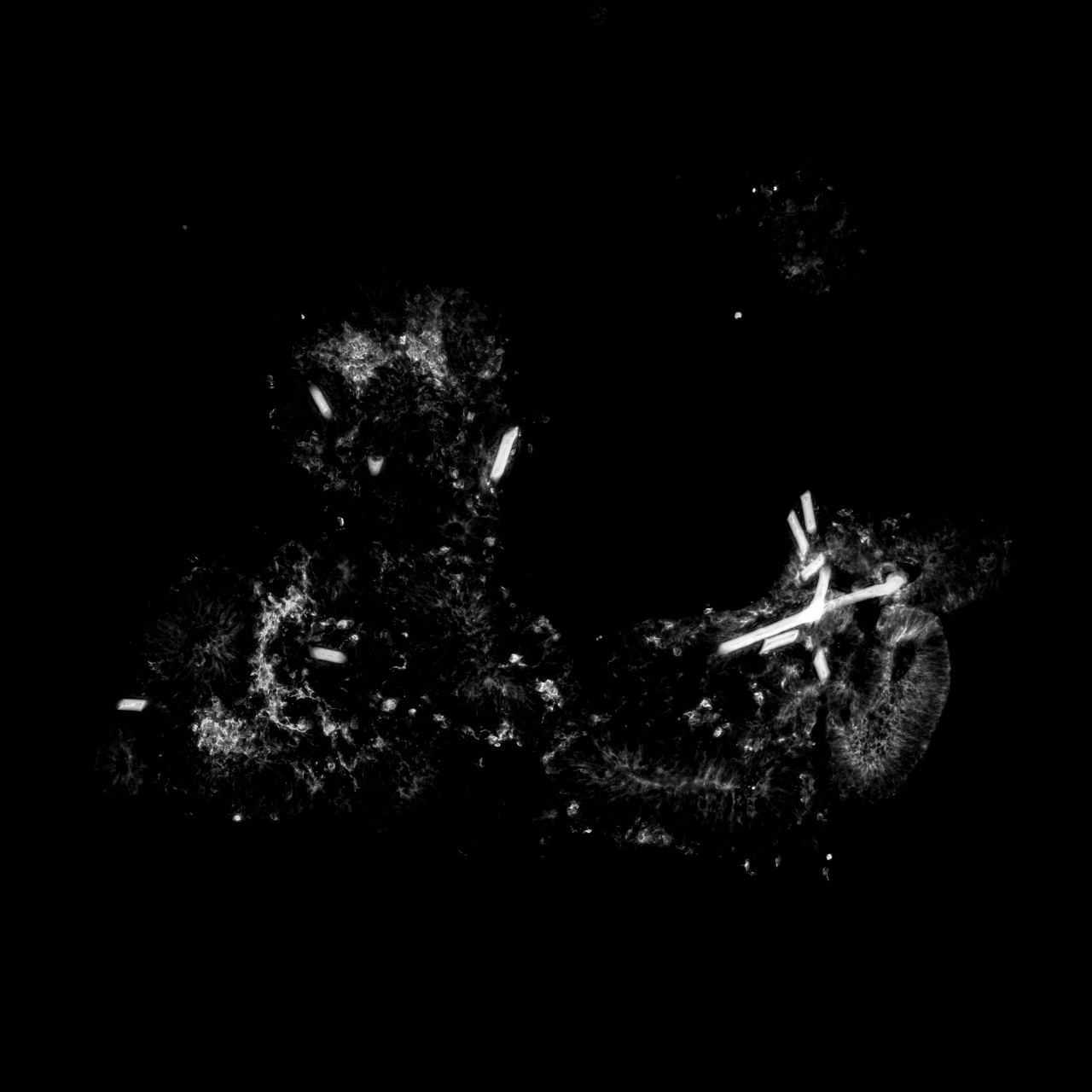

Supplement: Supplementary file 12 — Source data. [file 41556_2024_1412_MOESM12_ESM.zip › Lindenhoferetal-Fig-ED4-sourcedata-NCB/Lindenhoferetal-Fig-ED4-images-NCB/Lindenhoferetal-Fig-ED4c-day16-TuJI.tif]

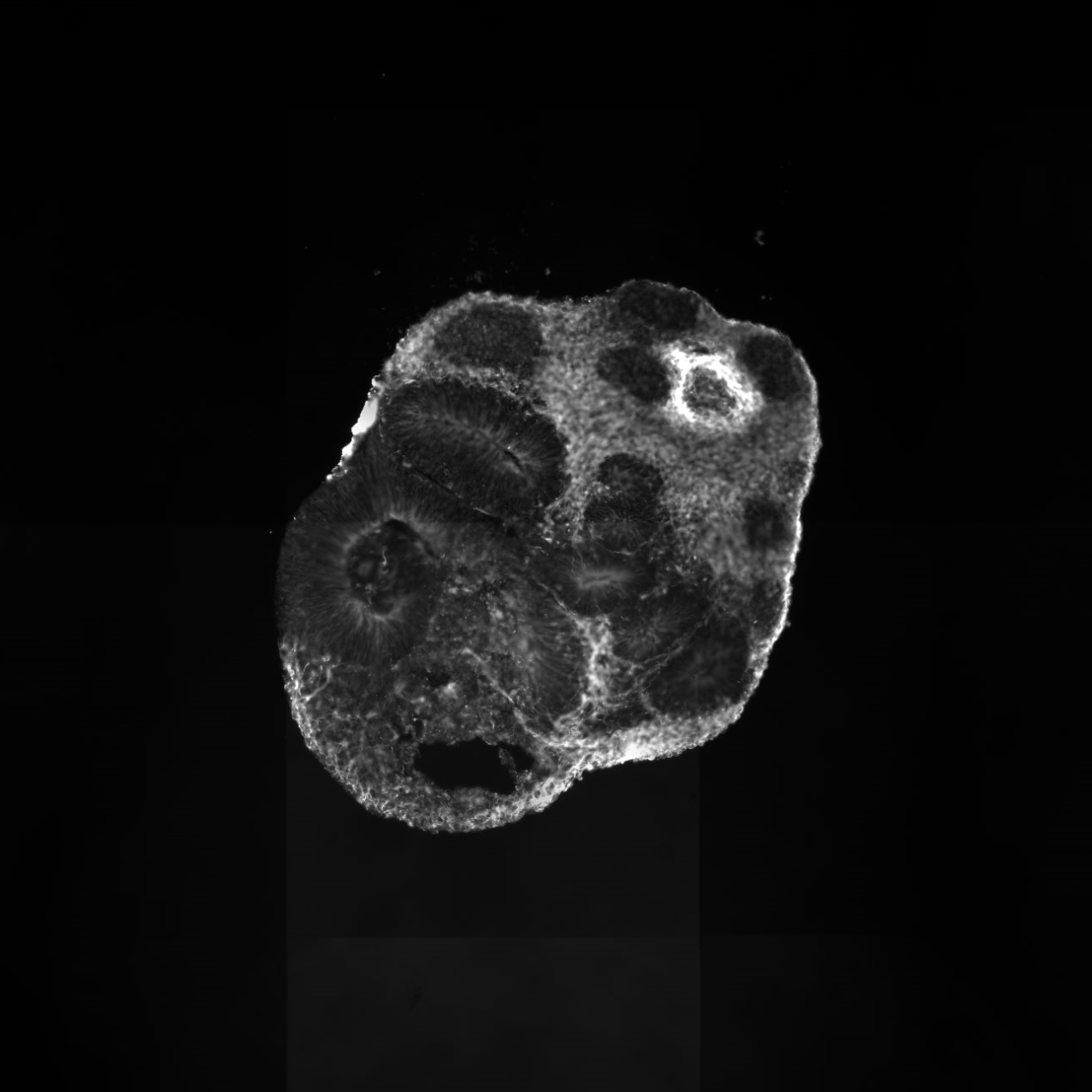

Supplement: Supplementary file 12 — Source data. [file 41556_2024_1412_MOESM12_ESM.zip › Lindenhoferetal-Fig-ED4-sourcedata-NCB/Lindenhoferetal-Fig-ED4-images-NCB/Lindenhoferetal-Fig-ED4c-day25-TuJI.tif]

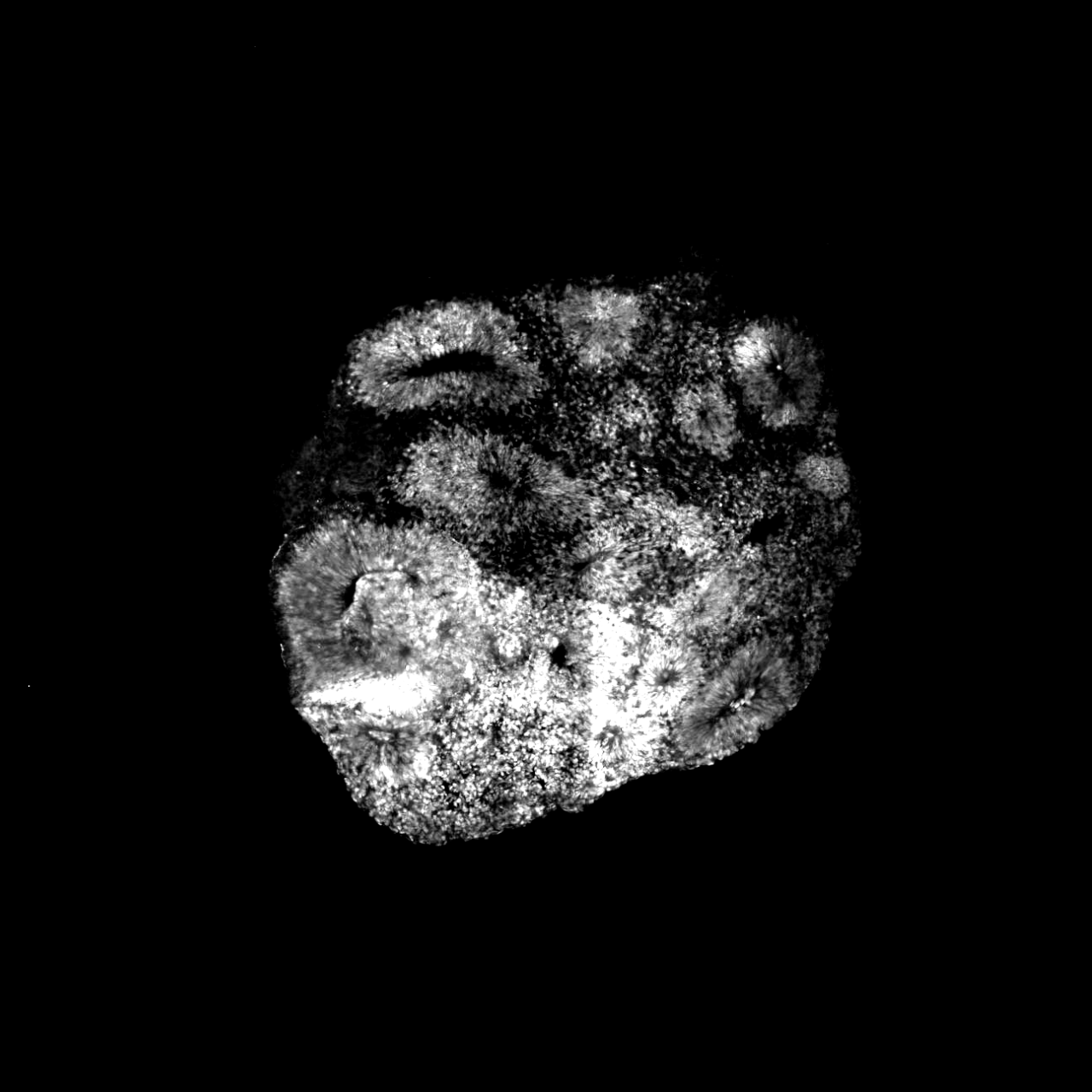

Supplement: Supplementary file 12 — Source data. [file 41556_2024_1412_MOESM12_ESM.zip › Lindenhoferetal-Fig-ED4-sourcedata-NCB/Lindenhoferetal-Fig-ED4-images-NCB/Lindenhoferetal-Fig-ED4c-day25-Sox2.tif]

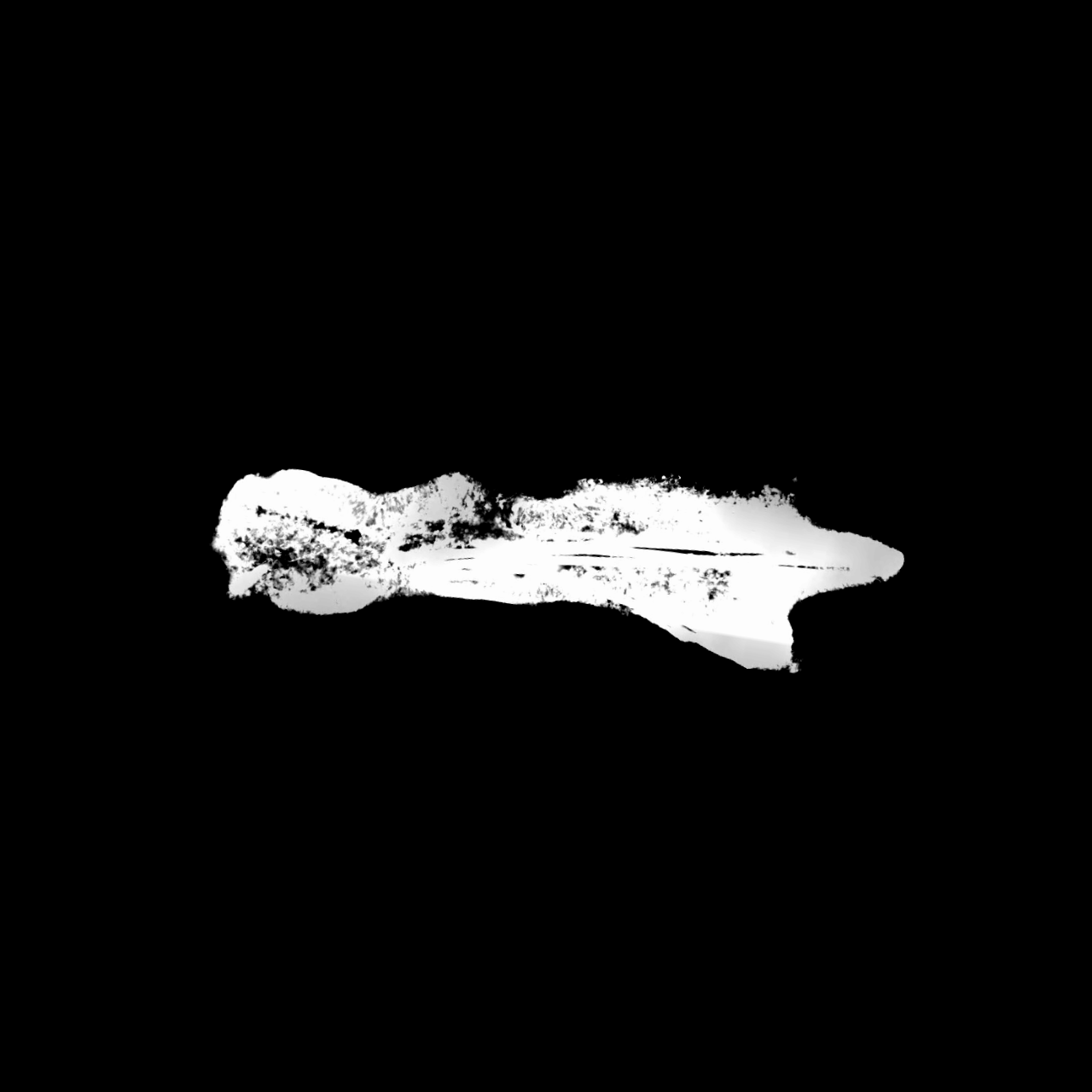

Supplement: Supplementary file 12 — Source data. [file 41556_2024_1412_MOESM12_ESM.zip › Lindenhoferetal-Fig-ED4-sourcedata-NCB/Lindenhoferetal-Fig-ED4-images-NCB/Lindenhoferetal-Fig-ED4c-day10-nestin.tif]

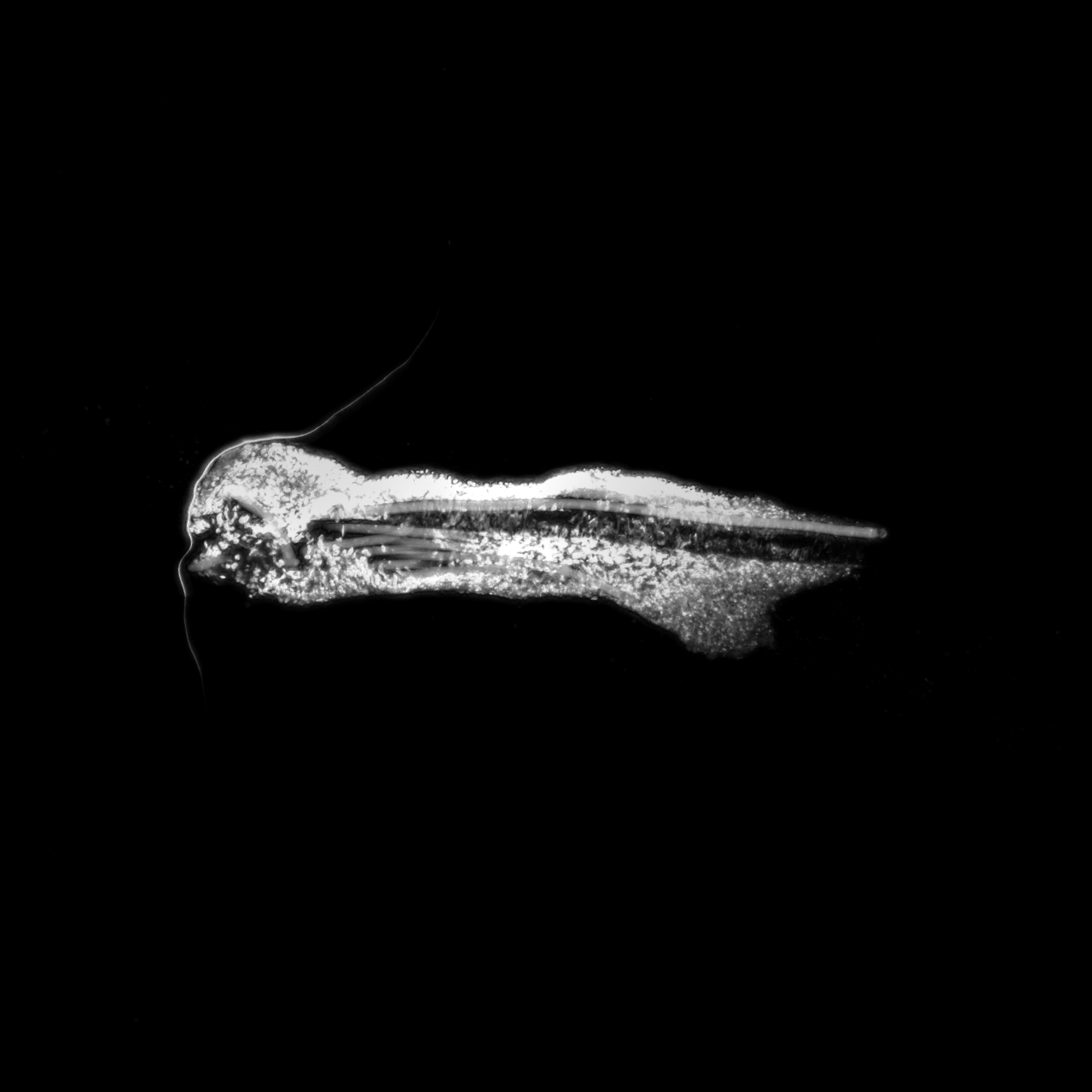

Supplement: Supplementary file 12 — Source data. [file 41556_2024_1412_MOESM12_ESM.zip › Lindenhoferetal-Fig-ED4-sourcedata-NCB/Lindenhoferetal-Fig-ED4-images-NCB/Lindenhoferetal-Fig-ED4c-day10-Sox2.tif]

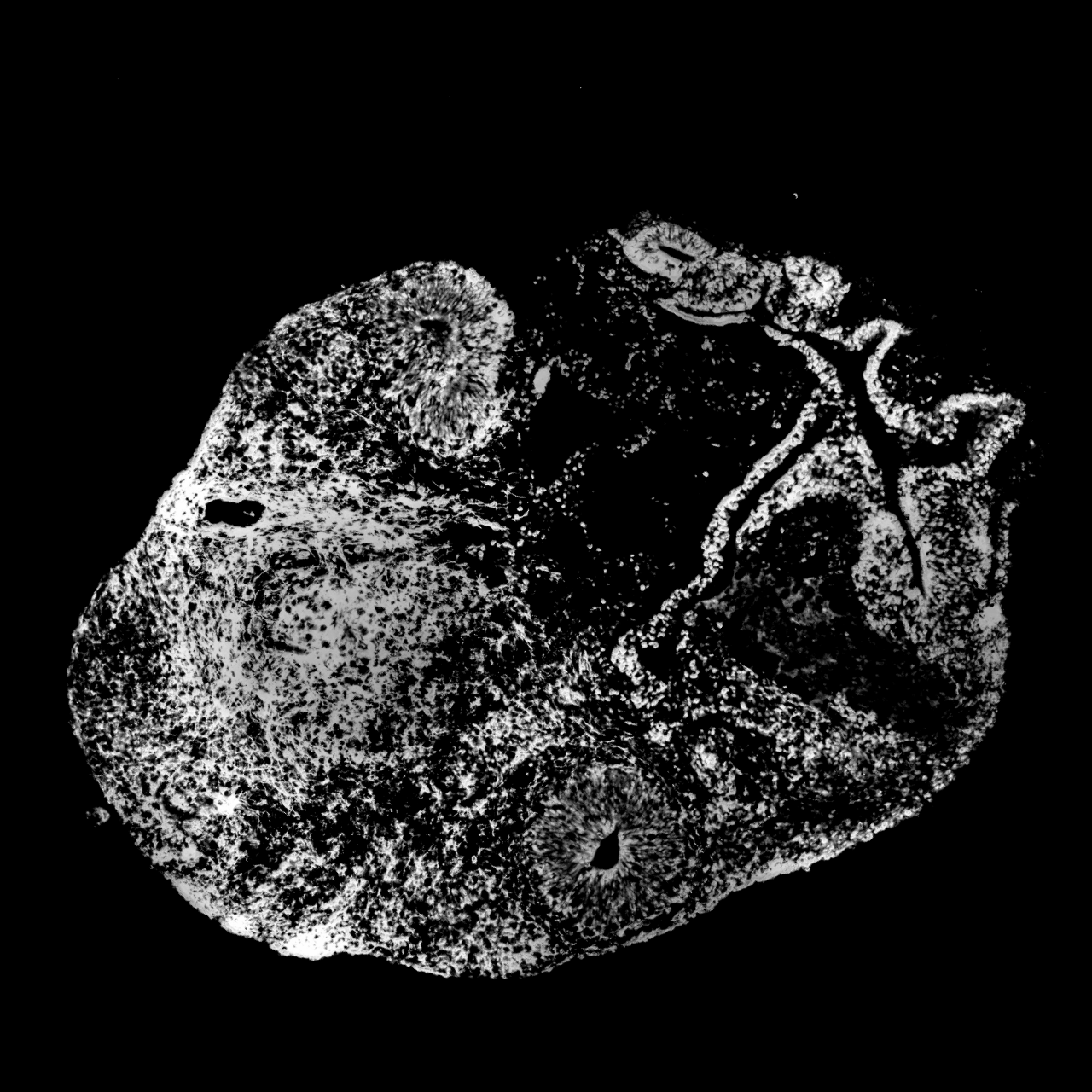

Supplement: Supplementary file 12 — Source data. [file 41556_2024_1412_MOESM12_ESM.zip › Lindenhoferetal-Fig-ED4-sourcedata-NCB/Lindenhoferetal-Fig-ED4-images-NCB/Lindenhoferetal-Fig-ED4c-day33-MAP2.tif]

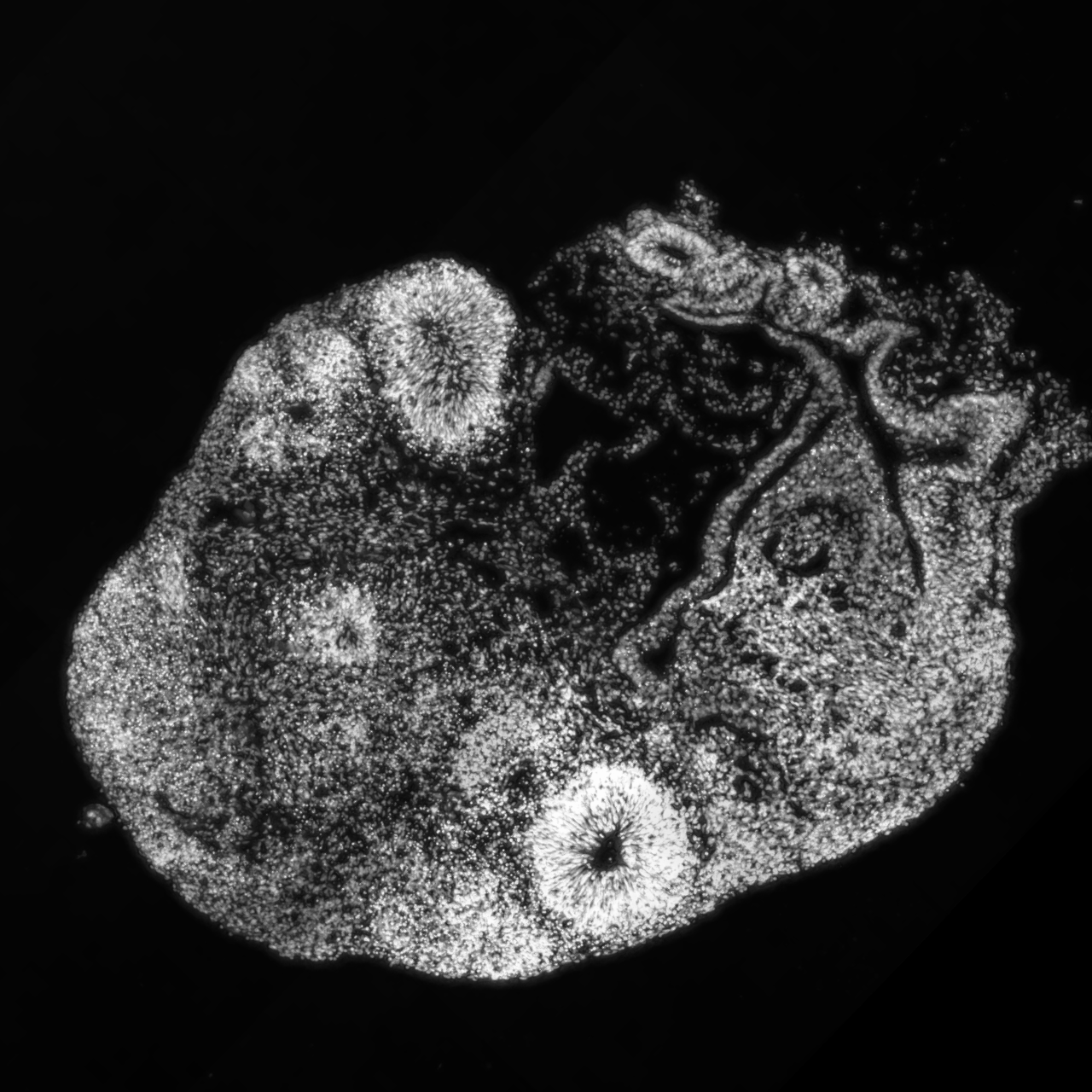

Supplement: Supplementary file 12 — Source data. [file 41556_2024_1412_MOESM12_ESM.zip › Lindenhoferetal-Fig-ED4-sourcedata-NCB/Lindenhoferetal-Fig-ED4-images-NCB/Lindenhoferetal-Fig-ED4c-day33-DAPI.tif]

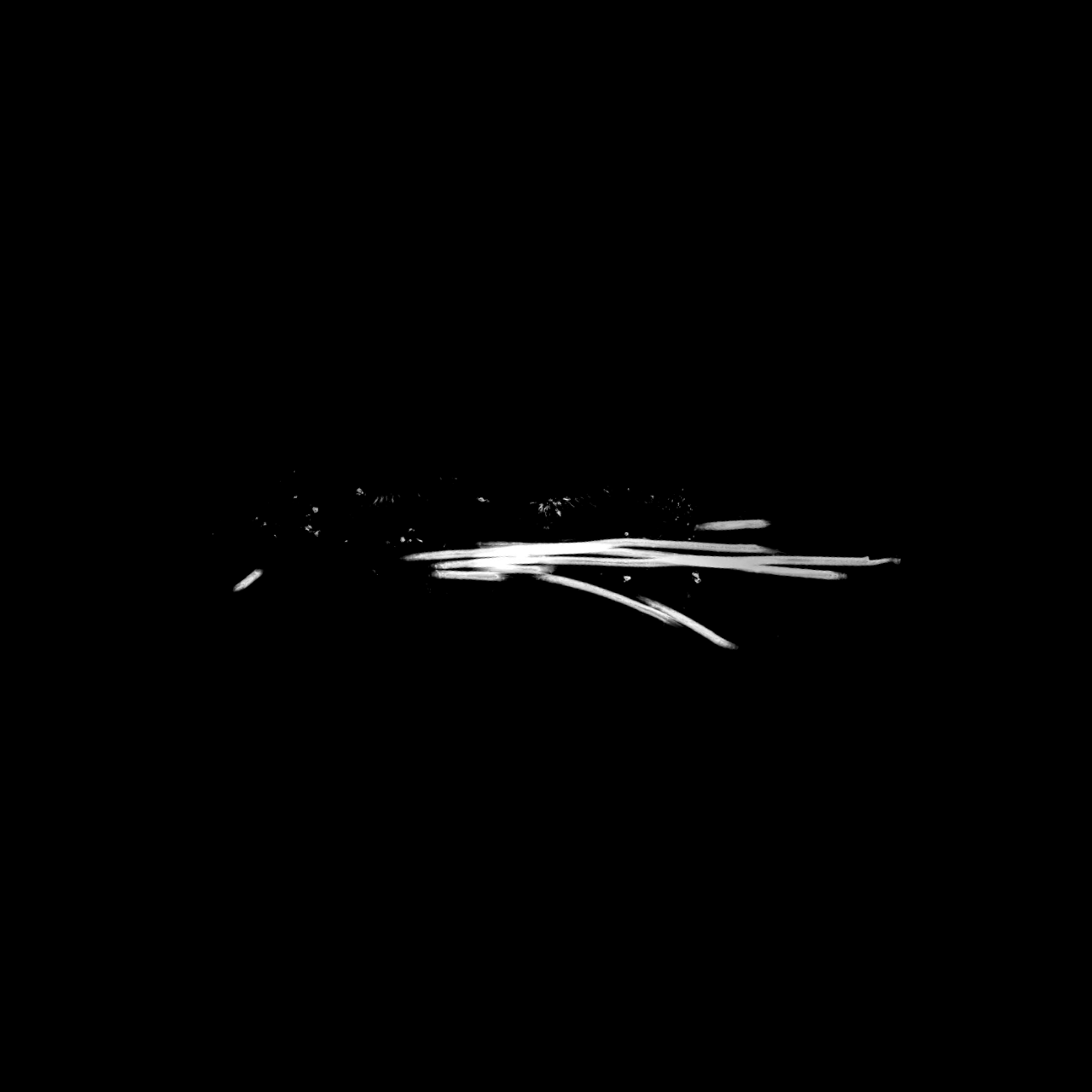

Supplement: Supplementary file 12 — Source data. [file 41556_2024_1412_MOESM12_ESM.zip › Lindenhoferetal-Fig-ED4-sourcedata-NCB/Lindenhoferetal-Fig-ED4-images-NCB/Lindenhoferetal-Fig-ED4c-day10-TuJI.tif]

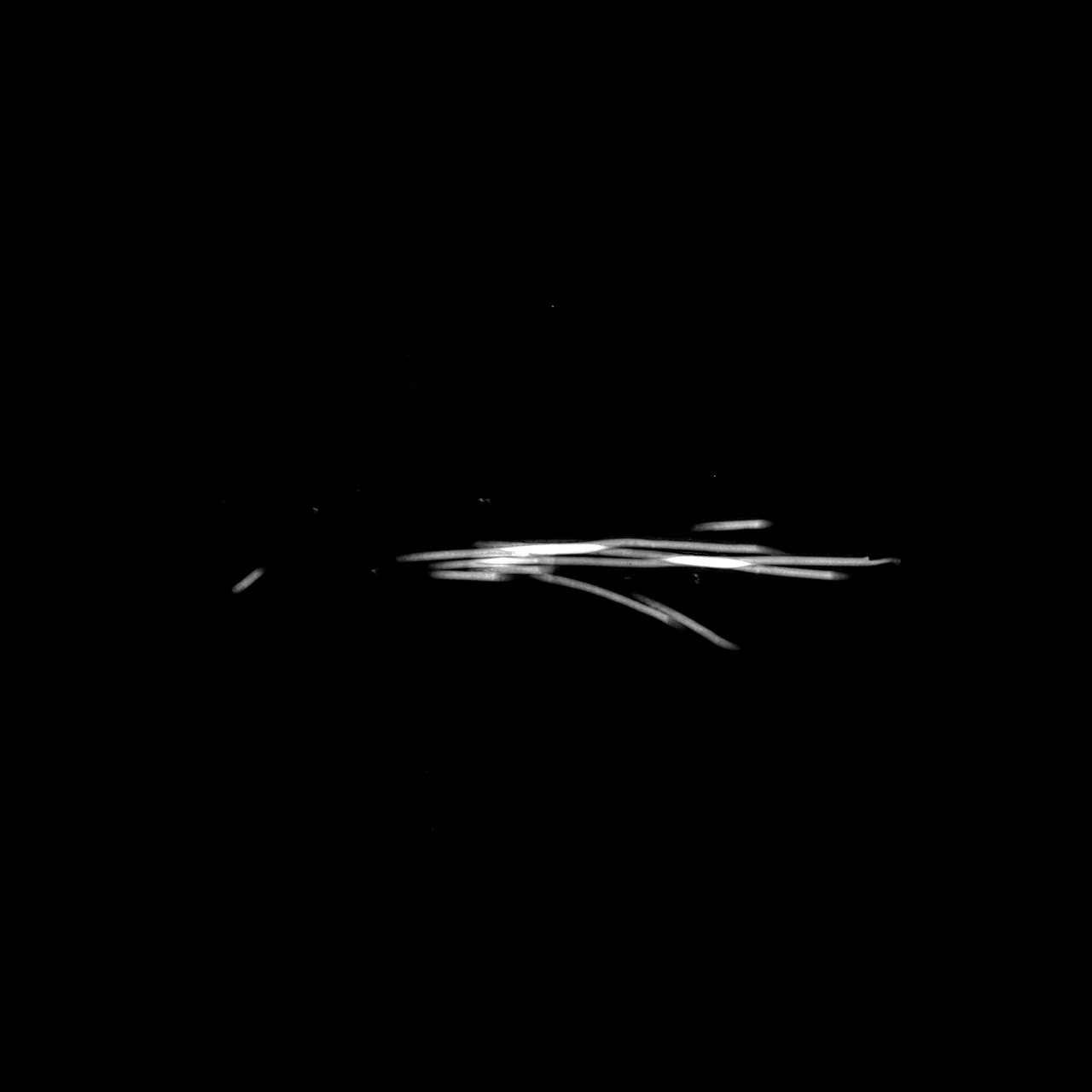

Supplement: Supplementary file 12 — Source data. [file 41556_2024_1412_MOESM12_ESM.zip › Lindenhoferetal-Fig-ED4-sourcedata-NCB/Lindenhoferetal-Fig-ED4-images-NCB/Lindenhoferetal-Fig-ED4c-day10-DCX.tif]

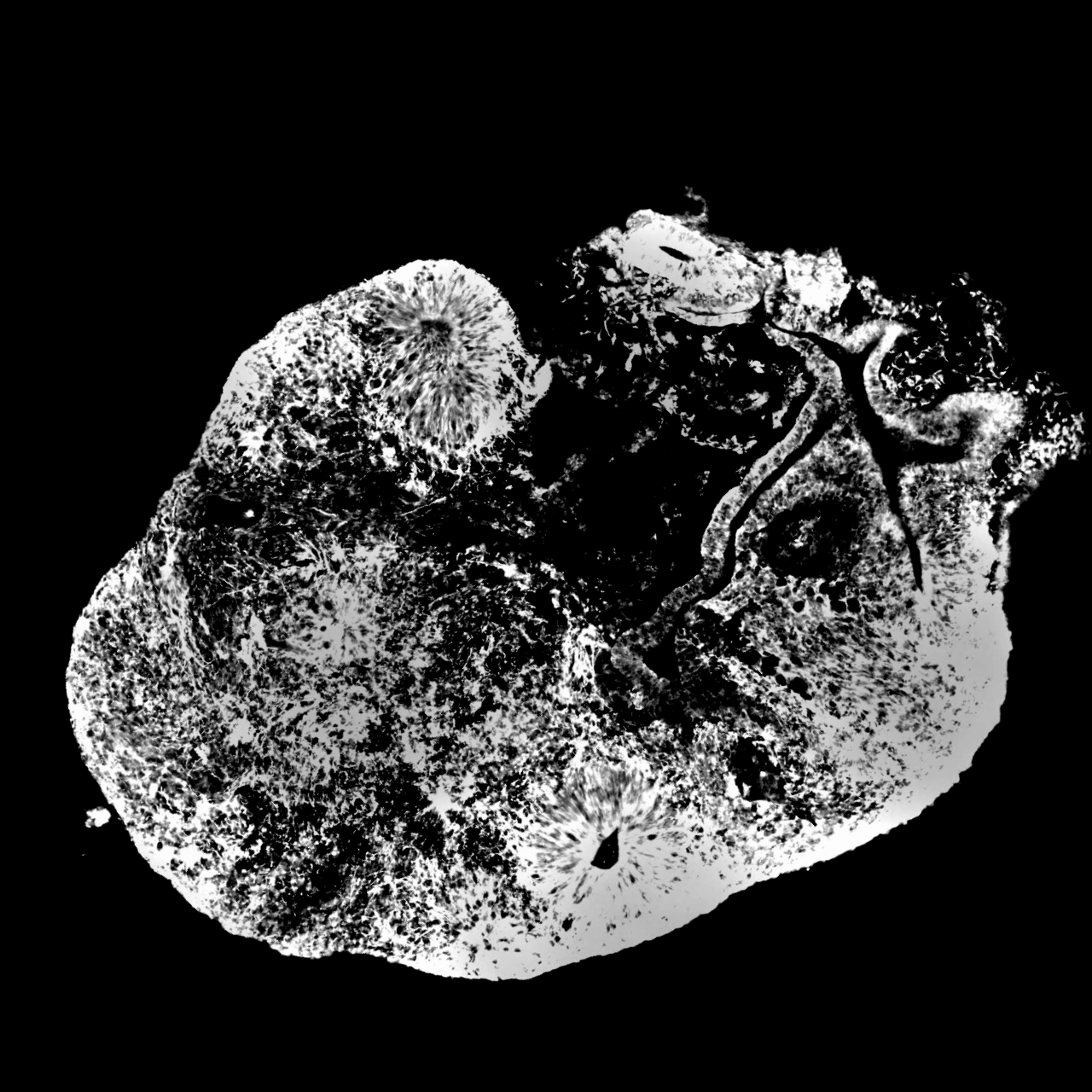

Supplement: Supplementary file 12 — Source data. [file 41556_2024_1412_MOESM12_ESM.zip › Lindenhoferetal-Fig-ED4-sourcedata-NCB/Lindenhoferetal-Fig-ED4-images-NCB/Lindenhoferetal-Fig-ED4c-day33-nestin.tif]

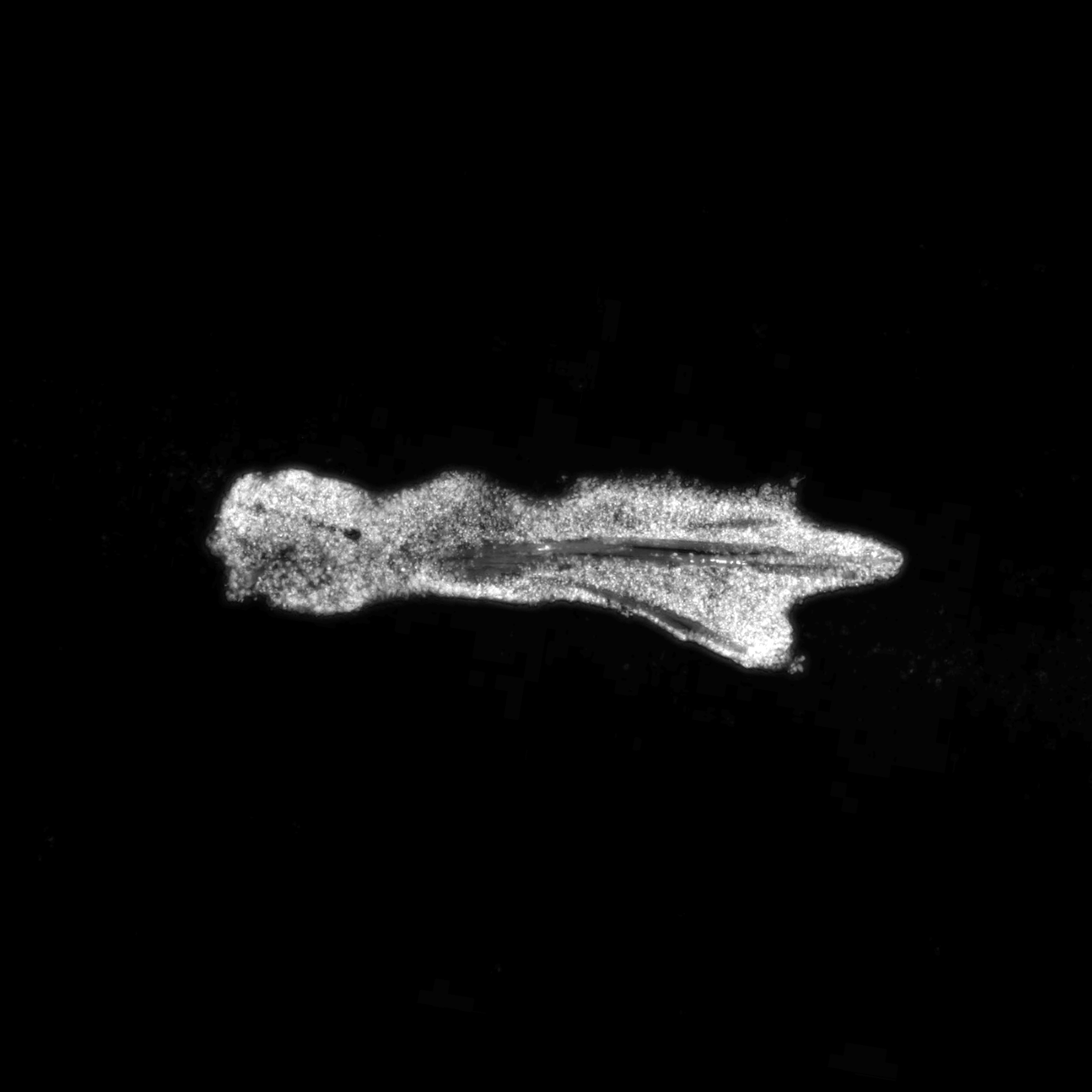

Supplement: Supplementary file 12 — Source data. [file 41556_2024_1412_MOESM12_ESM.zip › Lindenhoferetal-Fig-ED4-sourcedata-NCB/Lindenhoferetal-Fig-ED4-images-NCB/Lindenhoferetal-Fig-ED4c-day10-DAPI.tif]

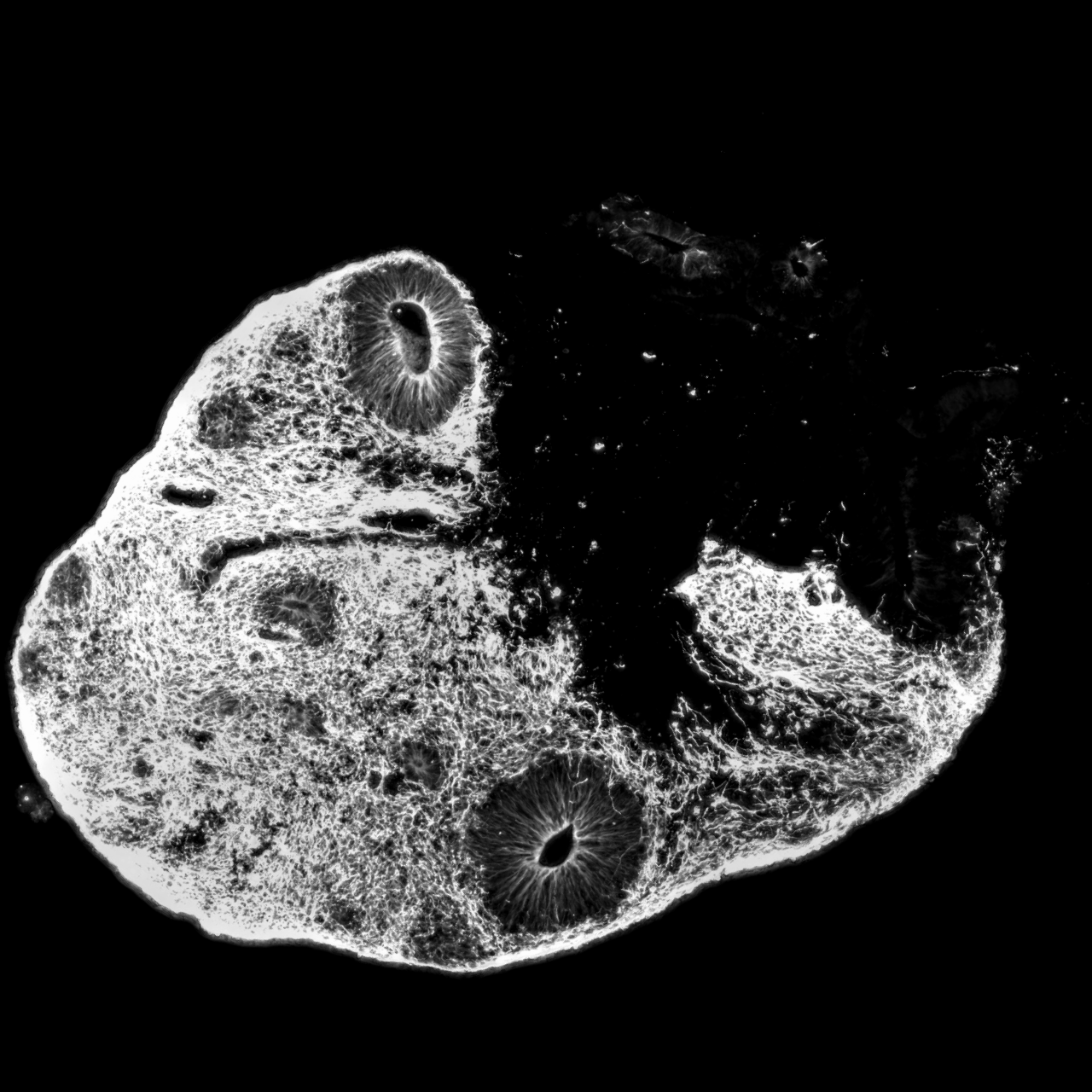

Supplement: Supplementary file 12 — Source data. [file 41556_2024_1412_MOESM12_ESM.zip › Lindenhoferetal-Fig-ED4-sourcedata-NCB/Lindenhoferetal-Fig-ED4-images-NCB/Lindenhoferetal-Fig-ED4c-day33-TuJI.tif]

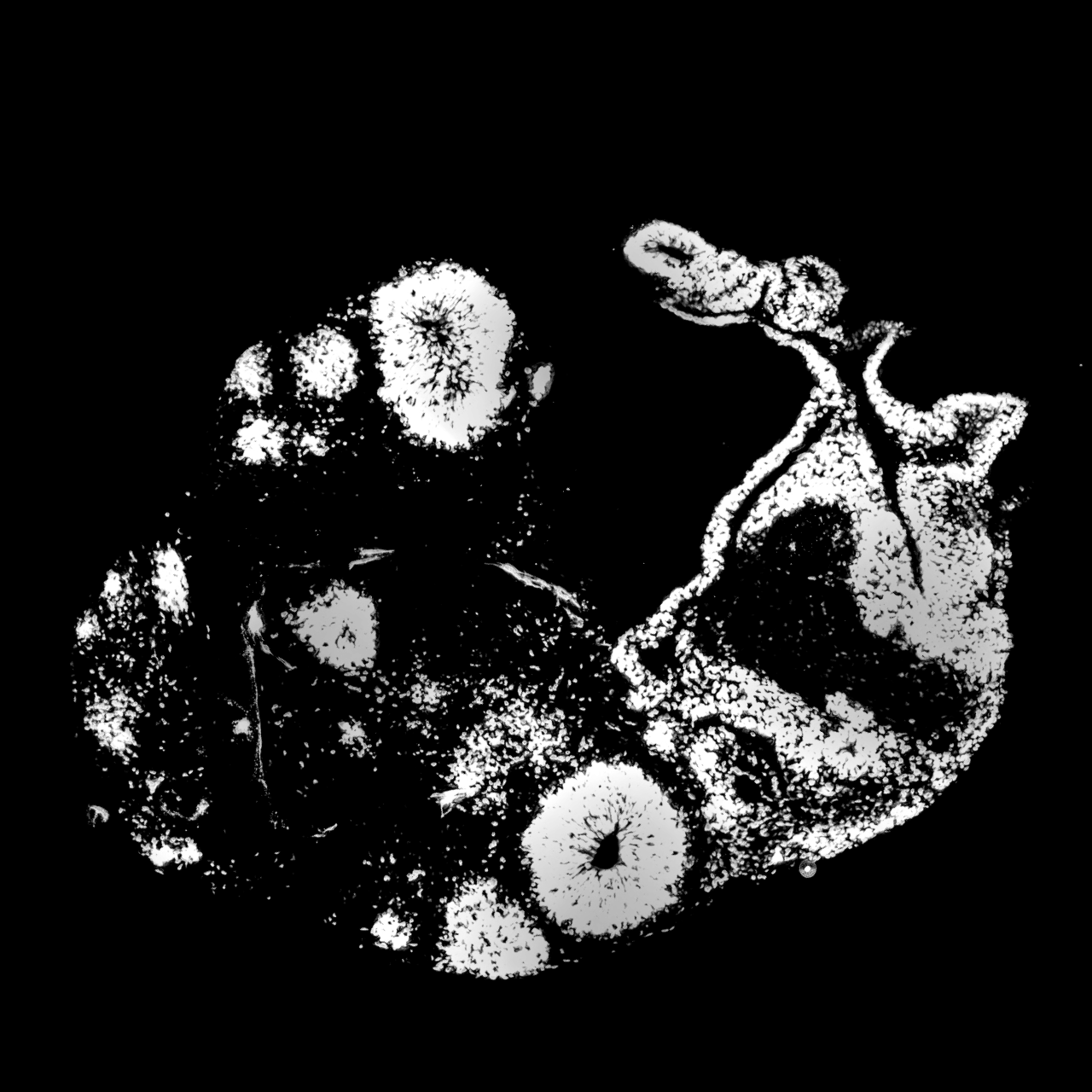

Supplement: Supplementary file 12 — Source data. [file 41556_2024_1412_MOESM12_ESM.zip › Lindenhoferetal-Fig-ED4-sourcedata-NCB/Lindenhoferetal-Fig-ED4-images-NCB/Lindenhoferetal-Fig-ED4c-day33-Sox2.tif]

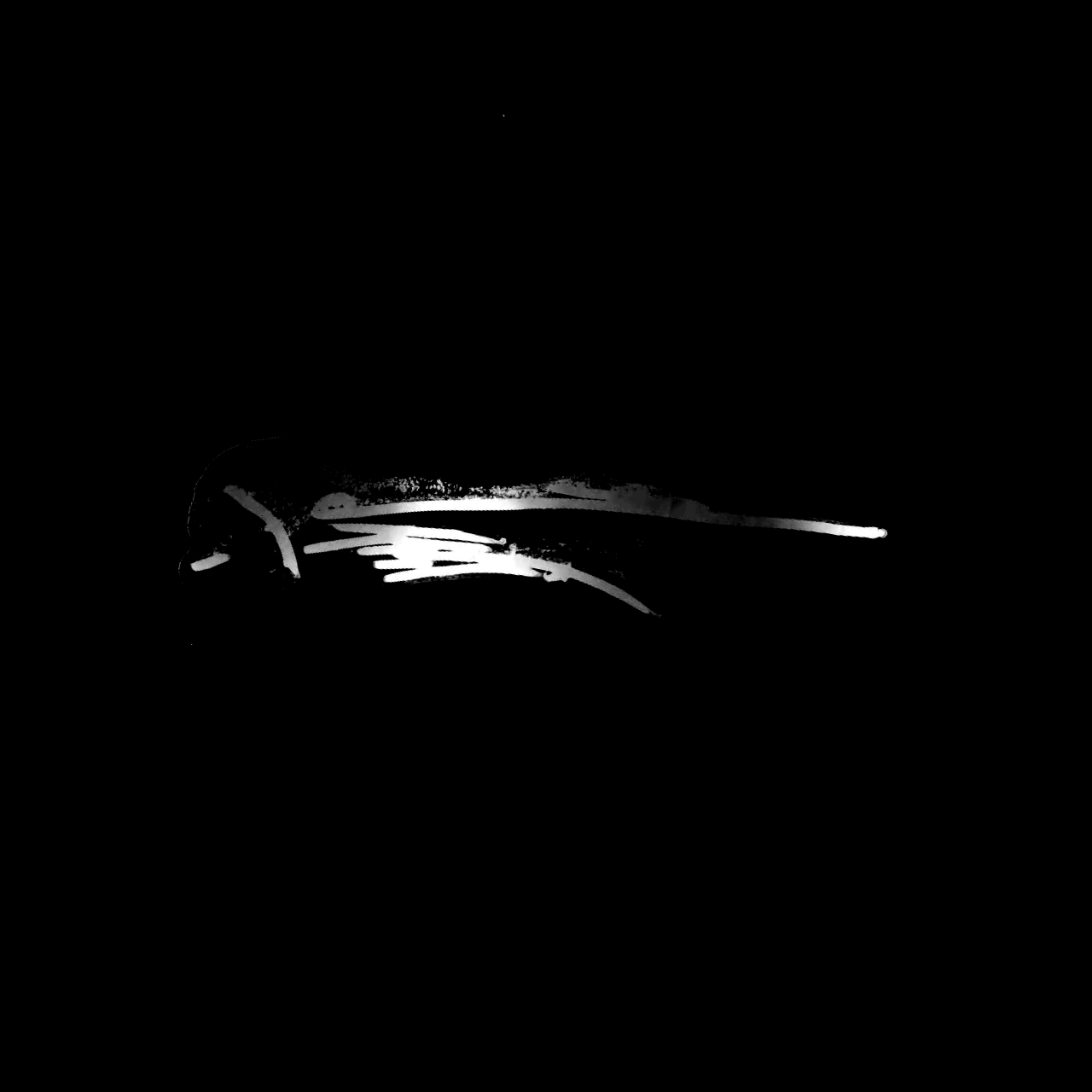

Supplement: Supplementary file 12 — Source data. [file 41556_2024_1412_MOESM12_ESM.zip › Lindenhoferetal-Fig-ED4-sourcedata-NCB/Lindenhoferetal-Fig-ED4-images-NCB/Lindenhoferetal-Fig-ED4c-day10-MAP2.tif]

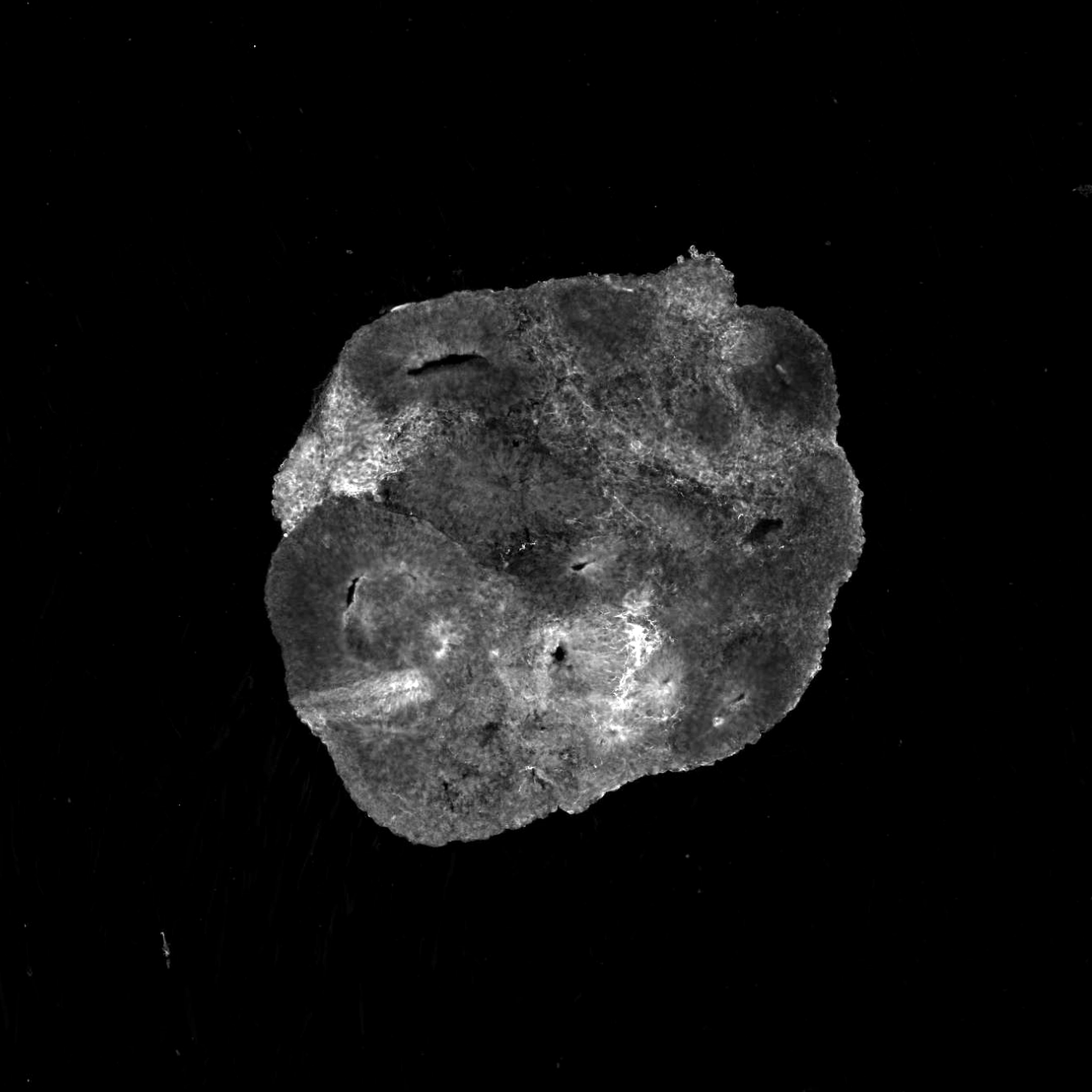

Supplement: Supplementary file 12 — Source data. [file 41556_2024_1412_MOESM12_ESM.zip › Lindenhoferetal-Fig-ED4-sourcedata-NCB/Lindenhoferetal-Fig-ED4-images-NCB/Lindenhoferetal-Fig-ED4c-day25-MAP2.tif]

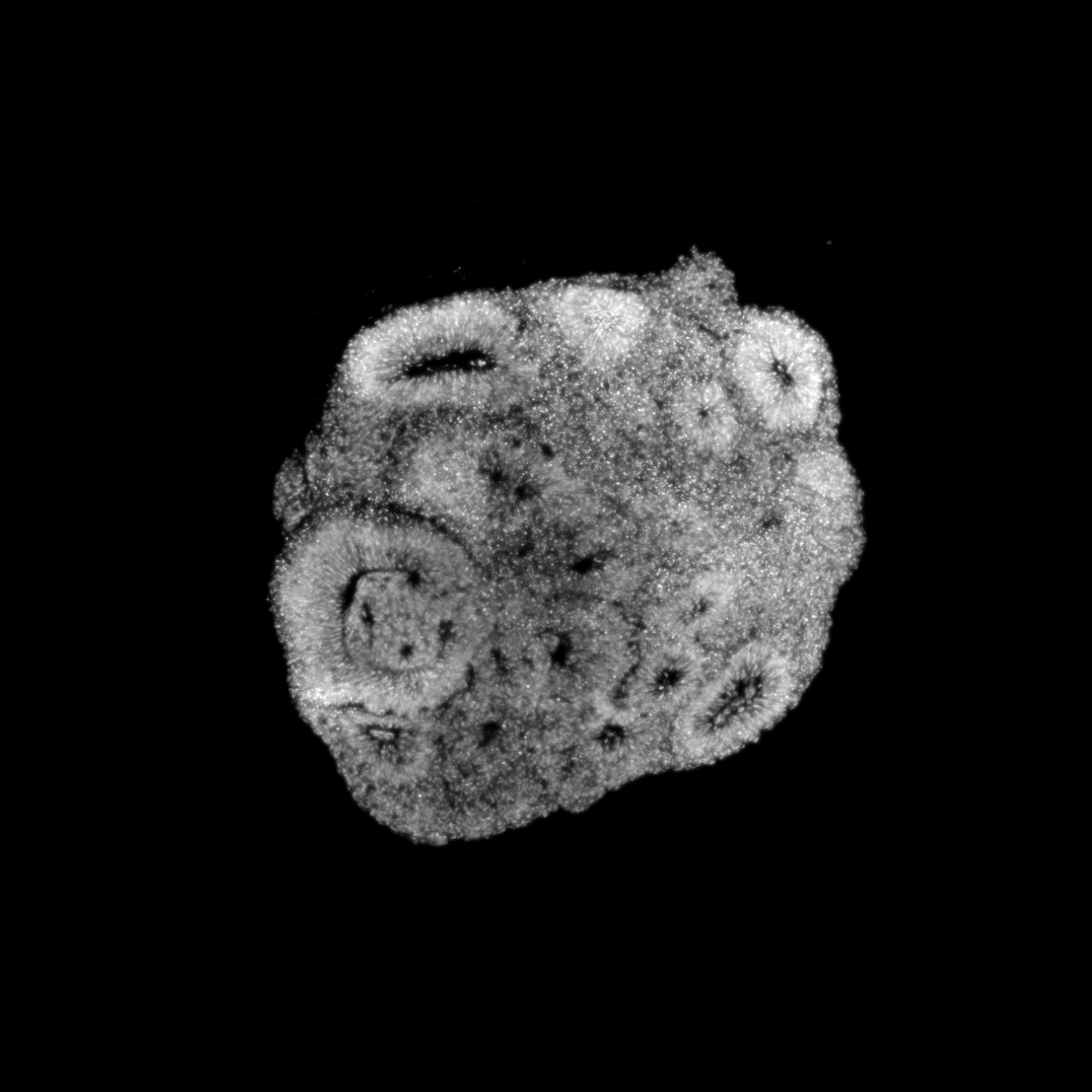

Supplement: Supplementary file 12 — Source data. [file 41556_2024_1412_MOESM12_ESM.zip › Lindenhoferetal-Fig-ED4-sourcedata-NCB/Lindenhoferetal-Fig-ED4-images-NCB/Lindenhoferetal-Fig-ED4c-day25-DAPI.tif]

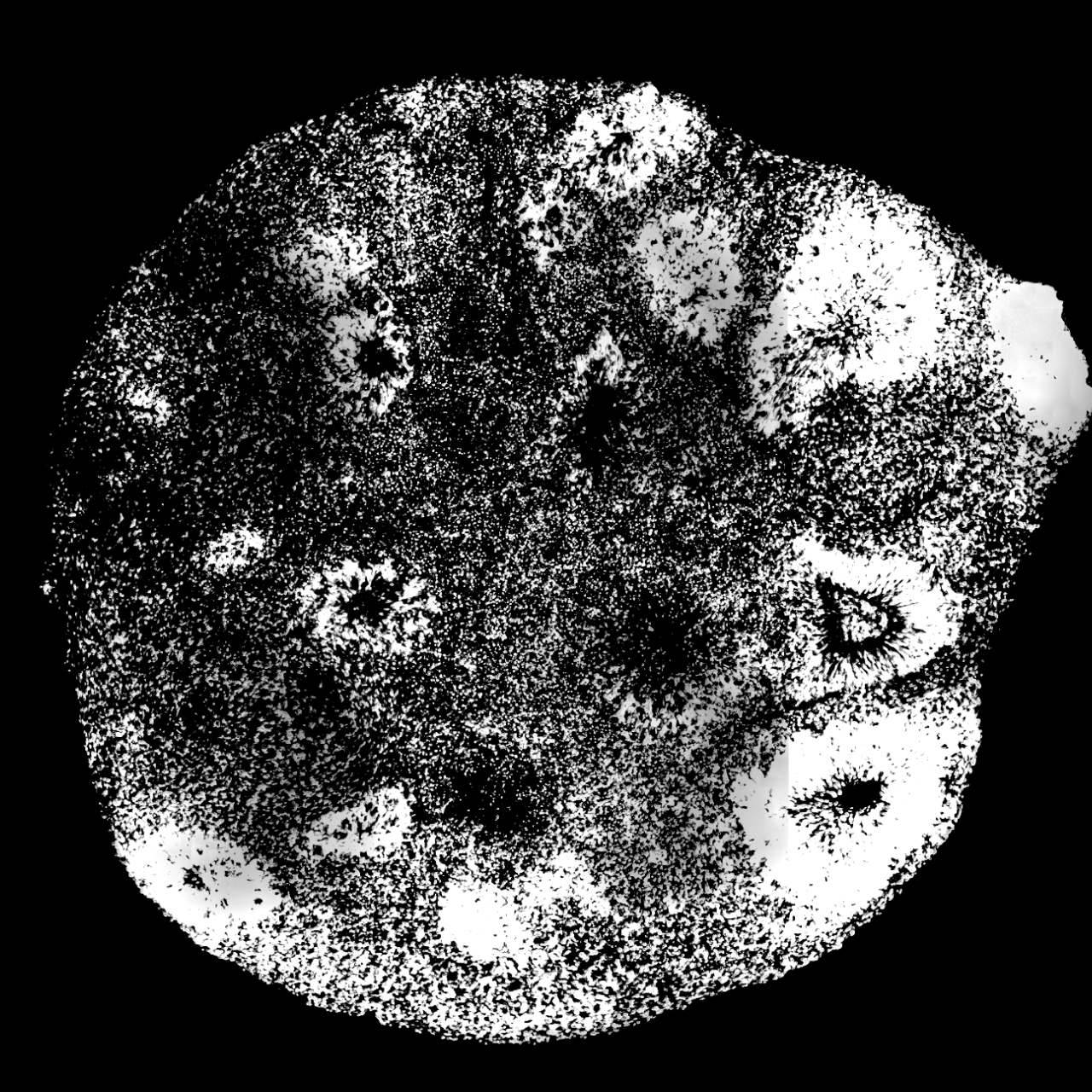

Supplement: Supplementary file 12 — Source data. [file 41556_2024_1412_MOESM12_ESM.zip › Lindenhoferetal-Fig-ED4-sourcedata-NCB/Lindenhoferetal-Fig-ED4-images-NCB/Lindenhoferetal-Fig-ED4c-day41-DAPI.tif]

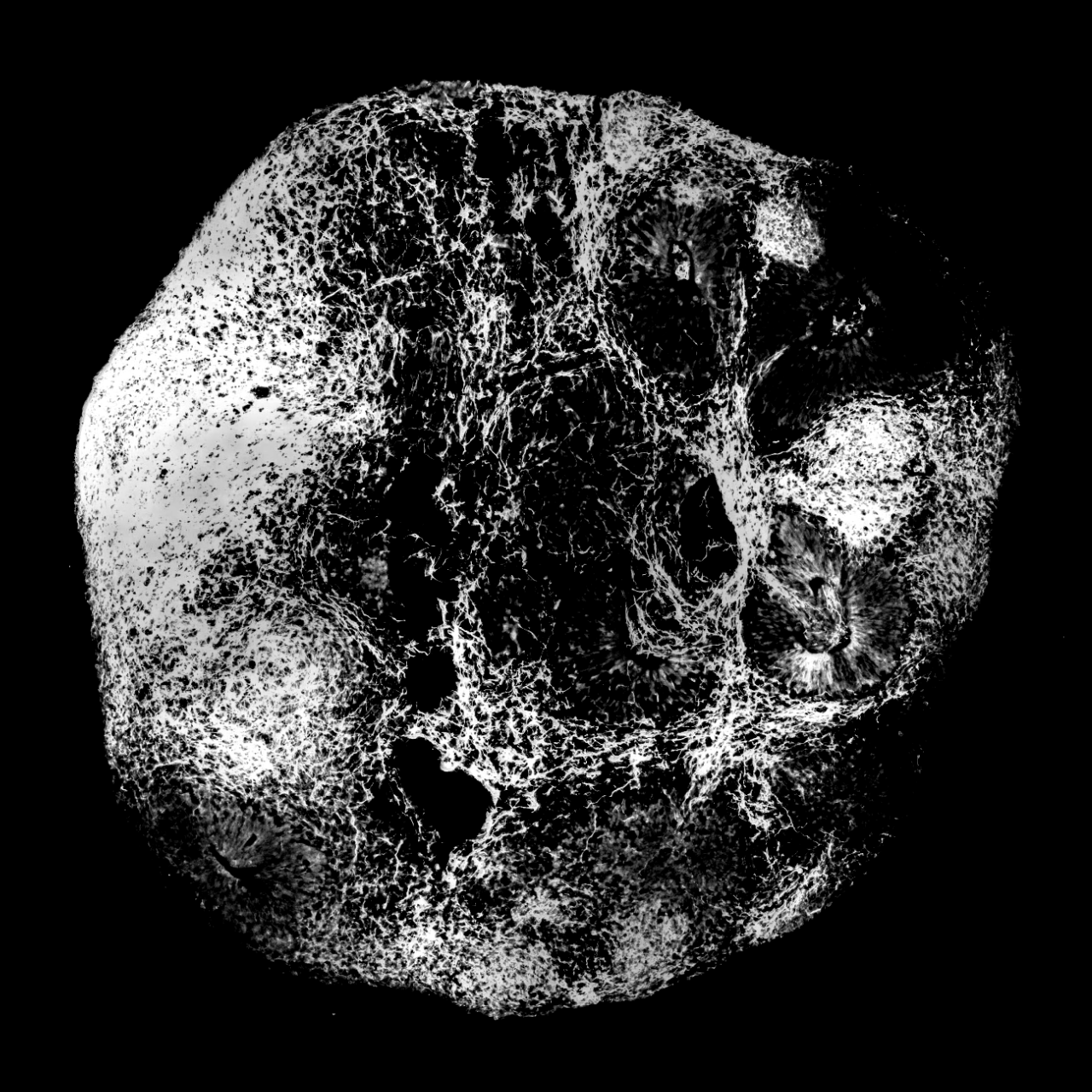

Supplement: Supplementary file 12 — Source data. [file 41556_2024_1412_MOESM12_ESM.zip › Lindenhoferetal-Fig-ED4-sourcedata-NCB/Lindenhoferetal-Fig-ED4-images-NCB/Lindenhoferetal-Fig-ED4c-day41-MAP2.tif]

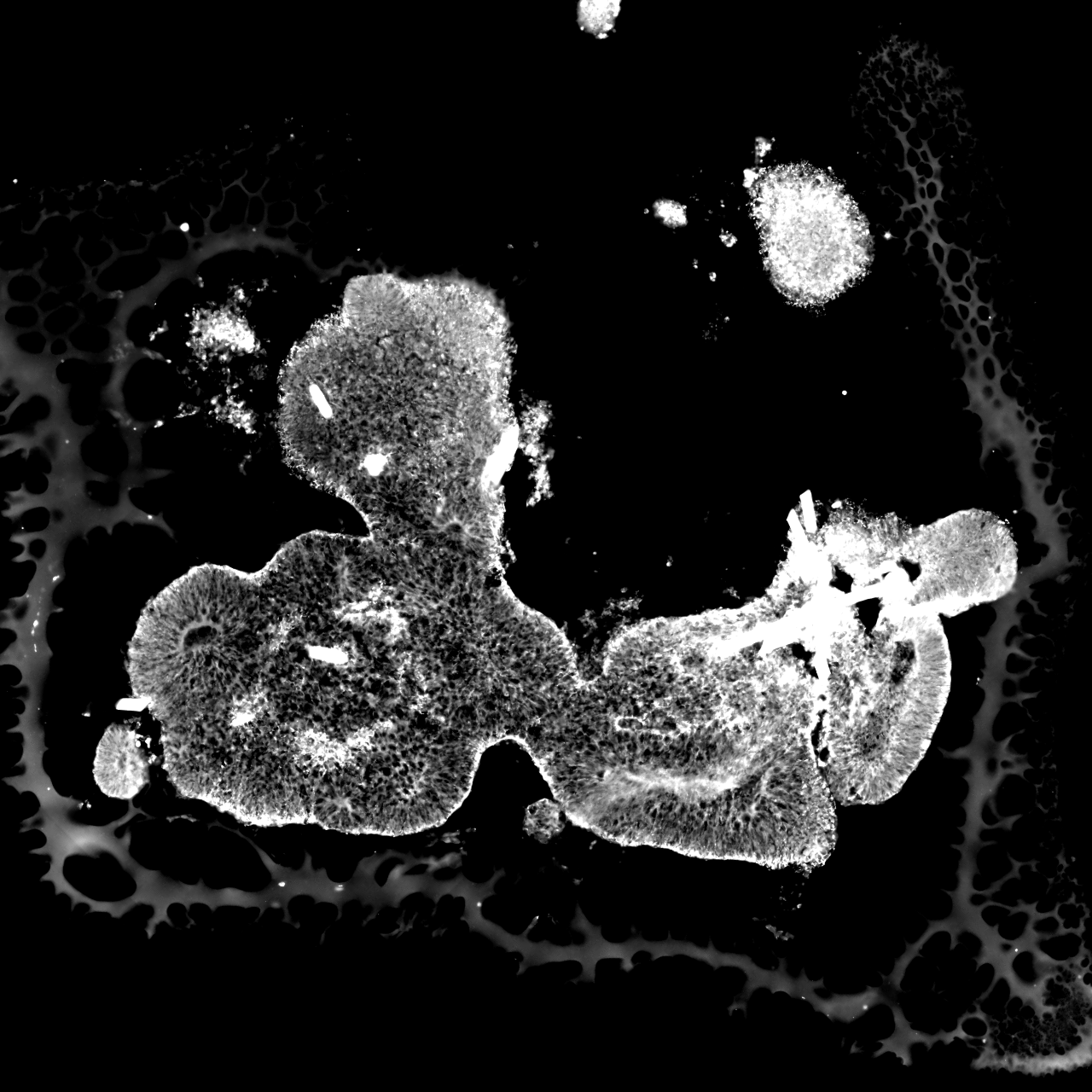

Supplement: Supplementary file 12 — Source data. [file 41556_2024_1412_MOESM12_ESM.zip › Lindenhoferetal-Fig-ED4-sourcedata-NCB/Lindenhoferetal-Fig-ED4-images-NCB/Lindenhoferetal-Fig-ED4c-day16-nestin.tif]

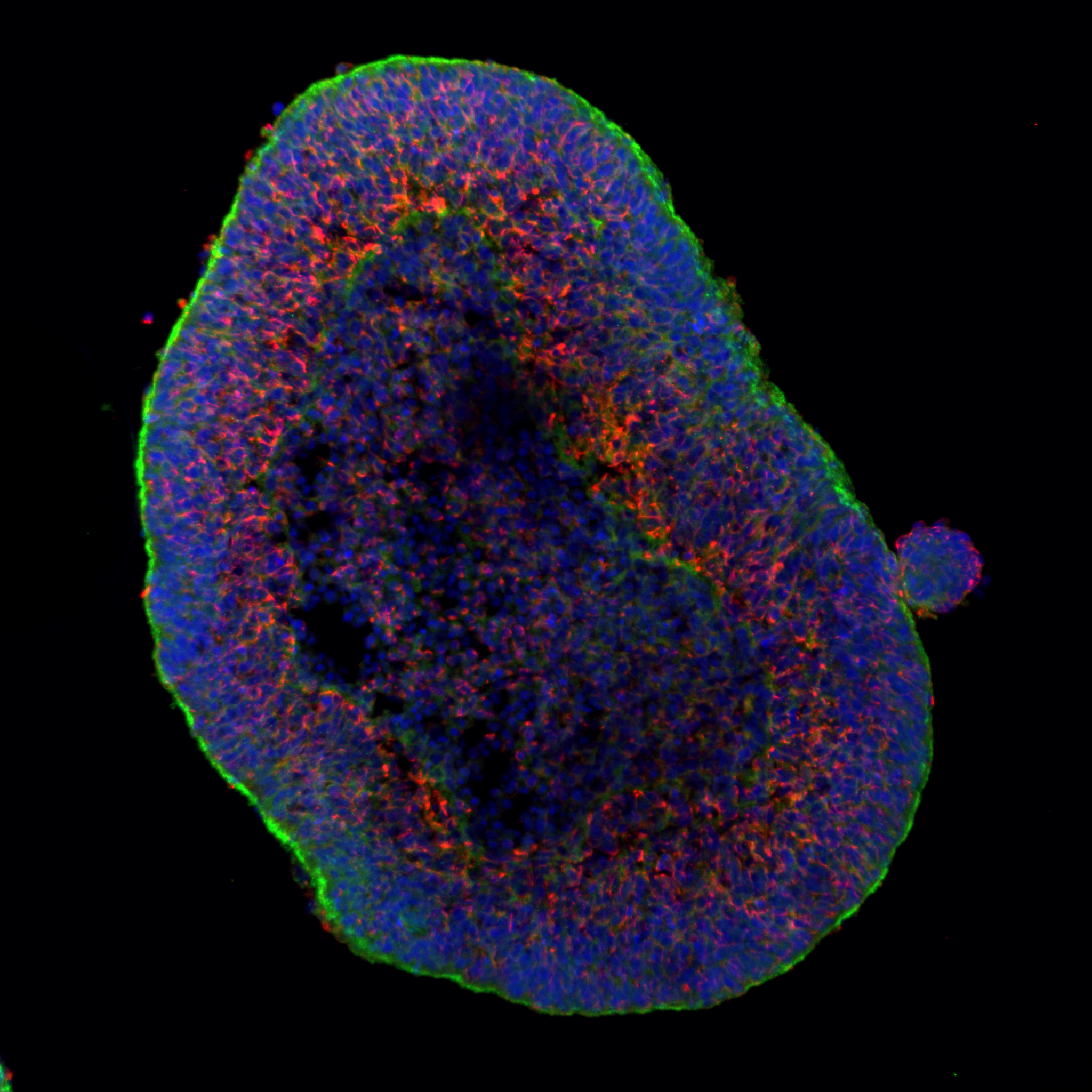

Supplement: Supplementary file 12 — Source data. [file 41556_2024_1412_MOESM12_ESM.zip › Lindenhoferetal-Fig-ED4-sourcedata-NCB/Lindenhoferetal-Fig-ED4-images-NCB/Lindenhoferetal-Fig-ED4a-day10-DAPI-aPKCzeta-nestin.tif]

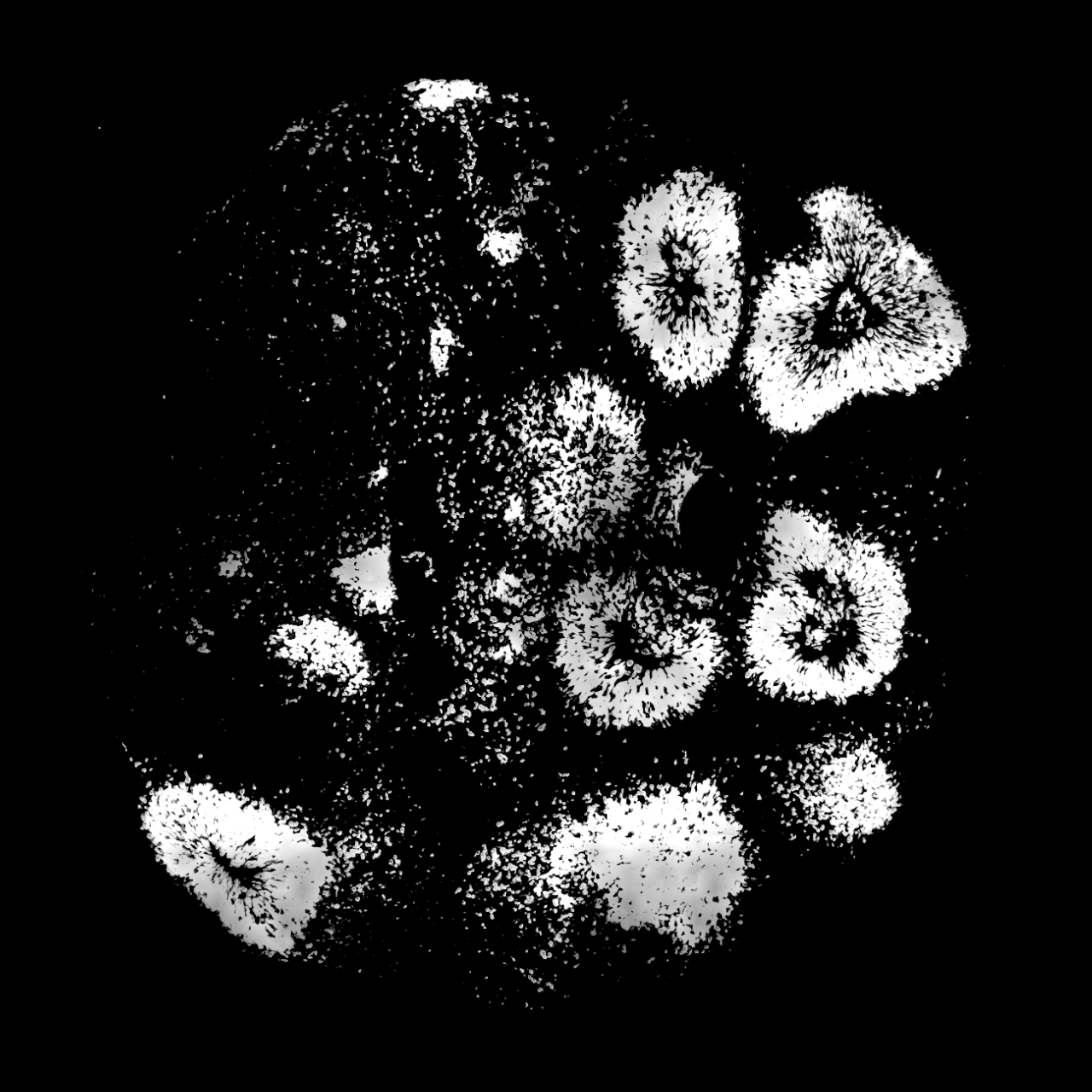

Supplement: Supplementary file 12 — Source data. [file 41556_2024_1412_MOESM12_ESM.zip › Lindenhoferetal-Fig-ED4-sourcedata-NCB/Lindenhoferetal-Fig-ED4-images-NCB/Lindenhoferetal-Fig-ED4c-day41-Sox2.tif]

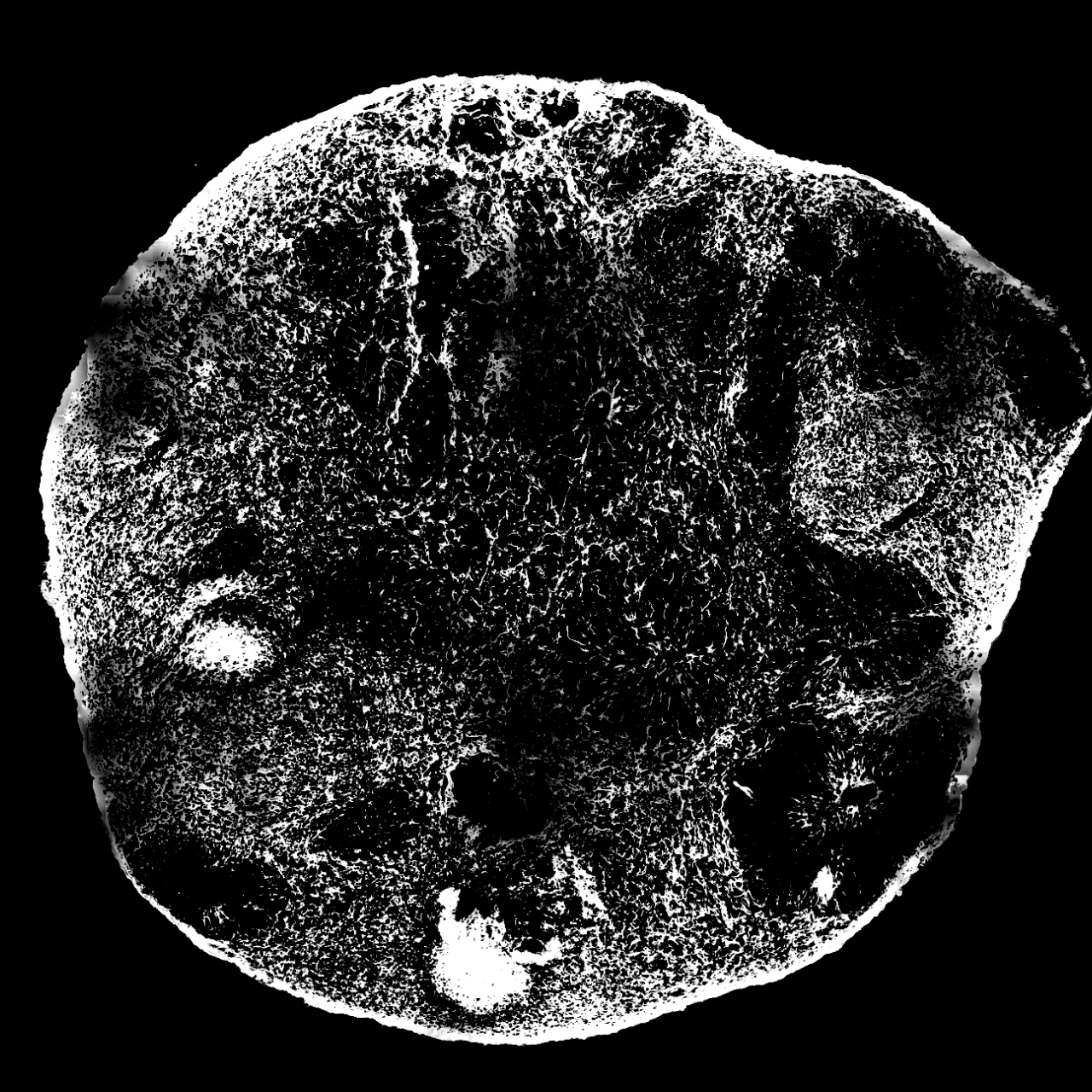

Supplement: Supplementary file 12 — Source data. [file 41556_2024_1412_MOESM12_ESM.zip › Lindenhoferetal-Fig-ED4-sourcedata-NCB/Lindenhoferetal-Fig-ED4-images-NCB/Lindenhoferetal-Fig-ED4c-day41-TuJI.tif]

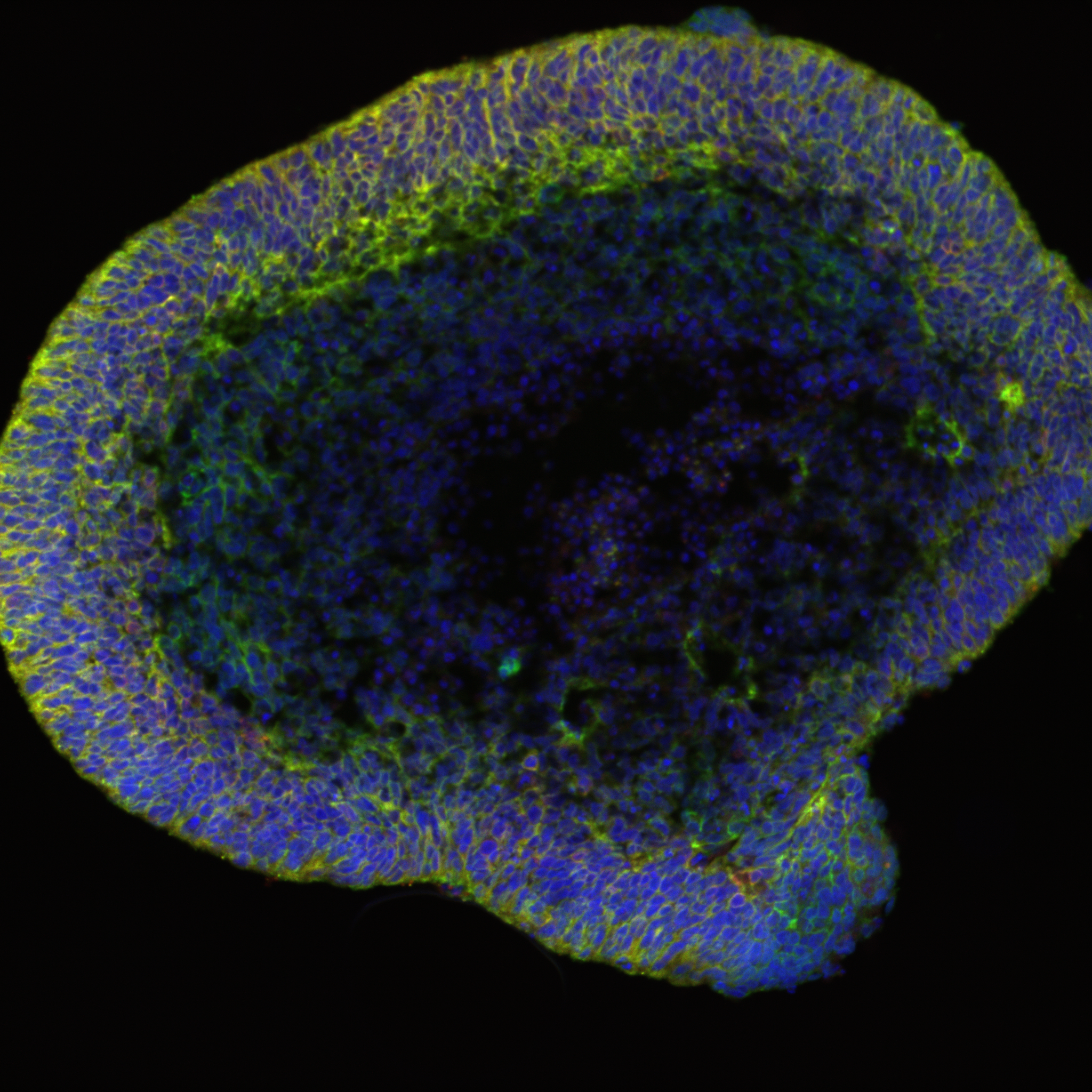

Supplement: Supplementary file 12 — Source data. [file 41556_2024_1412_MOESM12_ESM.zip › Lindenhoferetal-Fig-ED4-sourcedata-NCB/Lindenhoferetal-Fig-ED4-images-NCB/Lindenhoferetal-Fig-ED4b-day10-DAPI-betacatenin-ncadherin.tif]

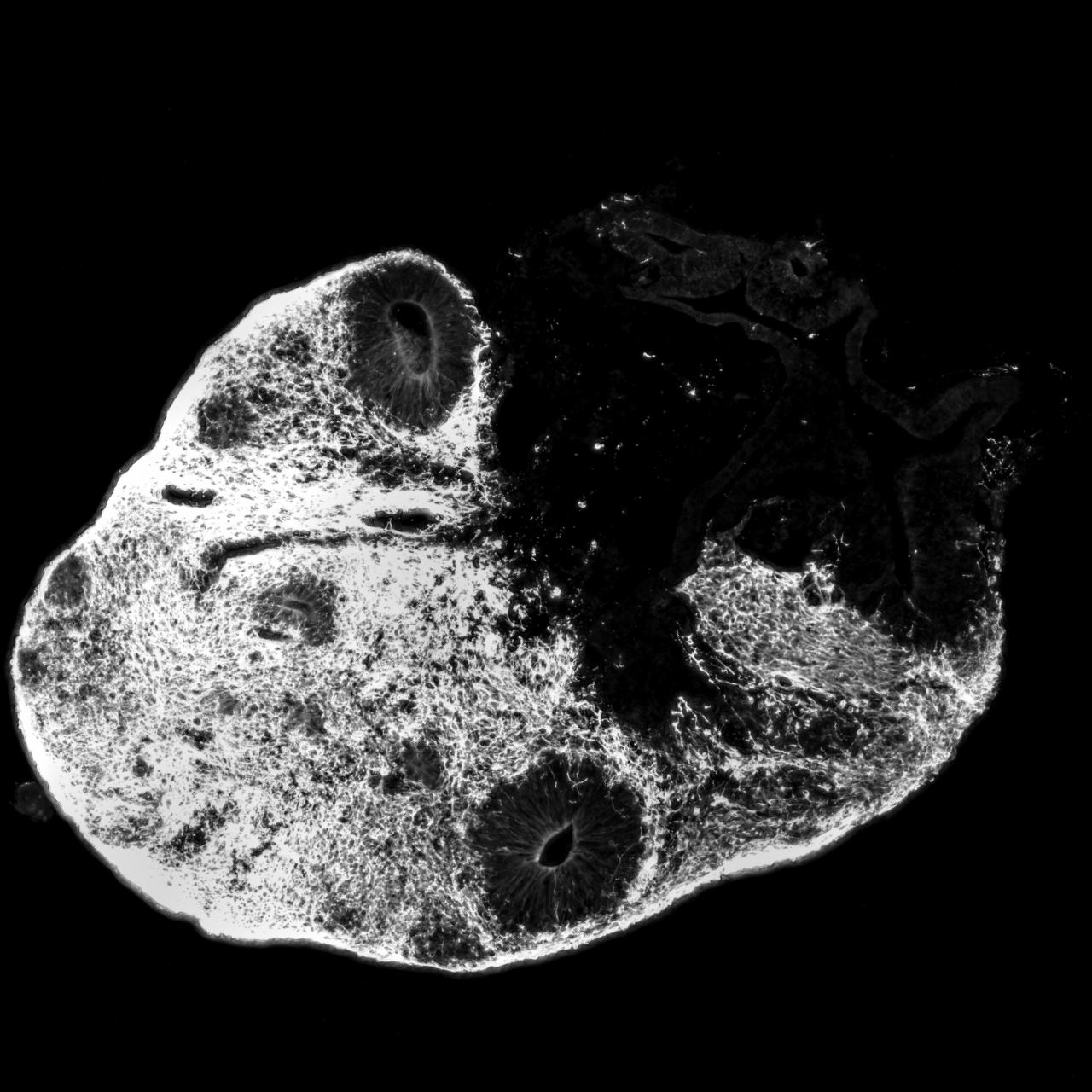

Supplement: Supplementary file 12 — Source data. [file 41556_2024_1412_MOESM12_ESM.zip › Lindenhoferetal-Fig-ED4-sourcedata-NCB/Lindenhoferetal-Fig-ED4-images-NCB/Lindenhoferetal-Fig-ED4c-day33-DCX.tif]

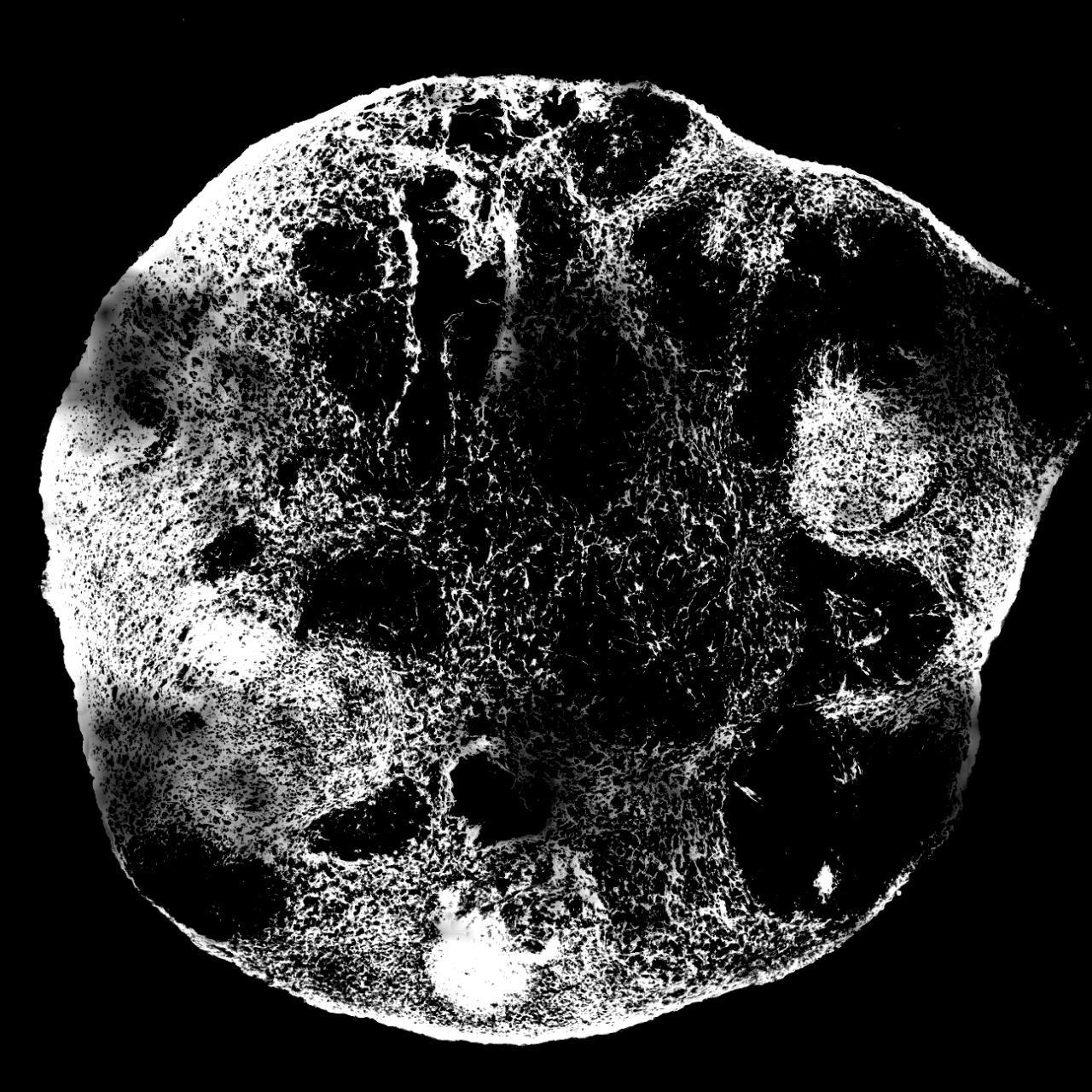

Supplement: Supplementary file 12 — Source data. [file 41556_2024_1412_MOESM12_ESM.zip › Lindenhoferetal-Fig-ED4-sourcedata-NCB/Lindenhoferetal-Fig-ED4-images-NCB/Lindenhoferetal-Fig-ED4c-day41-DCX.tif]

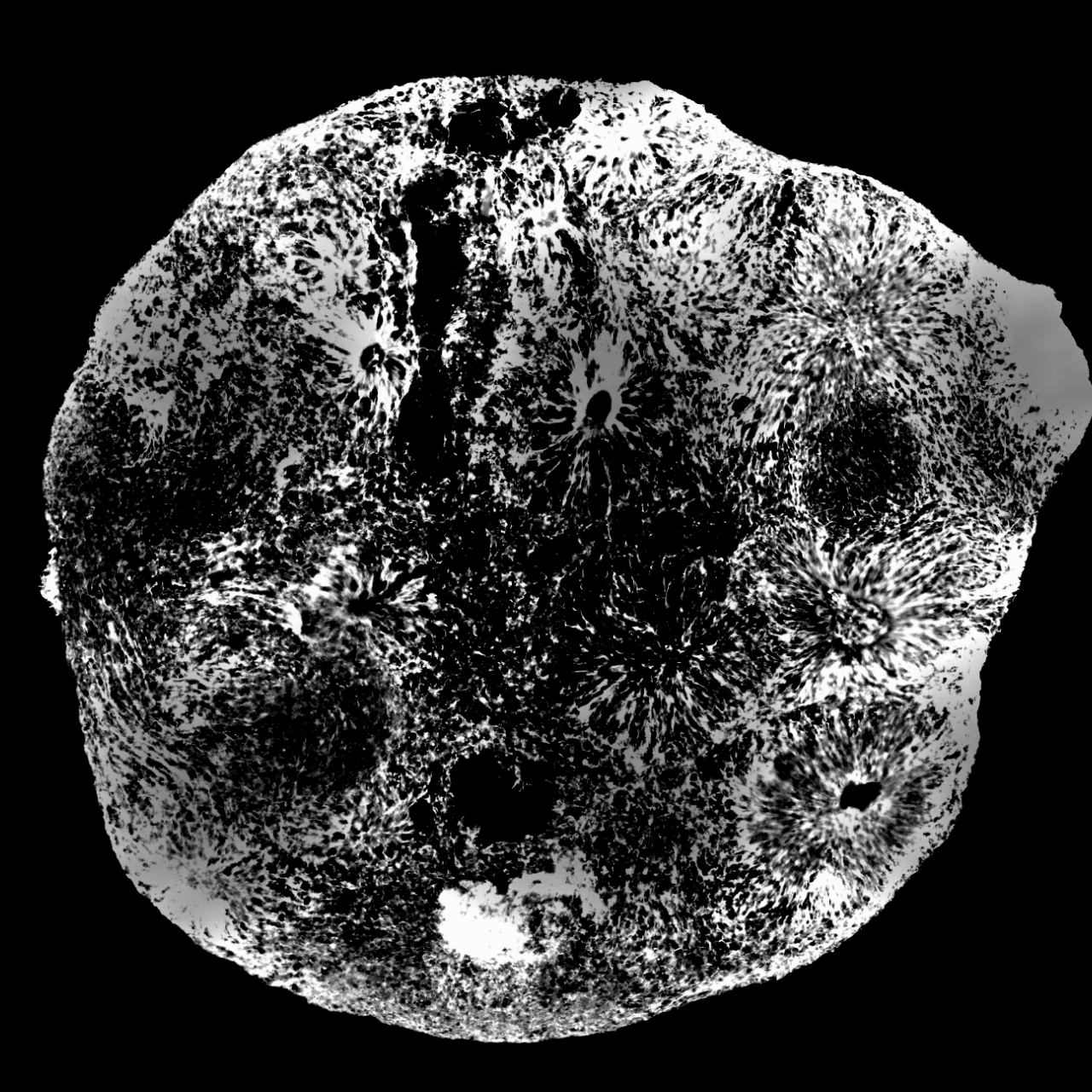

Supplement: Supplementary file 12 — Source data. [file 41556_2024_1412_MOESM12_ESM.zip › Lindenhoferetal-Fig-ED4-sourcedata-NCB/Lindenhoferetal-Fig-ED4-images-NCB/Lindenhoferetal-Fig-ED4c-day41-nestin.tif]

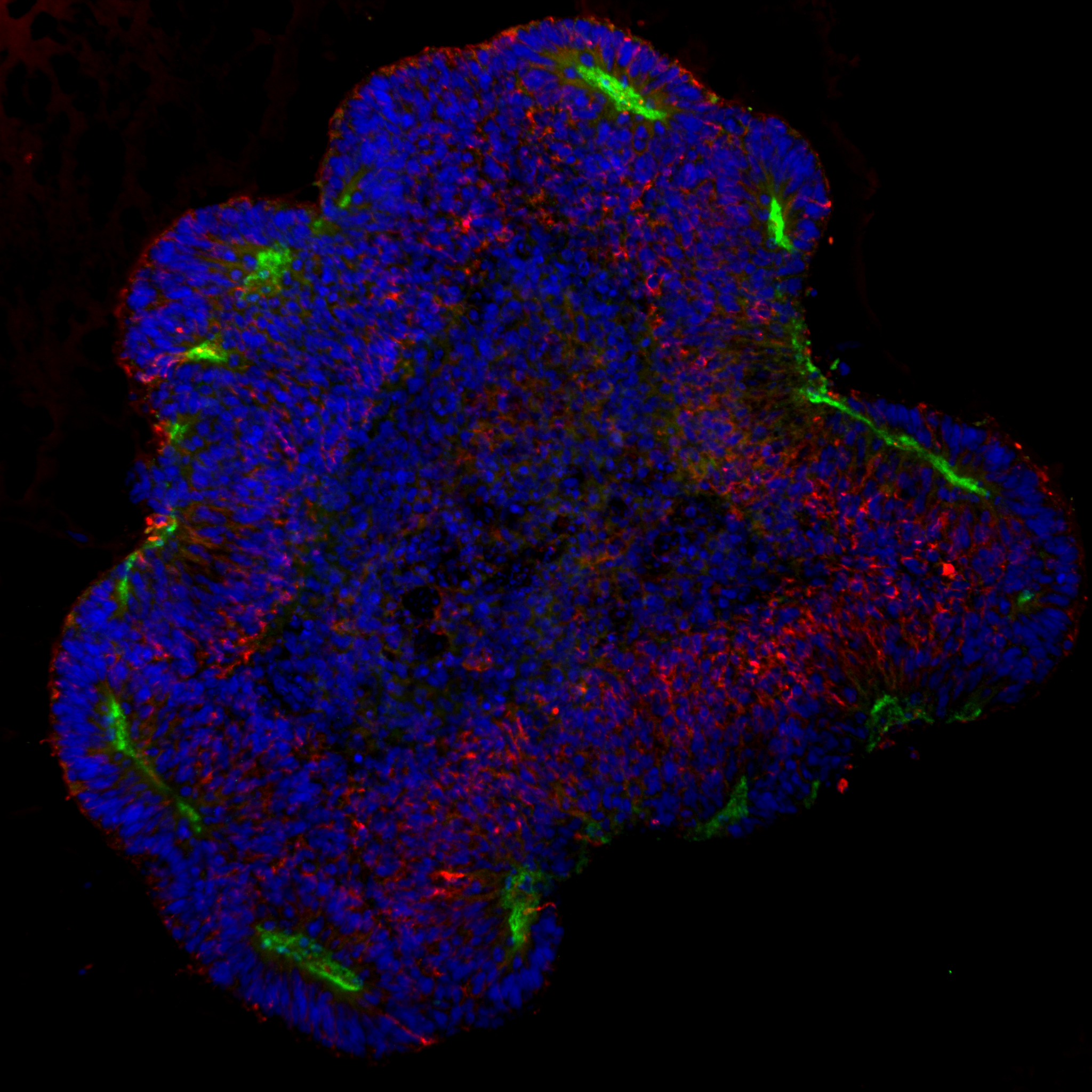

Supplement: Supplementary file 12 — Source data. [file 41556_2024_1412_MOESM12_ESM.zip › Lindenhoferetal-Fig-ED4-sourcedata-NCB/Lindenhoferetal-Fig-ED4-images-NCB/Lindenhoferetal-Fig-ED4a-day11-DAPI-aPKCzeta-nestin.tif]

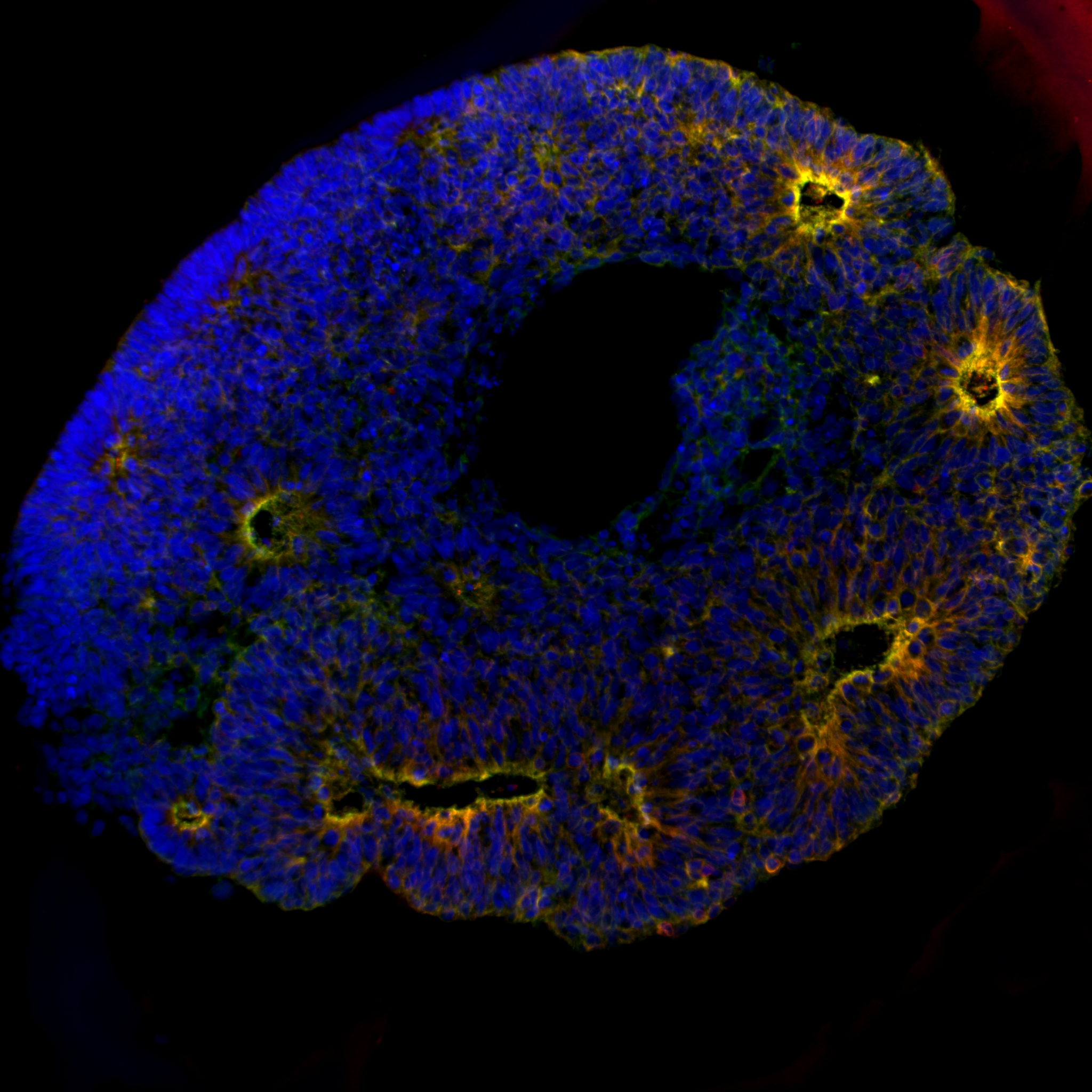

Supplement: Supplementary file 12 — Source data. [file 41556_2024_1412_MOESM12_ESM.zip › Lindenhoferetal-Fig-ED4-sourcedata-NCB/Lindenhoferetal-Fig-ED4-images-NCB/Lindenhoferetal-Fig-ED4b-day11-DAPI-betacatenin-ncadherin.tif]

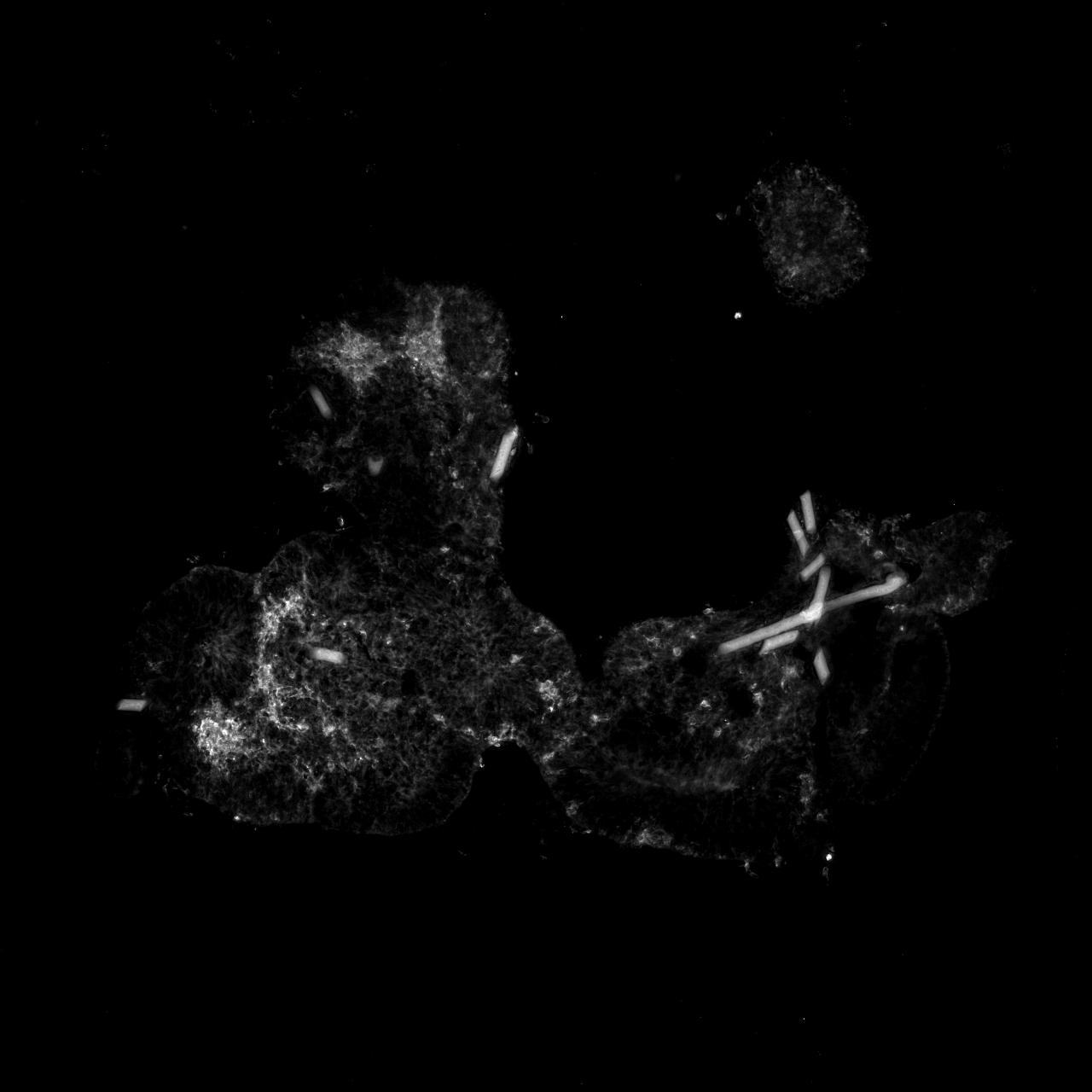

Supplement: Supplementary file 12 — Source data. [file 41556_2024_1412_MOESM12_ESM.zip › Lindenhoferetal-Fig-ED4-sourcedata-NCB/Lindenhoferetal-Fig-ED4-images-NCB/Lindenhoferetal-Fig-ED4c-day16-DCX.tif]

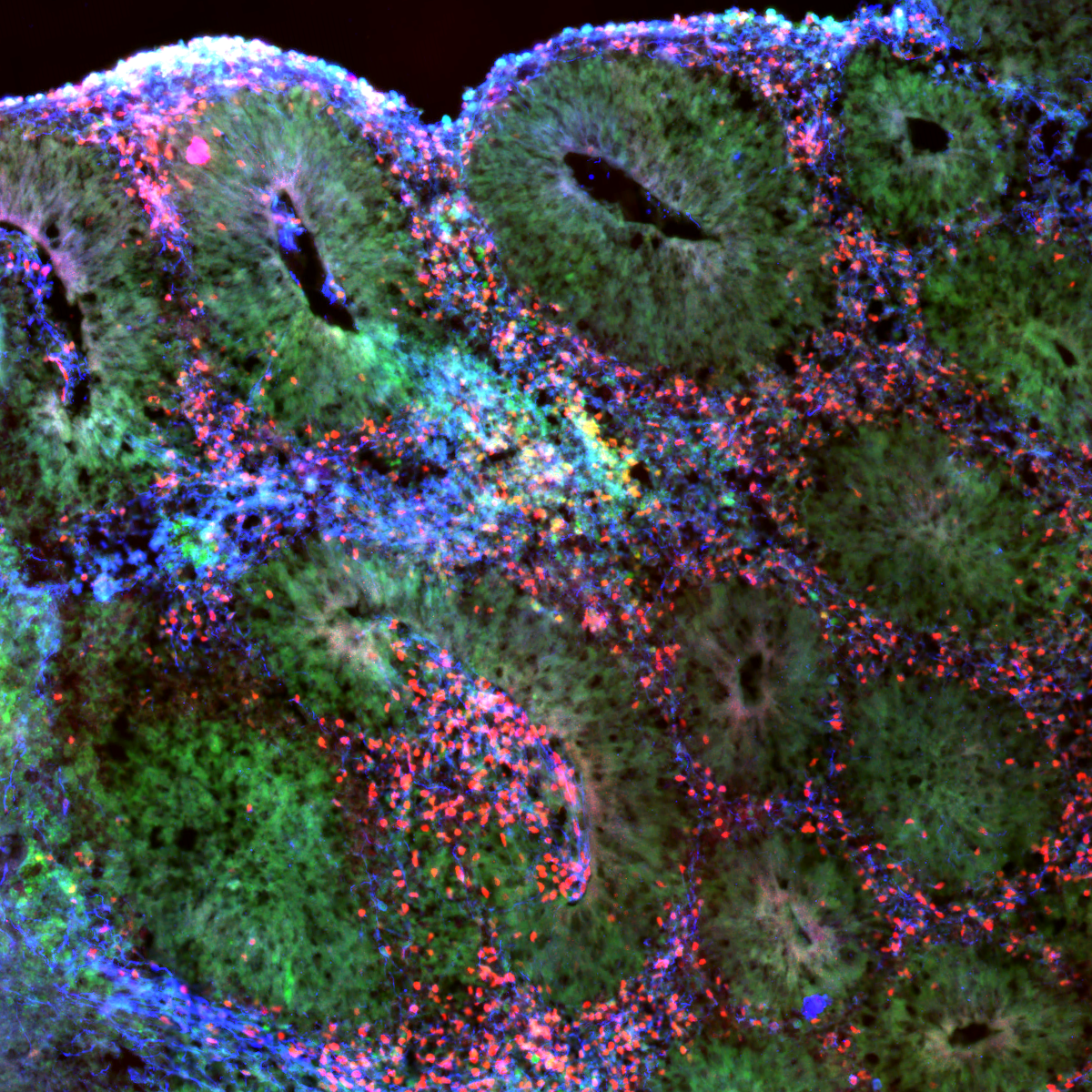

Supplement: Supplementary file 15 — Source data. [file 41556_2024_1412_MOESM15_ESM.zip › Lindenhoferetal-Fig-ED7-sourcedata-NCB/Lindenhoferetal-Fig-ED7-images-NCB/Lindenhoferetal-Fig-ED7-d.tif]

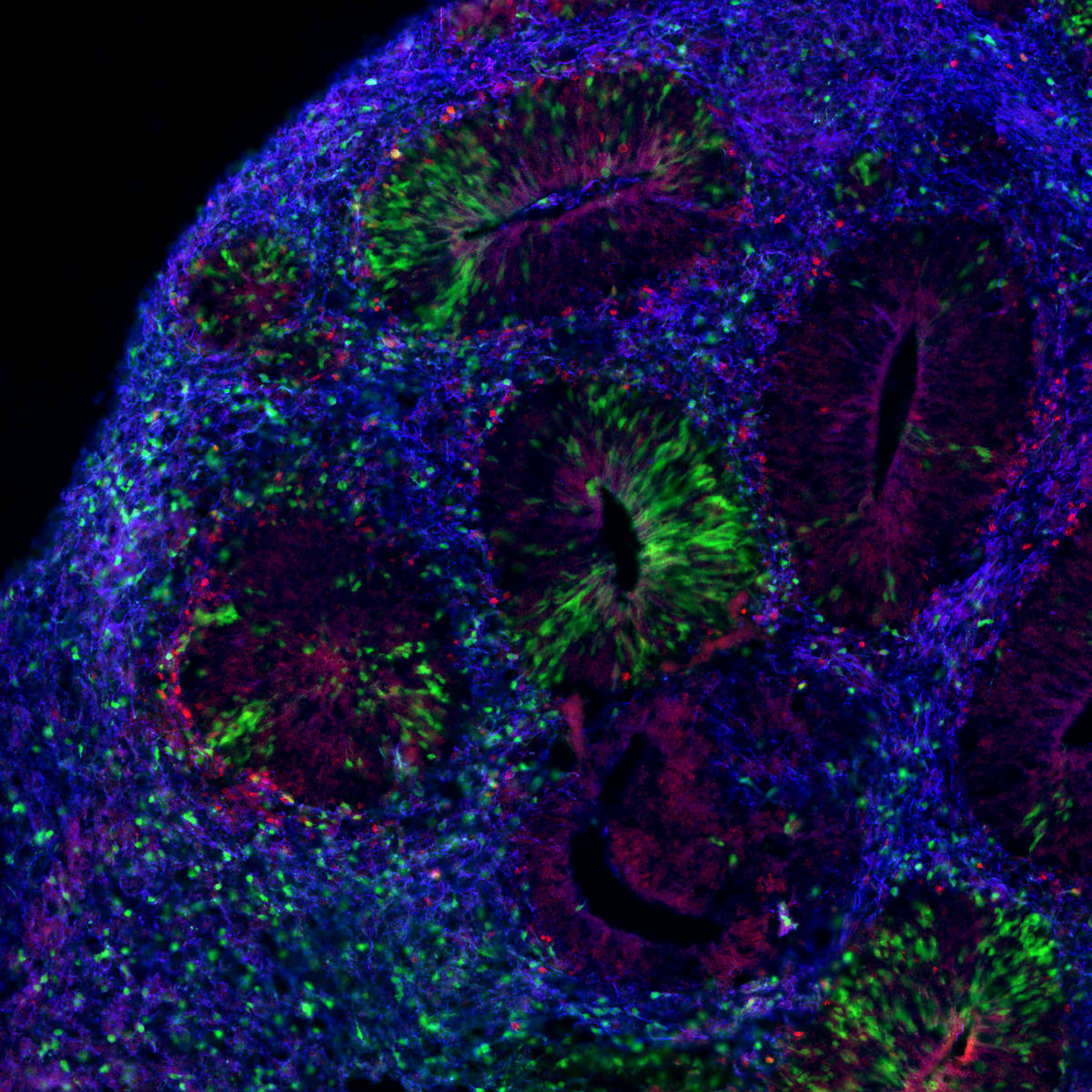

Supplement: Supplementary file 15 — Source data. [file 41556_2024_1412_MOESM15_ESM.zip › Lindenhoferetal-Fig-ED7-sourcedata-NCB/Lindenhoferetal-Fig-ED7-images-NCB/Lindenhoferetal-Fig-ED7-e.tif]

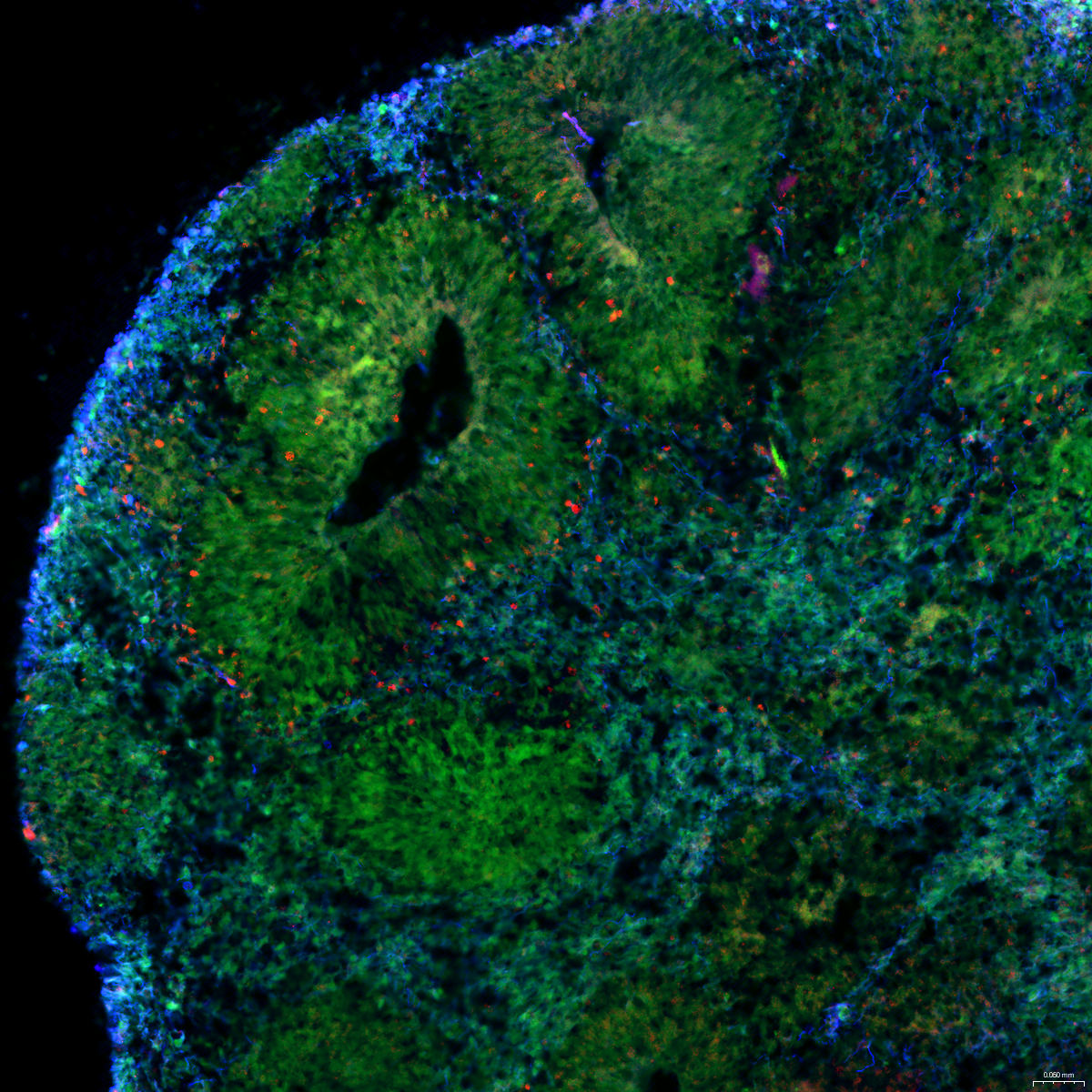

Supplement: Supplementary file 15 — Source data. [file 41556_2024_1412_MOESM15_ESM.zip › Lindenhoferetal-Fig-ED7-sourcedata-NCB/Lindenhoferetal-Fig-ED7-images-NCB/Lindenhoferetal-Fig-ED7-f.tif]

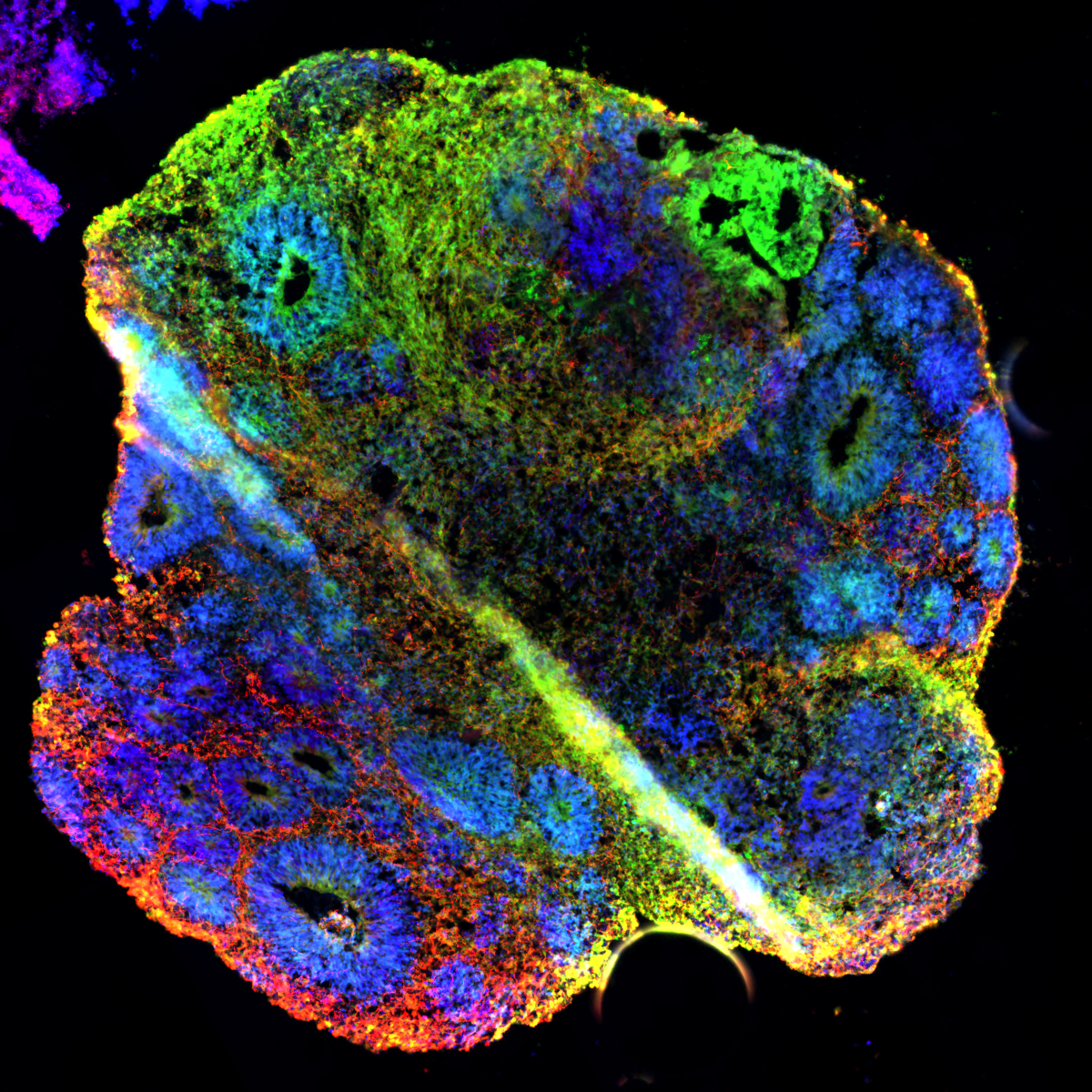

Supplement: Supplementary file 15 — Source data. [file 41556_2024_1412_MOESM15_ESM.zip › Lindenhoferetal-Fig-ED7-sourcedata-NCB/Lindenhoferetal-Fig-ED7-images-NCB/Lindenhoferetal-Fig-ED7-b.tif]

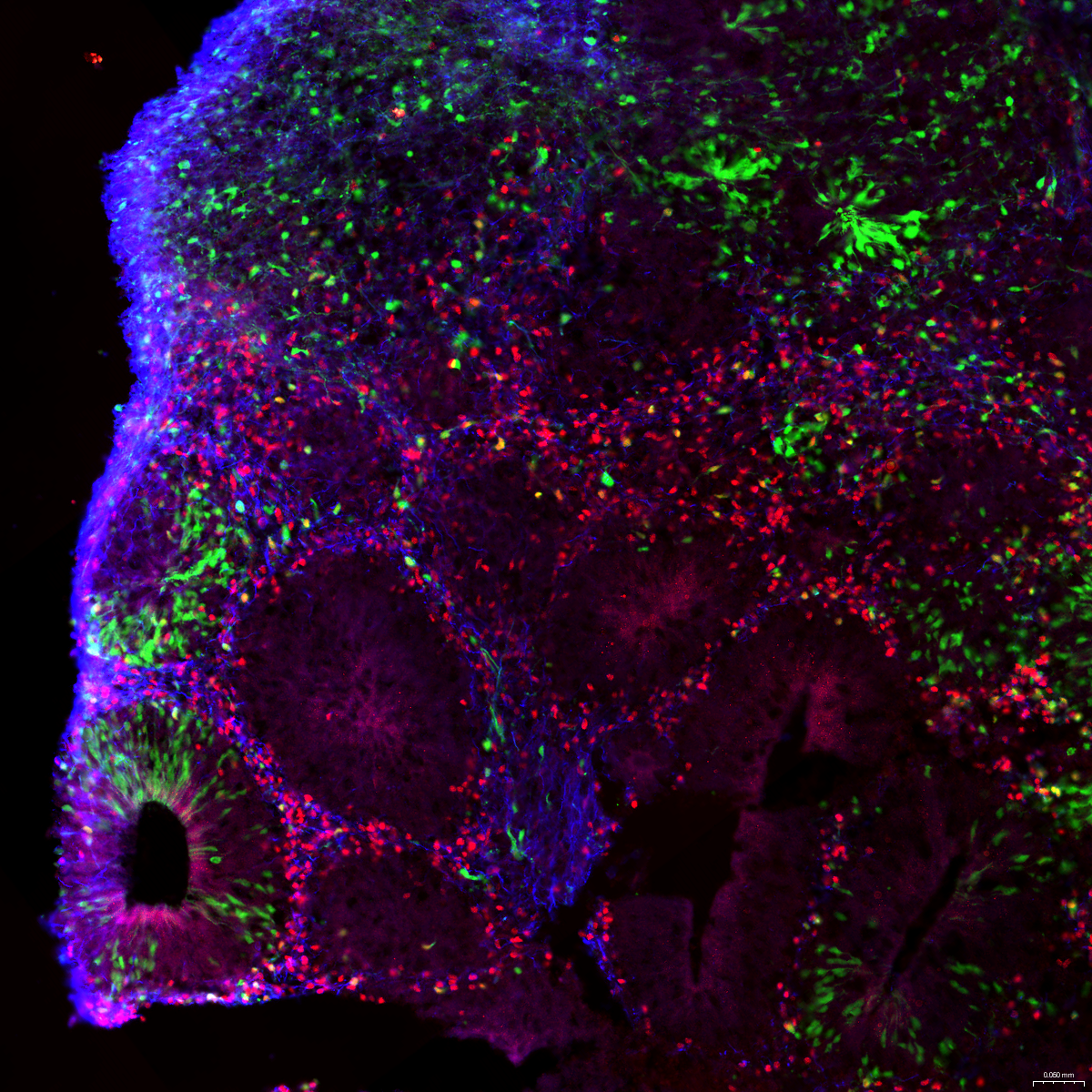

Supplement: Supplementary file 15 — Source data. [file 41556_2024_1412_MOESM15_ESM.zip › Lindenhoferetal-Fig-ED7-sourcedata-NCB/Lindenhoferetal-Fig-ED7-images-NCB/Lindenhoferetal-Fig-ED7-c.tif]

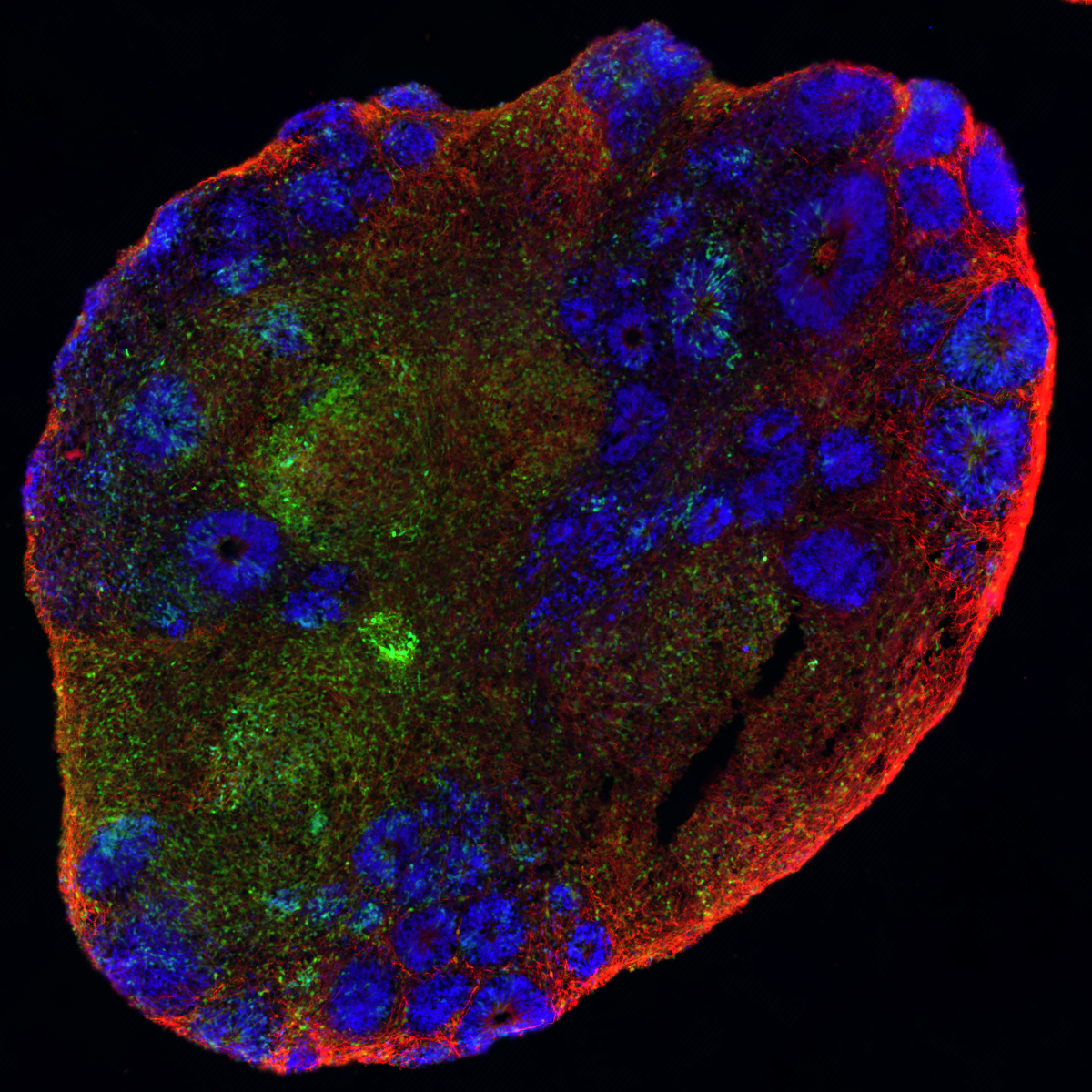

Supplement: Supplementary file 15 — Source data. [file 41556_2024_1412_MOESM15_ESM.zip › Lindenhoferetal-Fig-ED7-sourcedata-NCB/Lindenhoferetal-Fig-ED7-images-NCB/Lindenhoferetal-Fig-ED7-a.tif]

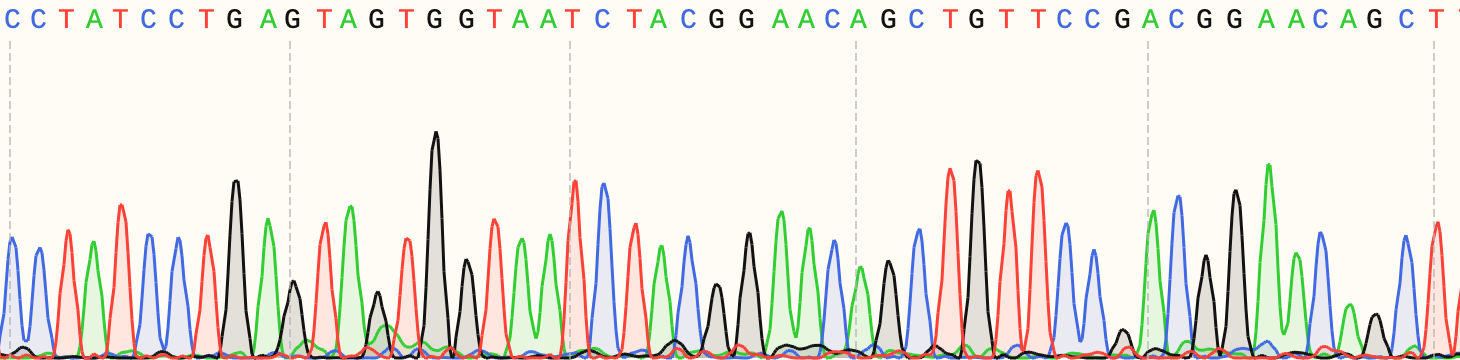

Supplement: Supplementary file 16 — Source data. [file 41556_2024_1412_MOESM16_ESM.zip › Lindenhoferetal-Fig-ED8-sourcedata-NCB/Lindenhoferetal-Fig-ED8-Sanger-and-TIDE/Lindenhoferetal-Fig-ED8-c-Sanger-Seq-TP53-locus-KO.png]

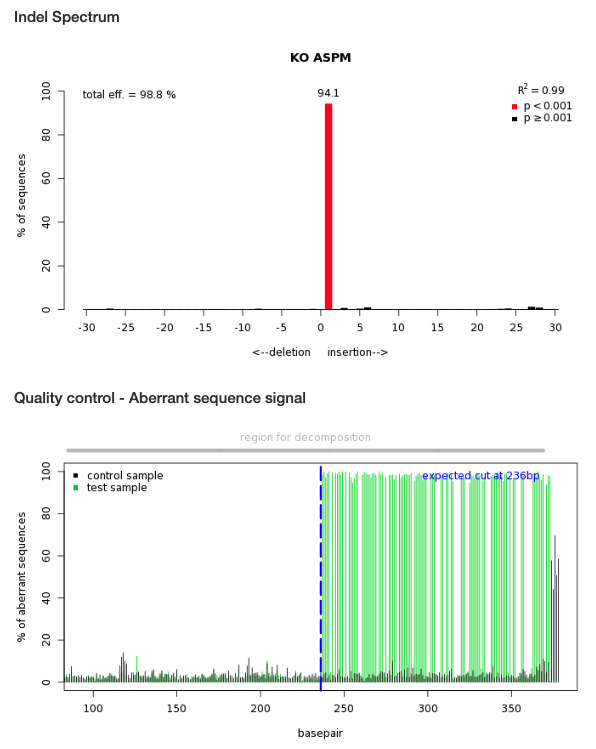

Supplement: Supplementary file 16 — Source data. [file 41556_2024_1412_MOESM16_ESM.zip › Lindenhoferetal-Fig-ED8-sourcedata-NCB/Lindenhoferetal-Fig-ED8-Sanger-and-TIDE/Lindenhoferetal-Fig-ED8-a-KO-ASPM-TIDE.png]

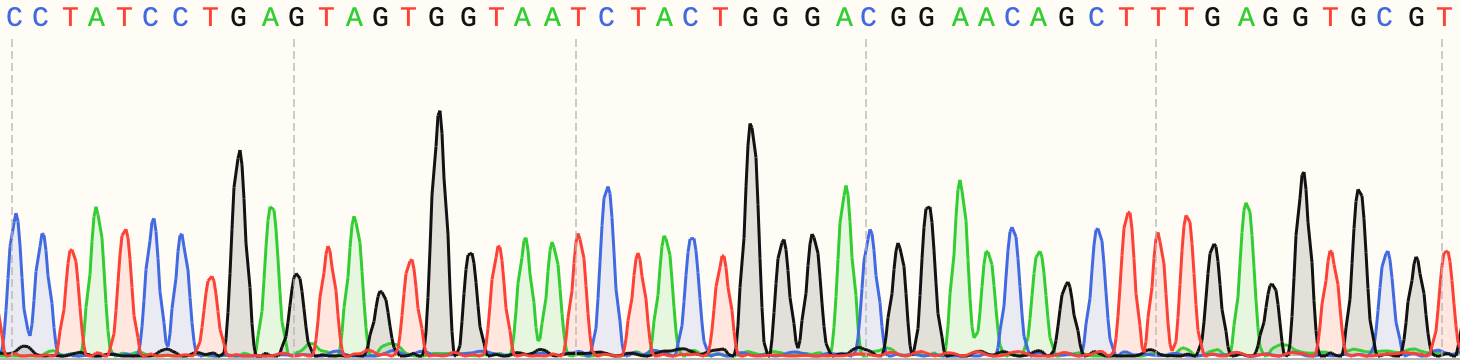

Supplement: Supplementary file 16 — Source data. [file 41556_2024_1412_MOESM16_ESM.zip › Lindenhoferetal-Fig-ED8-sourcedata-NCB/Lindenhoferetal-Fig-ED8-Sanger-and-TIDE/Lindenhoferetal-Fig-ED8-c-Sanger-Seq-TP53-locus-WT.png]

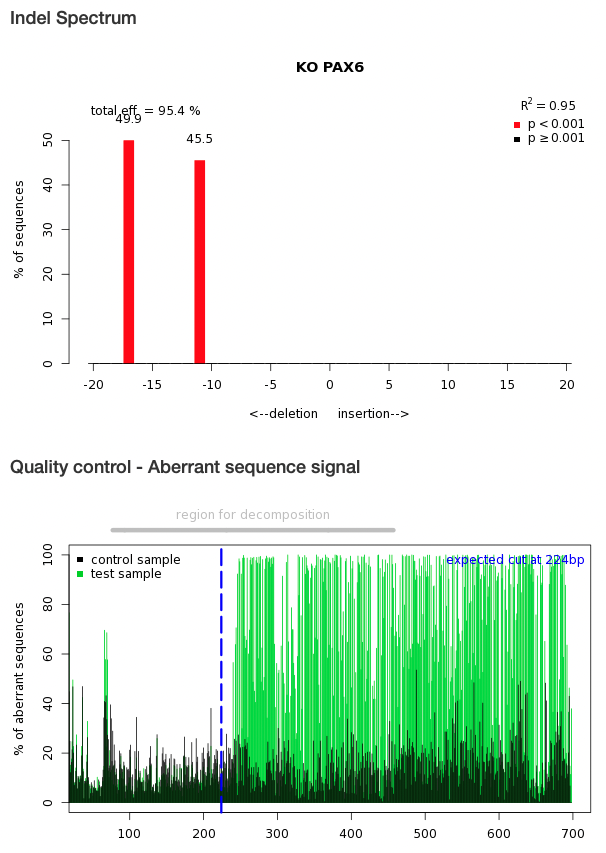

Supplement: Supplementary file 16 — Source data. [file 41556_2024_1412_MOESM16_ESM.zip › Lindenhoferetal-Fig-ED8-sourcedata-NCB/Lindenhoferetal-Fig-ED8-Sanger-and-TIDE/Lindenhoferetal-Fig-ED8-e-KO-PAX6-TIDE.png]

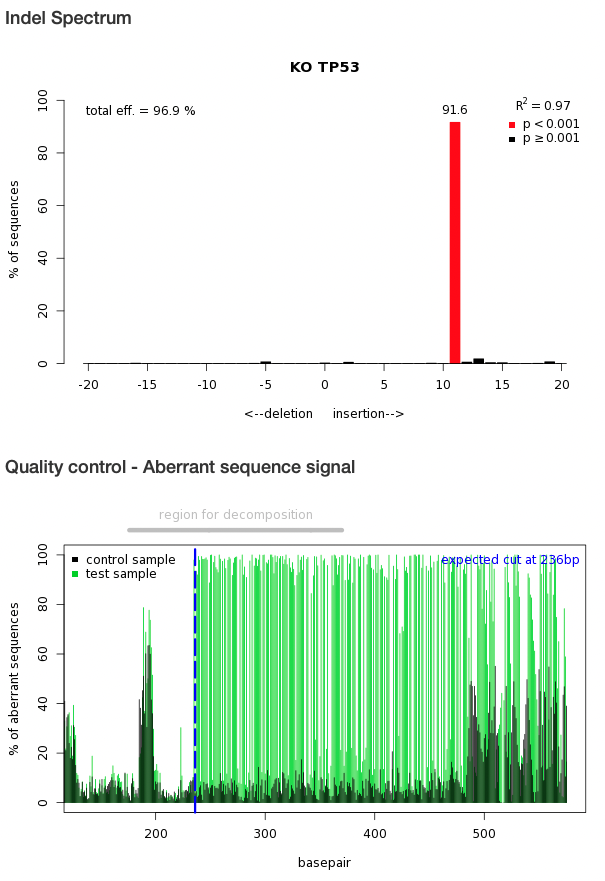

Supplement: Supplementary file 16 — Source data. [file 41556_2024_1412_MOESM16_ESM.zip › Lindenhoferetal-Fig-ED8-sourcedata-NCB/Lindenhoferetal-Fig-ED8-Sanger-and-TIDE/Lindenhoferetal-Fig-ED8-c-KO-TP53-TIDE.png]

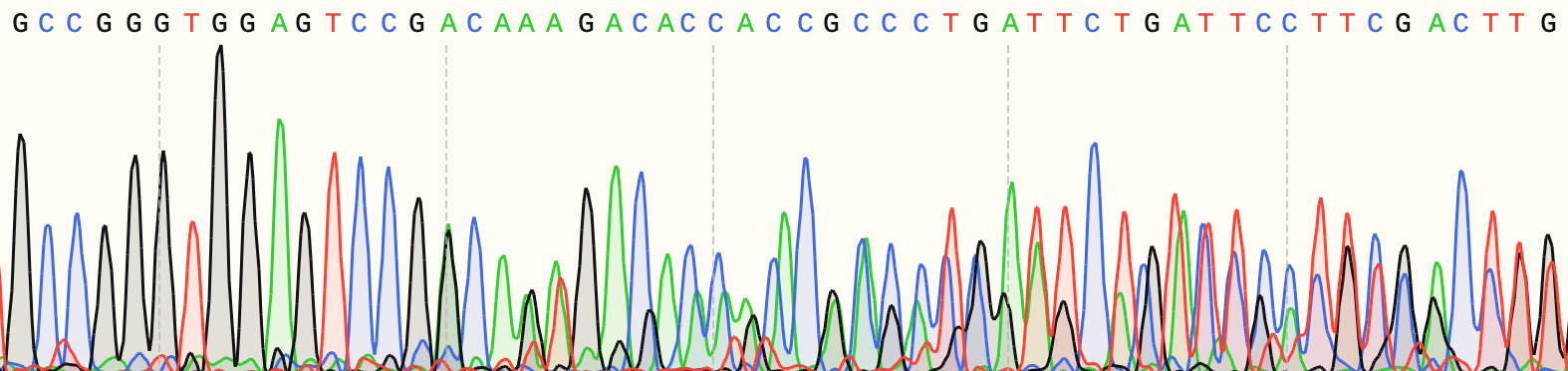

Supplement: Supplementary file 16 — Source data. [file 41556_2024_1412_MOESM16_ESM.zip › Lindenhoferetal-Fig-ED8-sourcedata-NCB/Lindenhoferetal-Fig-ED8-Sanger-and-TIDE/Lindenhoferetal-Fig-ED8-e-Sanger-Seq-PAX6-locus-KO.png]

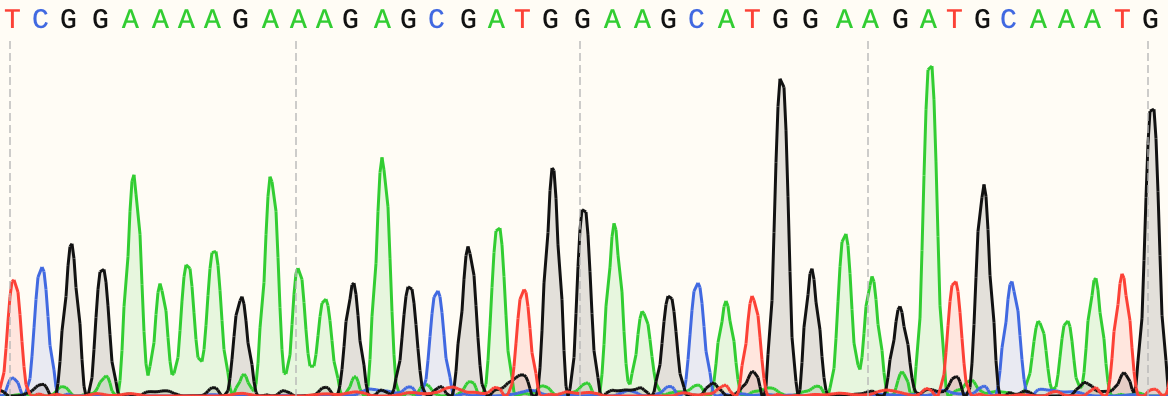

Supplement: Supplementary file 16 — Source data. [file 41556_2024_1412_MOESM16_ESM.zip › Lindenhoferetal-Fig-ED8-sourcedata-NCB/Lindenhoferetal-Fig-ED8-Sanger-and-TIDE/Lindenhoferetal-Fig-ED8-a-Sanger-Seq-ASPM-locus-WT.png]

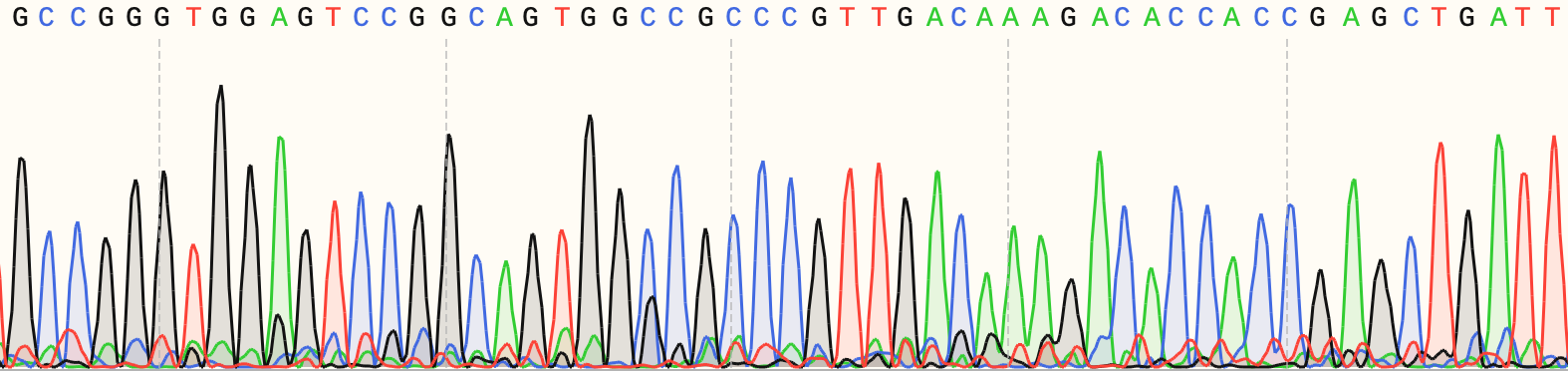

Supplement: Supplementary file 16 — Source data. [file 41556_2024_1412_MOESM16_ESM.zip › Lindenhoferetal-Fig-ED8-sourcedata-NCB/Lindenhoferetal-Fig-ED8-Sanger-and-TIDE/Lindenhoferetal-Fig-ED8-e-Sanger-Seq-PAX6-locus-WT.png]

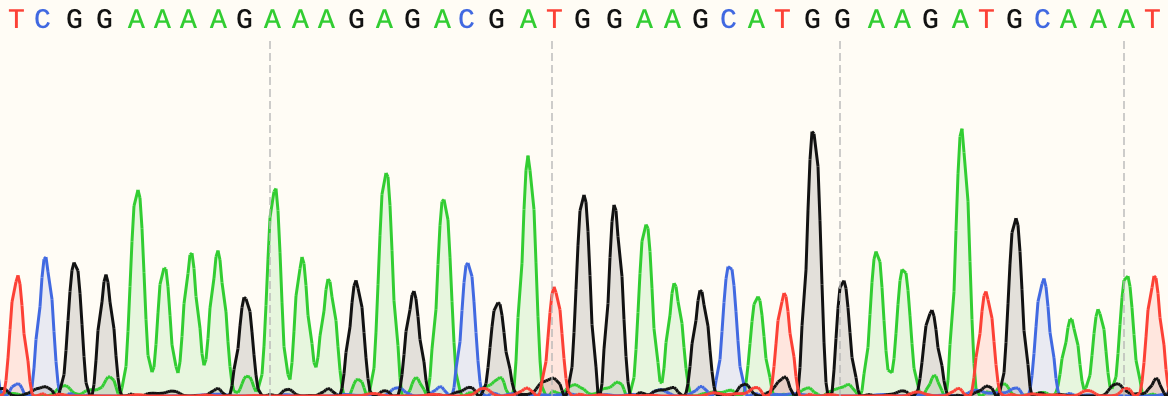

Supplement: Supplementary file 16 — Source data. [file 41556_2024_1412_MOESM16_ESM.zip › Lindenhoferetal-Fig-ED8-sourcedata-NCB/Lindenhoferetal-Fig-ED8-Sanger-and-TIDE/Lindenhoferetal-Fig-ED8-a-Sanger-Seq-ASPM-locus-KO.png]

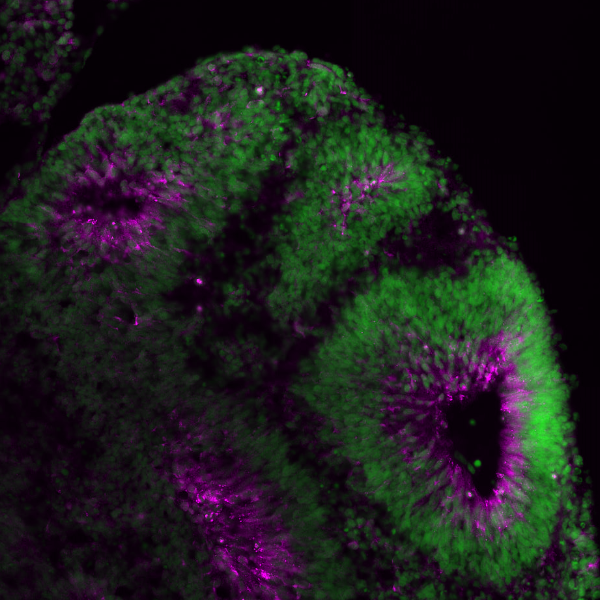

Supplement: Supplementary file 16 — Source data. [file 41556_2024_1412_MOESM16_ESM.zip › Lindenhoferetal-Fig-ED8-sourcedata-NCB/Lindenhoferetal-Fig-ED8-images-NCB/Lindenhoferetal-Fig-ED8-b-ASPM-WT-DAPI-ASPM.tif]

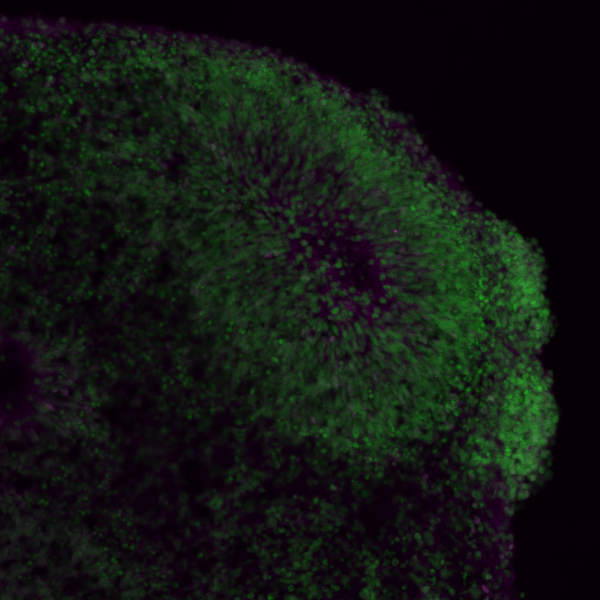

Supplement: Supplementary file 16 — Source data. [file 41556_2024_1412_MOESM16_ESM.zip › Lindenhoferetal-Fig-ED8-sourcedata-NCB/Lindenhoferetal-Fig-ED8-images-NCB/Lindenhoferetal-Fig-ED8-b-ASPM-KO-DAPI-ASPM.tif]

# Unprocessed images in Extended Data Figure 8

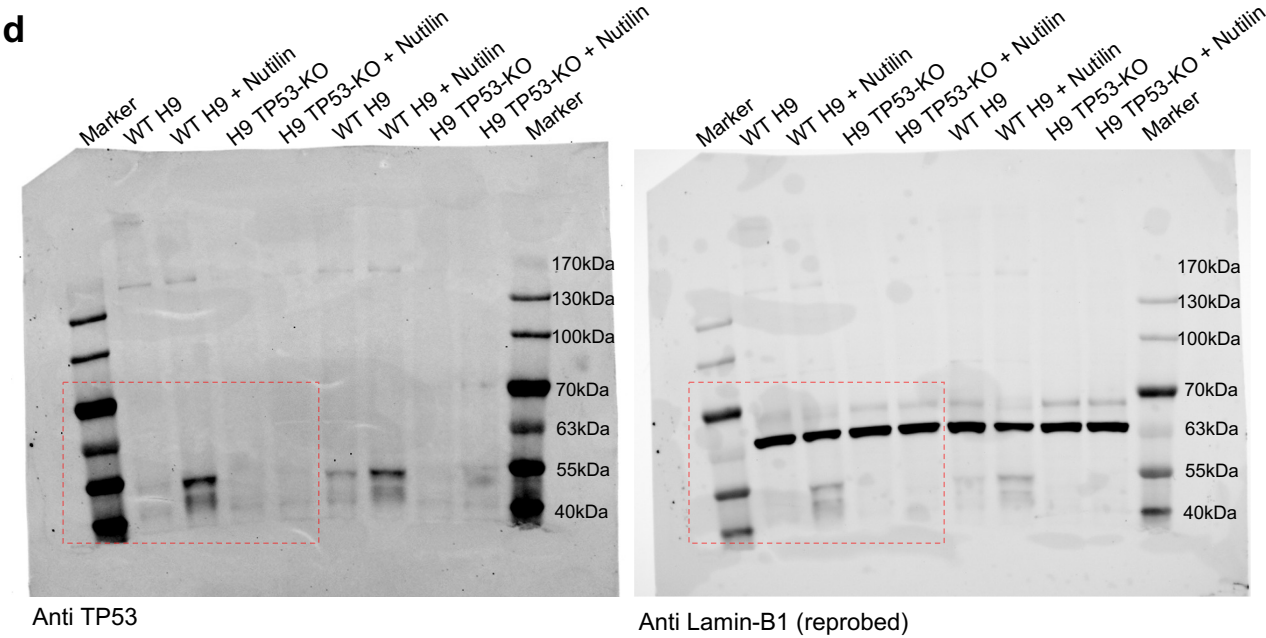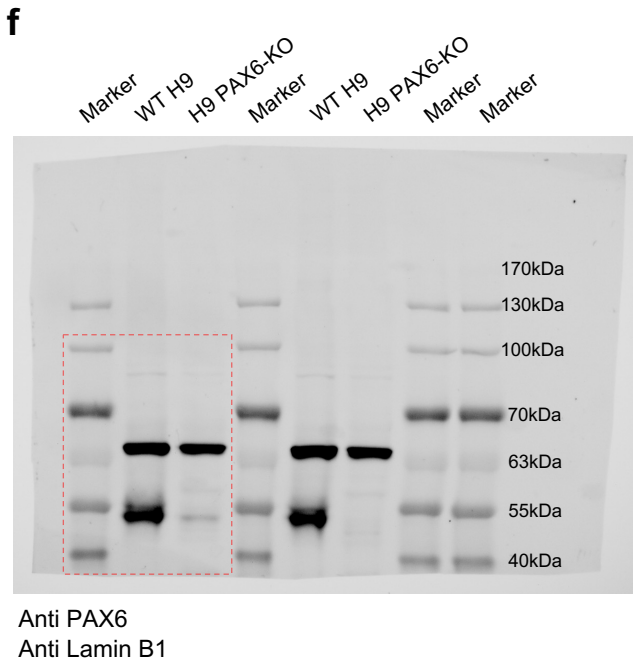

Supplement: Supplementary file 16 — Source data. [file 41556_2024_1412_MOESM16_ESM.zip › Lindenhoferetal-Fig-ED8-sourcedata-NCB/Lindenhoferetal-Fig-ED8-westernblots/240222-sourcedata-uncropped-westernblots.pdf]

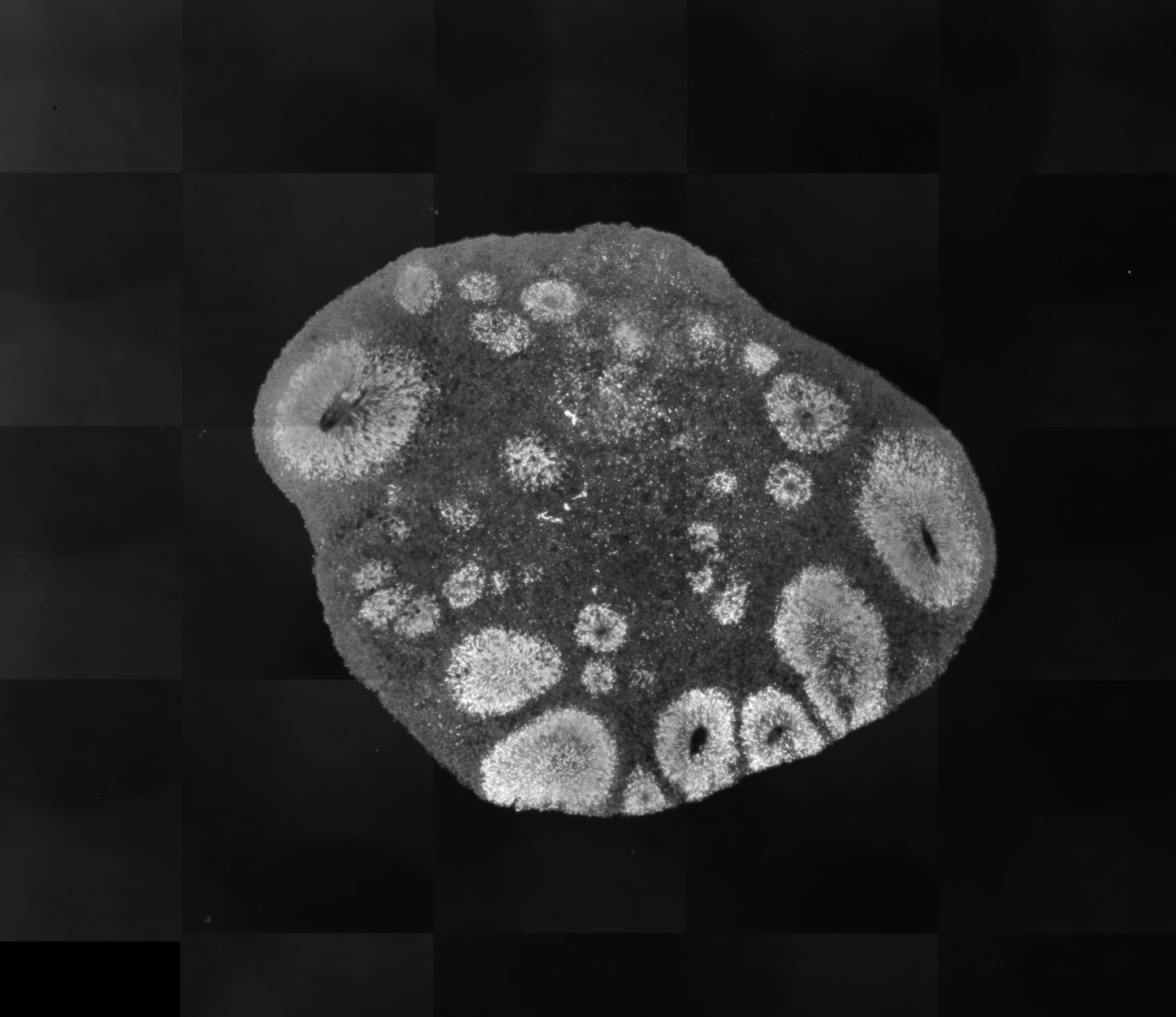

Supplement: Supplementary file 16 — Source data. [file 41556_2024_1412_MOESM16_ESM.zip › Lindenhoferetal-Fig-ED8-sourcedata-NCB/Lindenhoferetal-Fig-ED8-images-NCB/Lindenhoferetal-Fig-ED8-g-RFPWT-GFPKOASPM/Lindenhoferetal-Fig-ED8-g-RFPWT-GFPKOASPM-Sox2.tif]

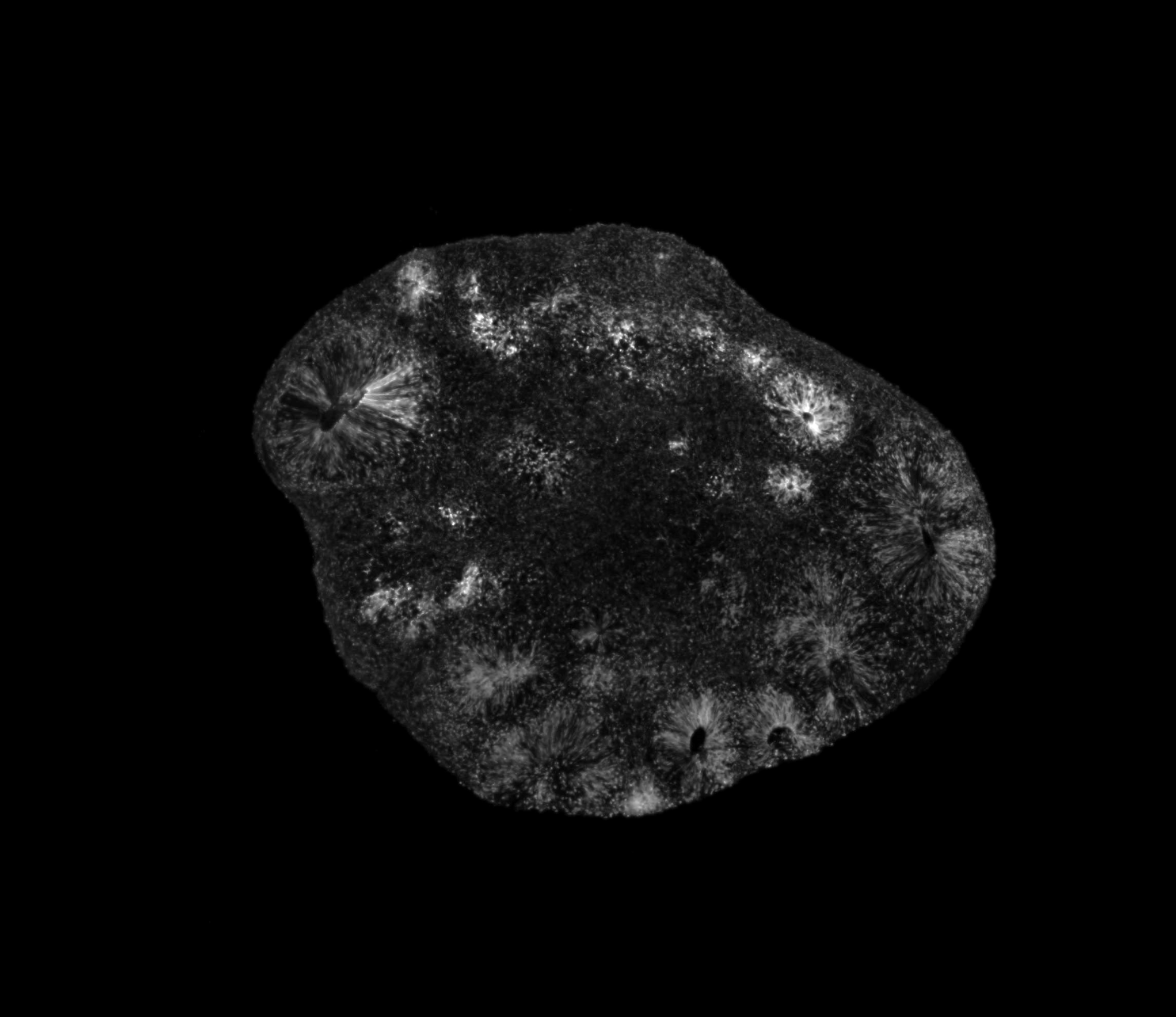

Supplement: Supplementary file 16 — Source data. [file 41556_2024_1412_MOESM16_ESM.zip › Lindenhoferetal-Fig-ED8-sourcedata-NCB/Lindenhoferetal-Fig-ED8-images-NCB/Lindenhoferetal-Fig-ED8-g-RFPWT-GFPKOASPM/Lindenhoferetal-Fig-ED8-g-RFPWT-GFPKOASPM-GFP.tif]

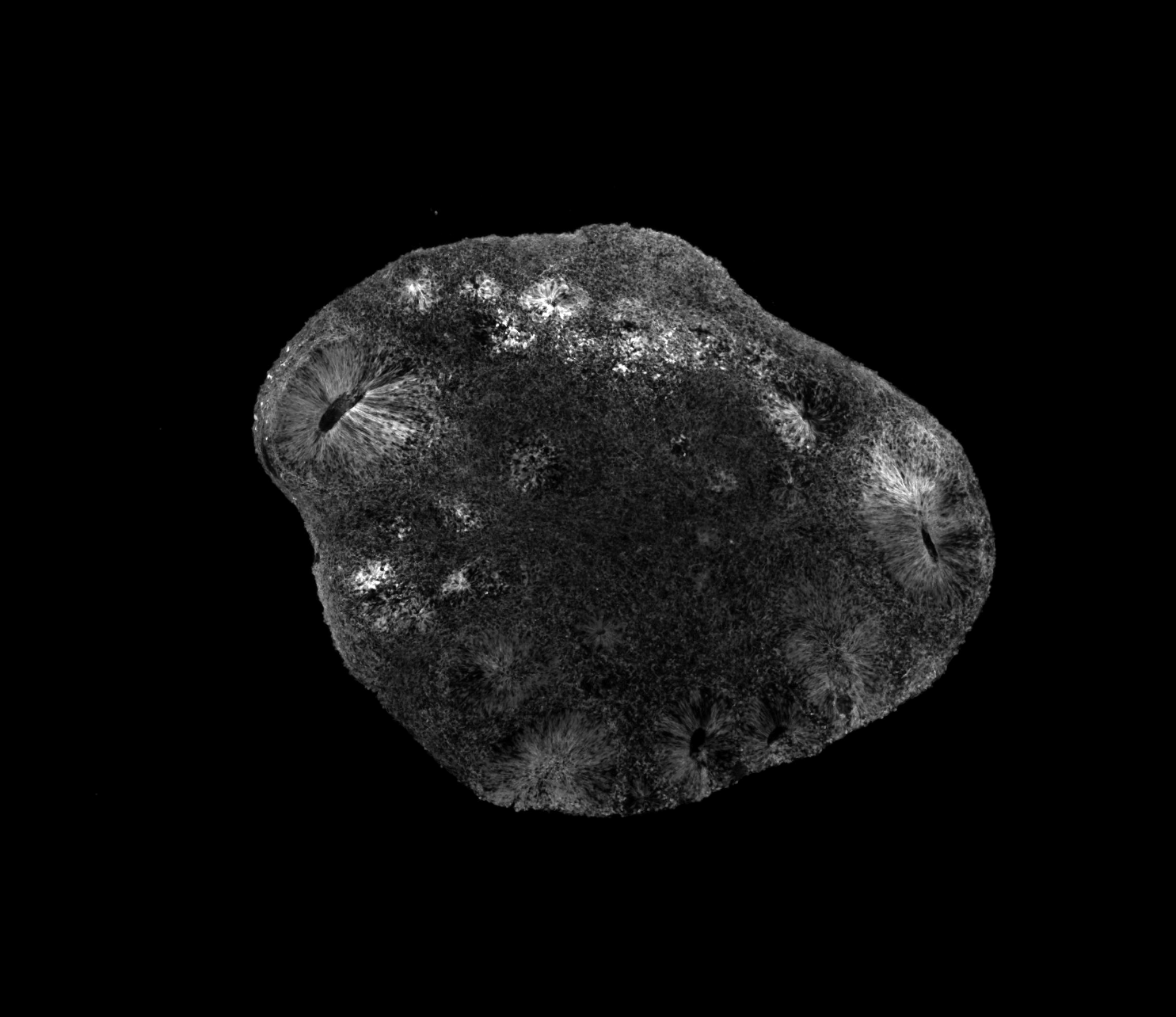

Supplement: Supplementary file 16 — Source data. [file 41556_2024_1412_MOESM16_ESM.zip › Lindenhoferetal-Fig-ED8-sourcedata-NCB/Lindenhoferetal-Fig-ED8-images-NCB/Lindenhoferetal-Fig-ED8-g-RFPWT-GFPKOASPM/Lindenhoferetal-Fig-ED8-g-RFPWT-GFPKOASPM-RFP.tif]

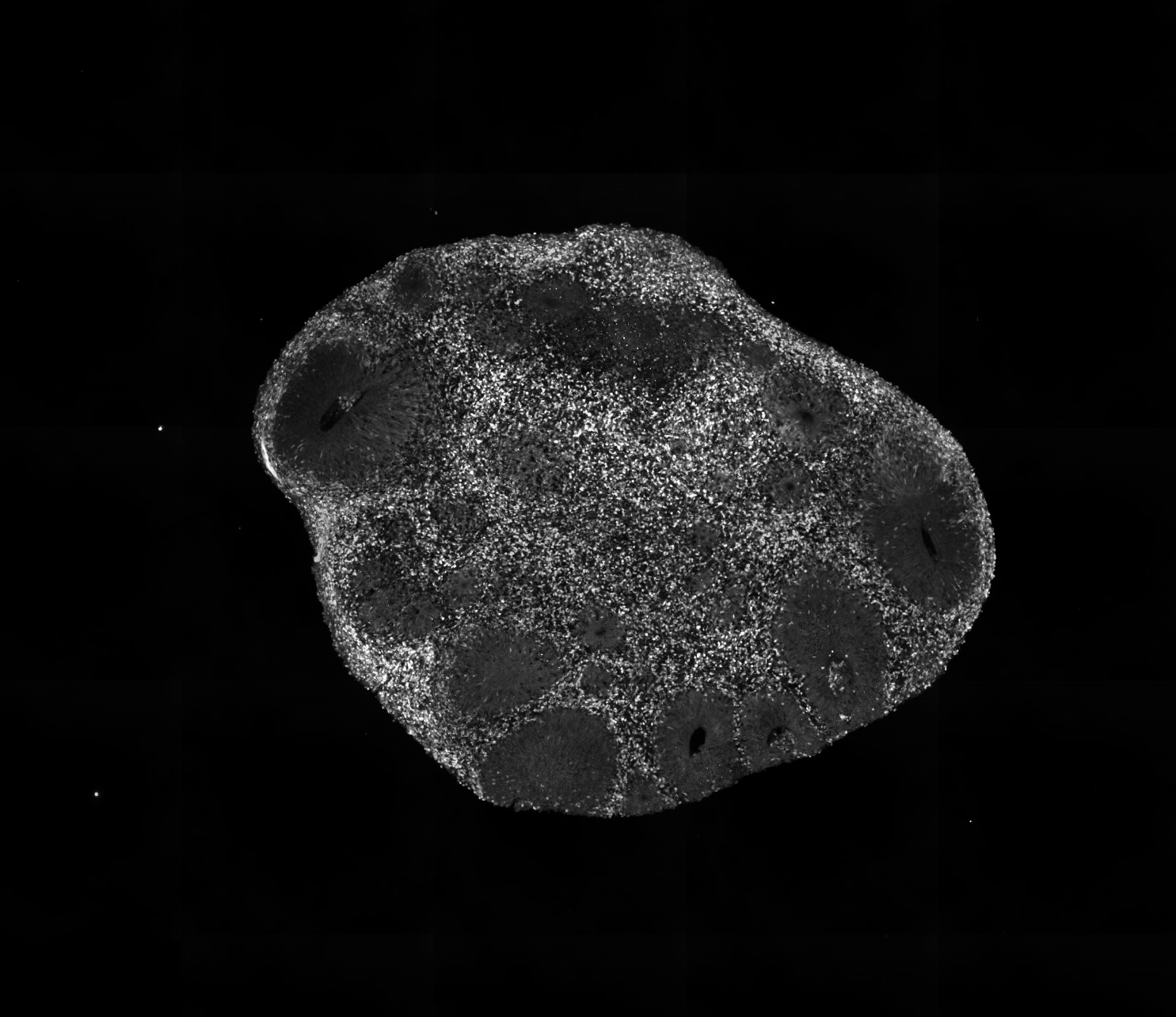

Supplement: Supplementary file 16 — Source data. [file 41556_2024_1412_MOESM16_ESM.zip › Lindenhoferetal-Fig-ED8-sourcedata-NCB/Lindenhoferetal-Fig-ED8-images-NCB/Lindenhoferetal-Fig-ED8-g-RFPWT-GFPKOASPM/Lindenhoferetal-Fig-ED8-g-RFPWT-GFPKOASPM-NeuN.tif]

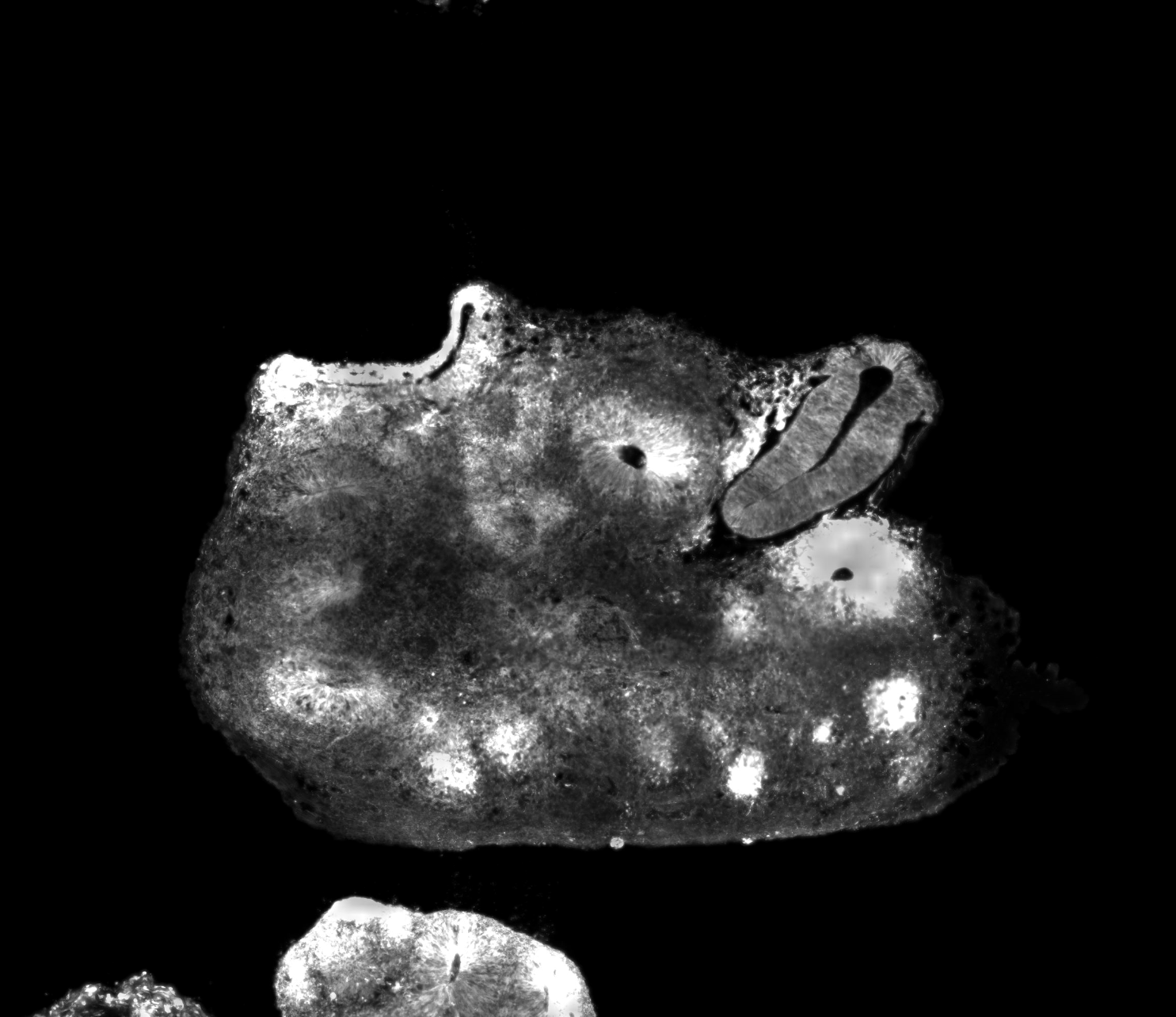

Supplement: Supplementary file 16 — Source data. [file 41556_2024_1412_MOESM16_ESM.zip › Lindenhoferetal-Fig-ED8-sourcedata-NCB/Lindenhoferetal-Fig-ED8-images-NCB/Lindenhoferetal-Fig-ED8-g-RFPWT-GFPKOTP53/Lindenhoferetal-Fig-ED8-g-RFPWT-GFPKOTP53-GFP.tif]

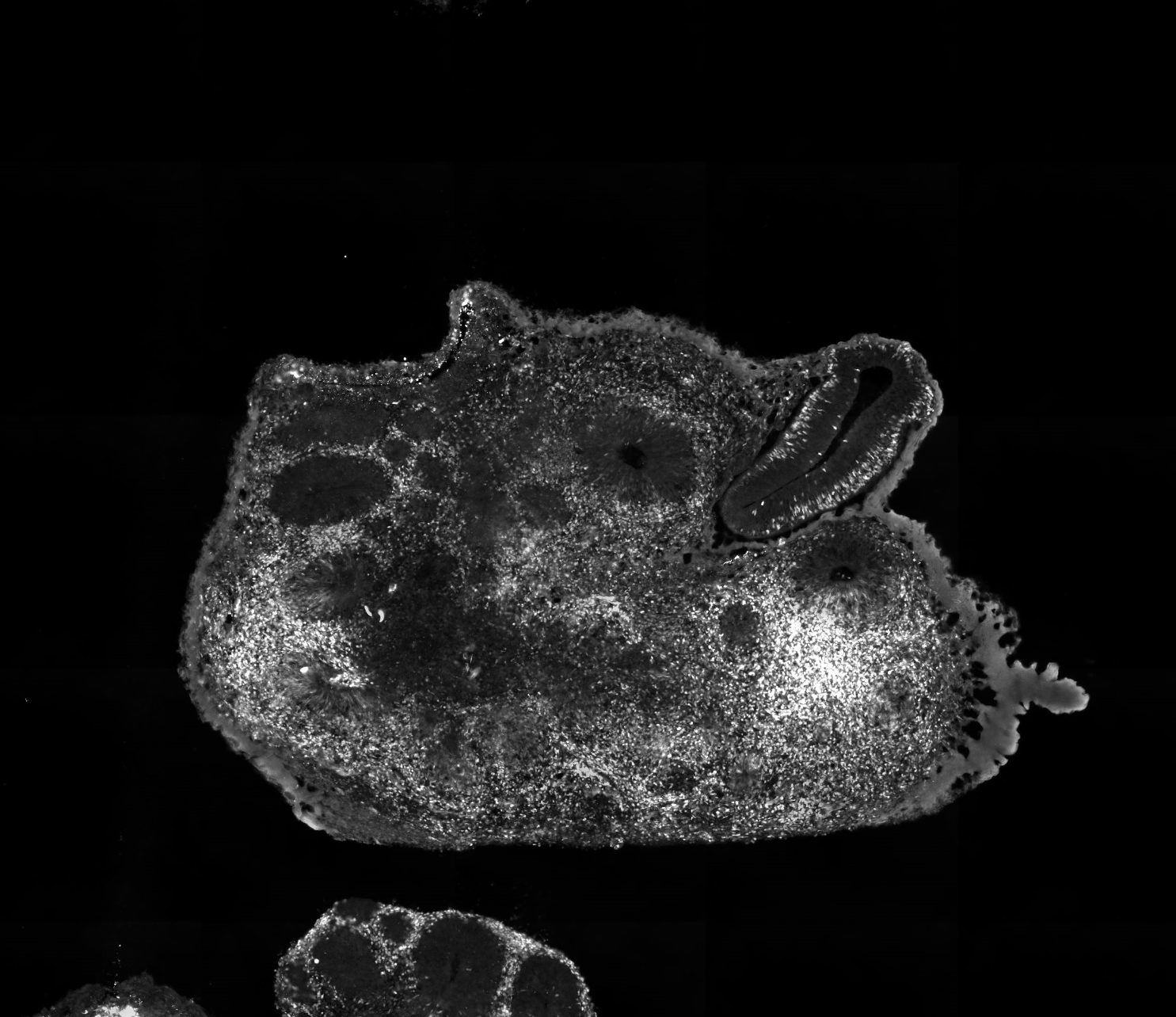

Supplement: Supplementary file 16 — Source data. [file 41556_2024_1412_MOESM16_ESM.zip › Lindenhoferetal-Fig-ED8-sourcedata-NCB/Lindenhoferetal-Fig-ED8-images-NCB/Lindenhoferetal-Fig-ED8-g-RFPWT-GFPKOTP53/Lindenhoferetal-Fig-ED8-g-RFPWT-GFPKOTP53-NeuN.tif]

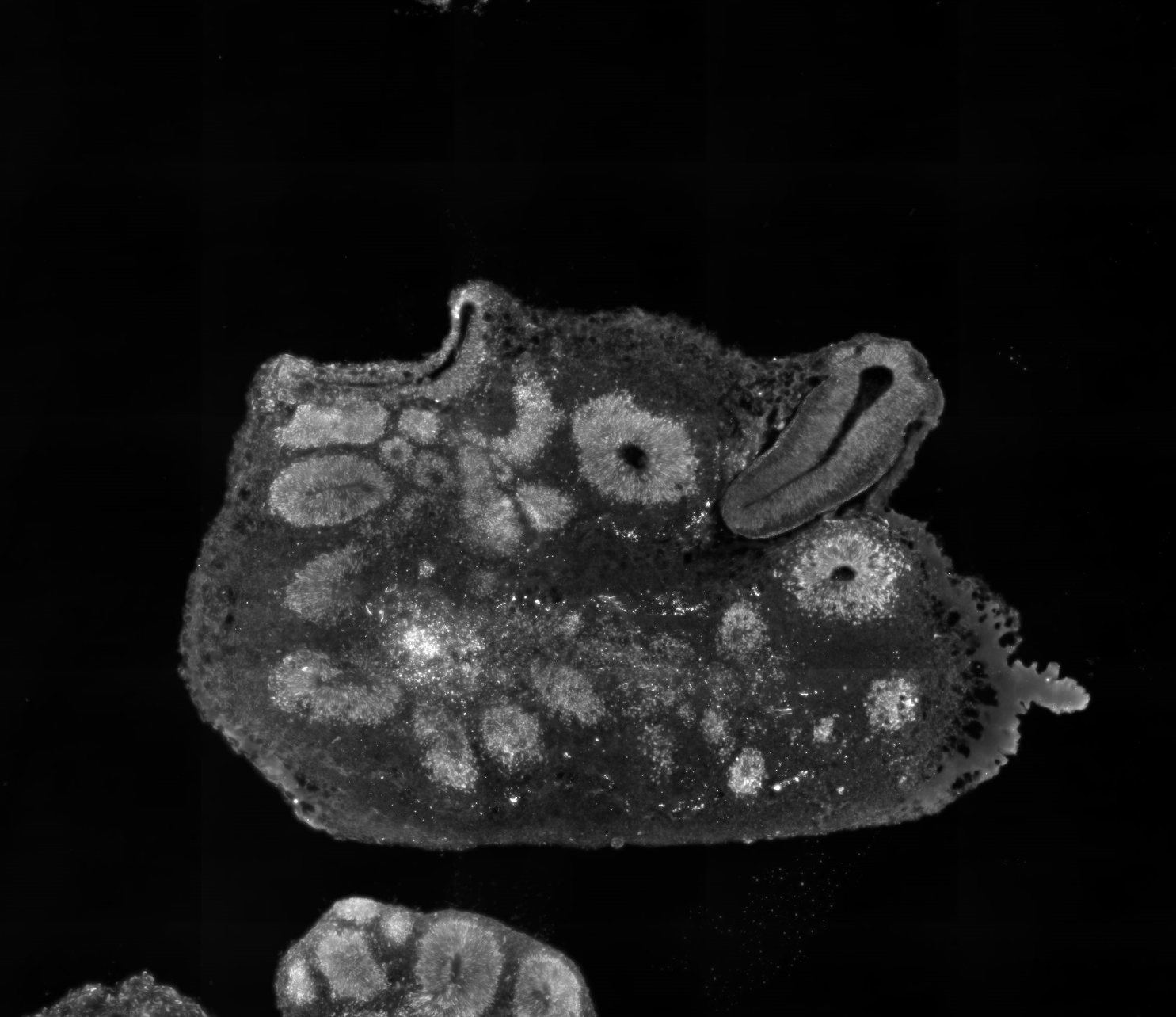

Supplement: Supplementary file 16 — Source data. [file 41556_2024_1412_MOESM16_ESM.zip › Lindenhoferetal-Fig-ED8-sourcedata-NCB/Lindenhoferetal-Fig-ED8-images-NCB/Lindenhoferetal-Fig-ED8-g-RFPWT-GFPKOTP53/Lindenhoferetal-Fig-ED8-g-RFPWT-GFPKOTP53-Sox2.tif]

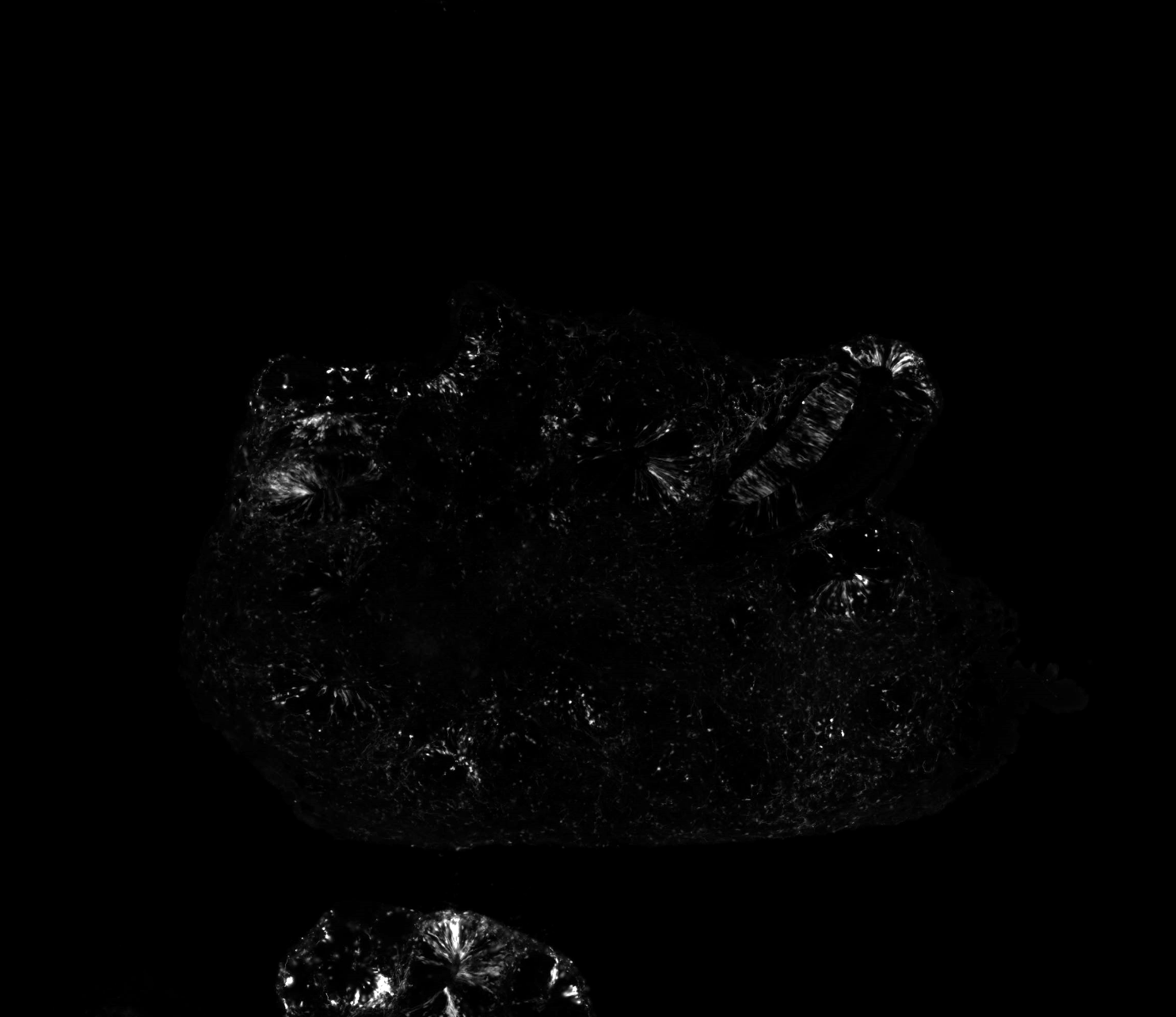

Supplement: Supplementary file 16 — Source data. [file 41556_2024_1412_MOESM16_ESM.zip › Lindenhoferetal-Fig-ED8-sourcedata-NCB/Lindenhoferetal-Fig-ED8-images-NCB/Lindenhoferetal-Fig-ED8-g-RFPWT-GFPKOTP53/Lindenhoferetal-Fig-ED8-g-RFPWT-GFPKOTP53-RFPtif.tif]

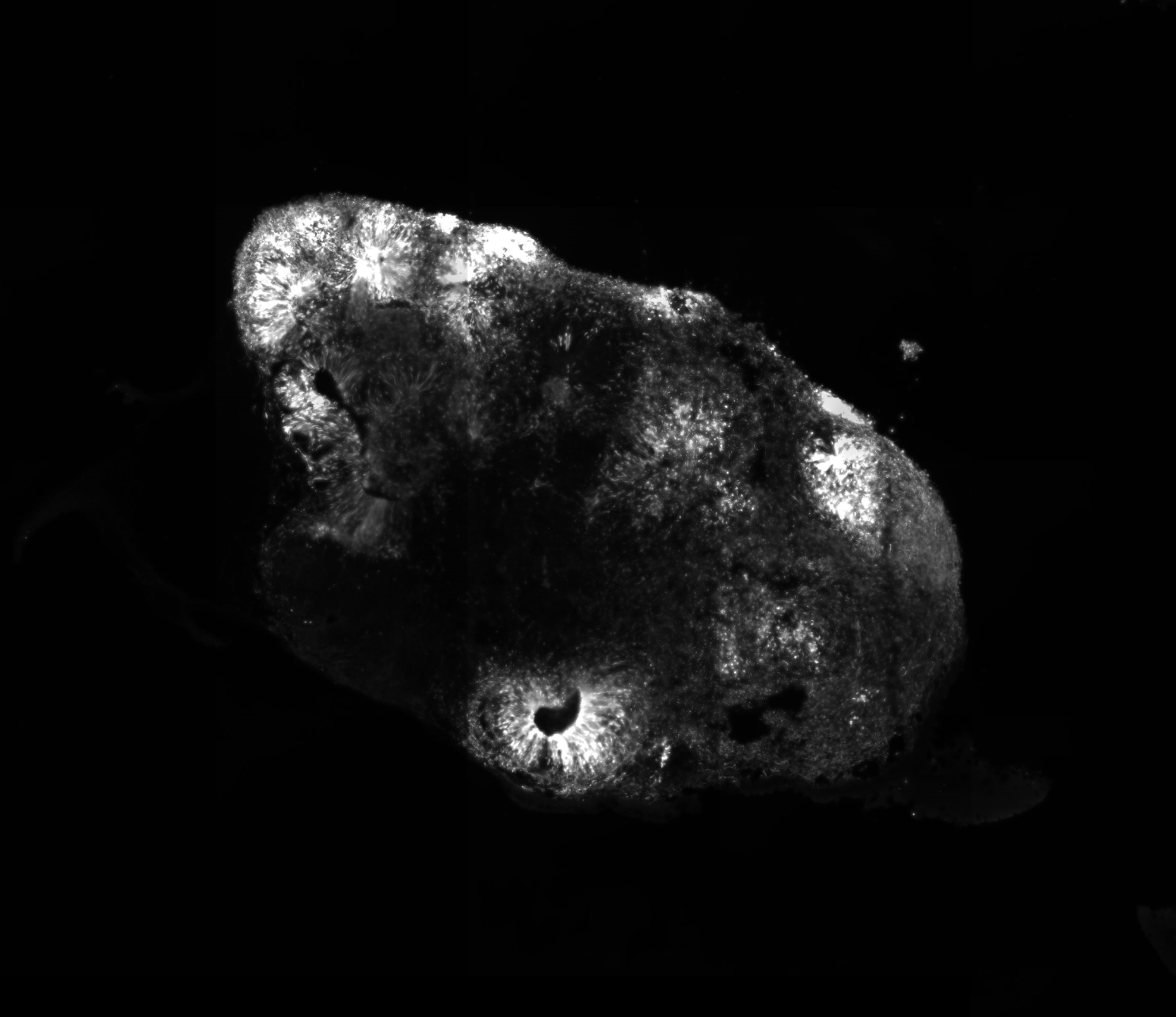

Supplement: Supplementary file 16 — Source data. [file 41556_2024_1412_MOESM16_ESM.zip › Lindenhoferetal-Fig-ED8-sourcedata-NCB/Lindenhoferetal-Fig-ED8-images-NCB/Lindenhoferetal-Fig-ED8-g-RFPWT-GFPWT/Lindenhoferetal-Fig-ED8-g-RFPWT-GFPWT-GFP.tif]

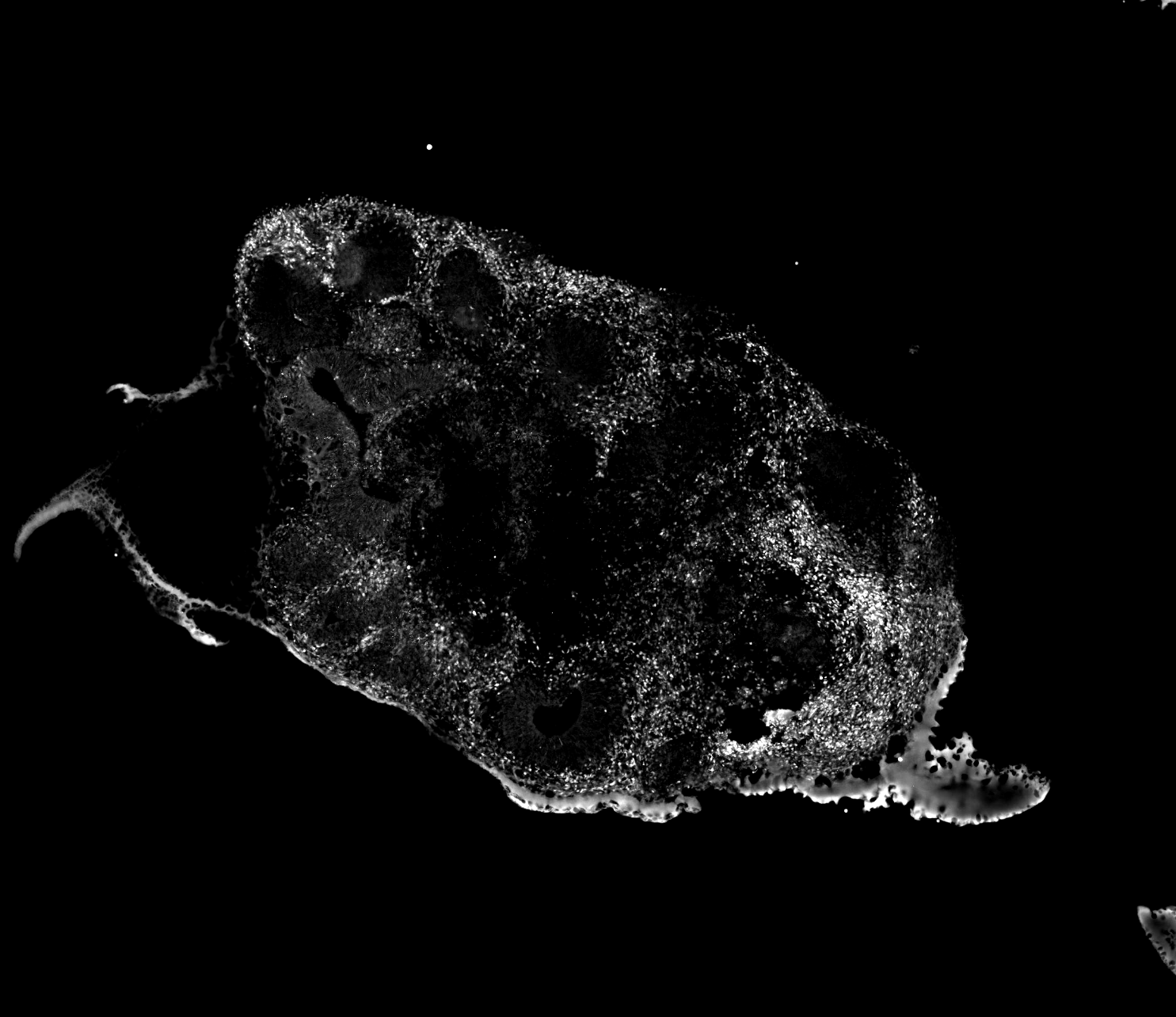

Supplement: Supplementary file 16 — Source data. [file 41556_2024_1412_MOESM16_ESM.zip › Lindenhoferetal-Fig-ED8-sourcedata-NCB/Lindenhoferetal-Fig-ED8-images-NCB/Lindenhoferetal-Fig-ED8-g-RFPWT-GFPWT/Lindenhoferetal-Fig-ED8-g-RFPWT-GFPWT-NeuN.tif]

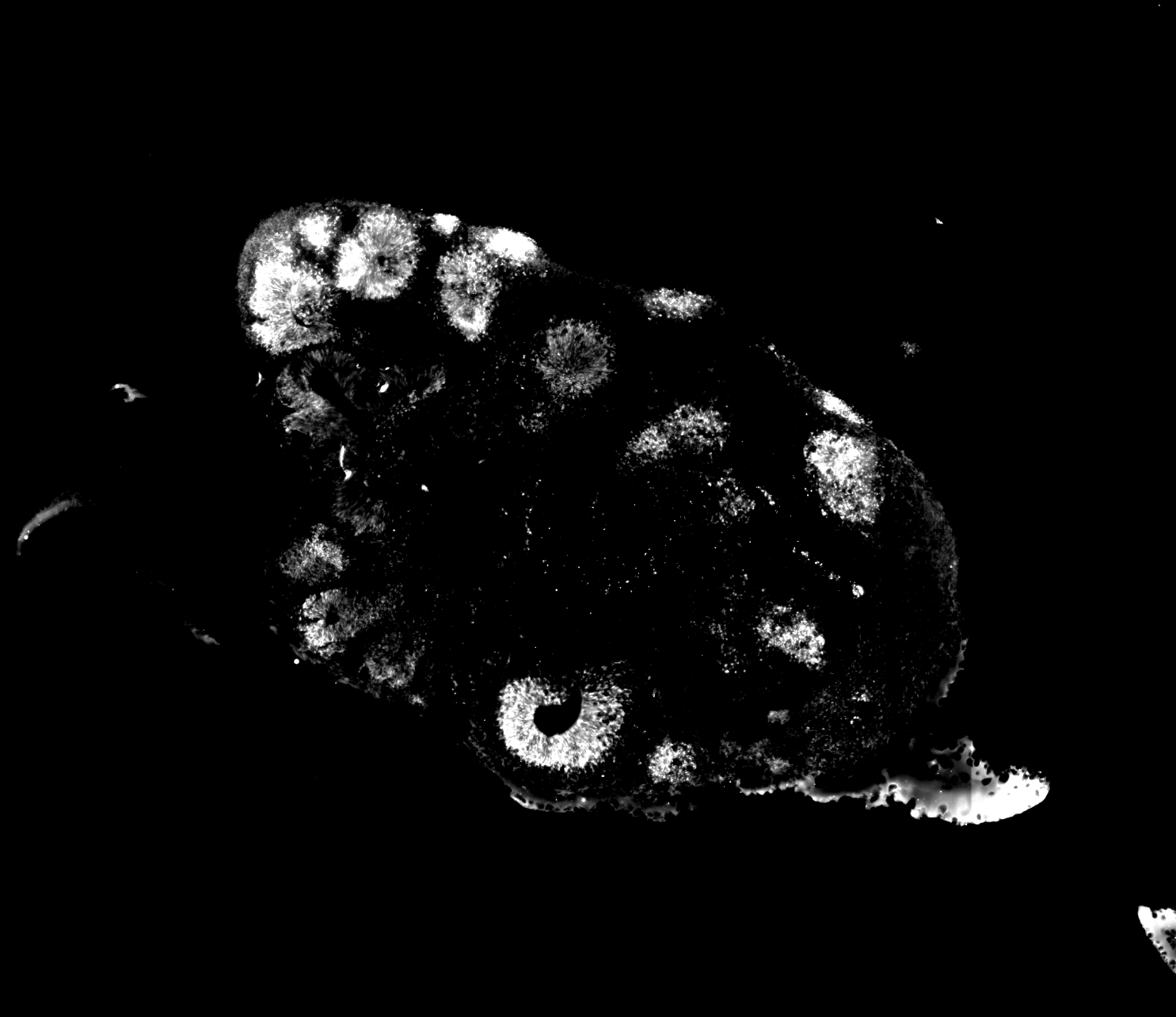

Supplement: Supplementary file 16 — Source data. [file 41556_2024_1412_MOESM16_ESM.zip › Lindenhoferetal-Fig-ED8-sourcedata-NCB/Lindenhoferetal-Fig-ED8-images-NCB/Lindenhoferetal-Fig-ED8-g-RFPWT-GFPWT/Lindenhoferetal-Fig-ED8-g-RFPWT-GFPWT-Sox2.tif]

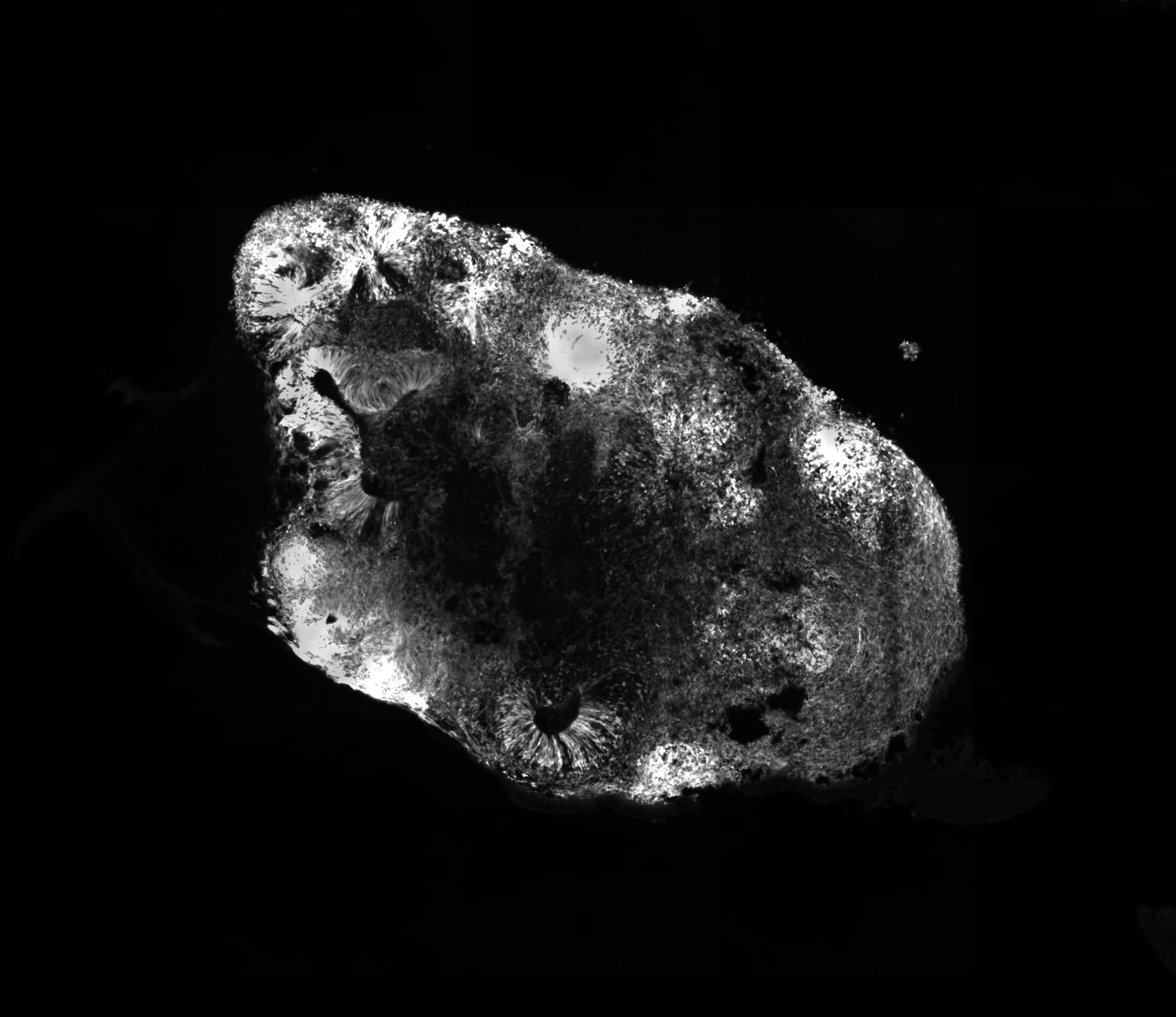

Supplement: Supplementary file 16 — Source data. [file 41556_2024_1412_MOESM16_ESM.zip › Lindenhoferetal-Fig-ED8-sourcedata-NCB/Lindenhoferetal-Fig-ED8-images-NCB/Lindenhoferetal-Fig-ED8-g-RFPWT-GFPWT/Lindenhoferetal-Fig-ED8-g-RFPWT-GFPWT-RFP.tif]

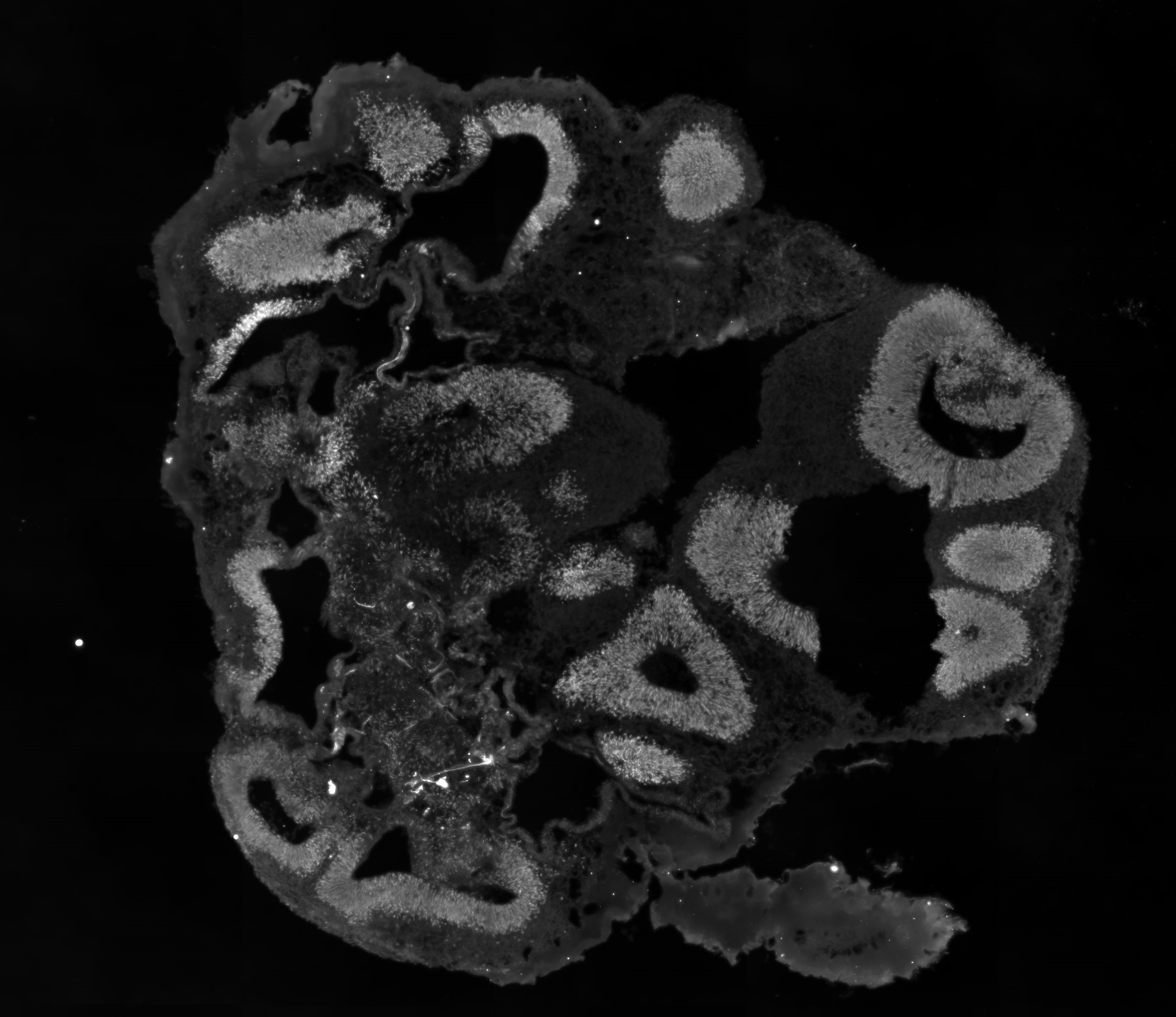

Supplement: Supplementary file 16 — Source data. [file 41556_2024_1412_MOESM16_ESM.zip › Lindenhoferetal-Fig-ED8-sourcedata-NCB/Lindenhoferetal-Fig-ED8-images-NCB/Lindenhoferetal-Fig-ED8-g-RFPWT-GFPKOPAX6/Lindenhoferetal-Fig-ED8-g-RFPWT-GFPKOPAX6-Sox2.tif]

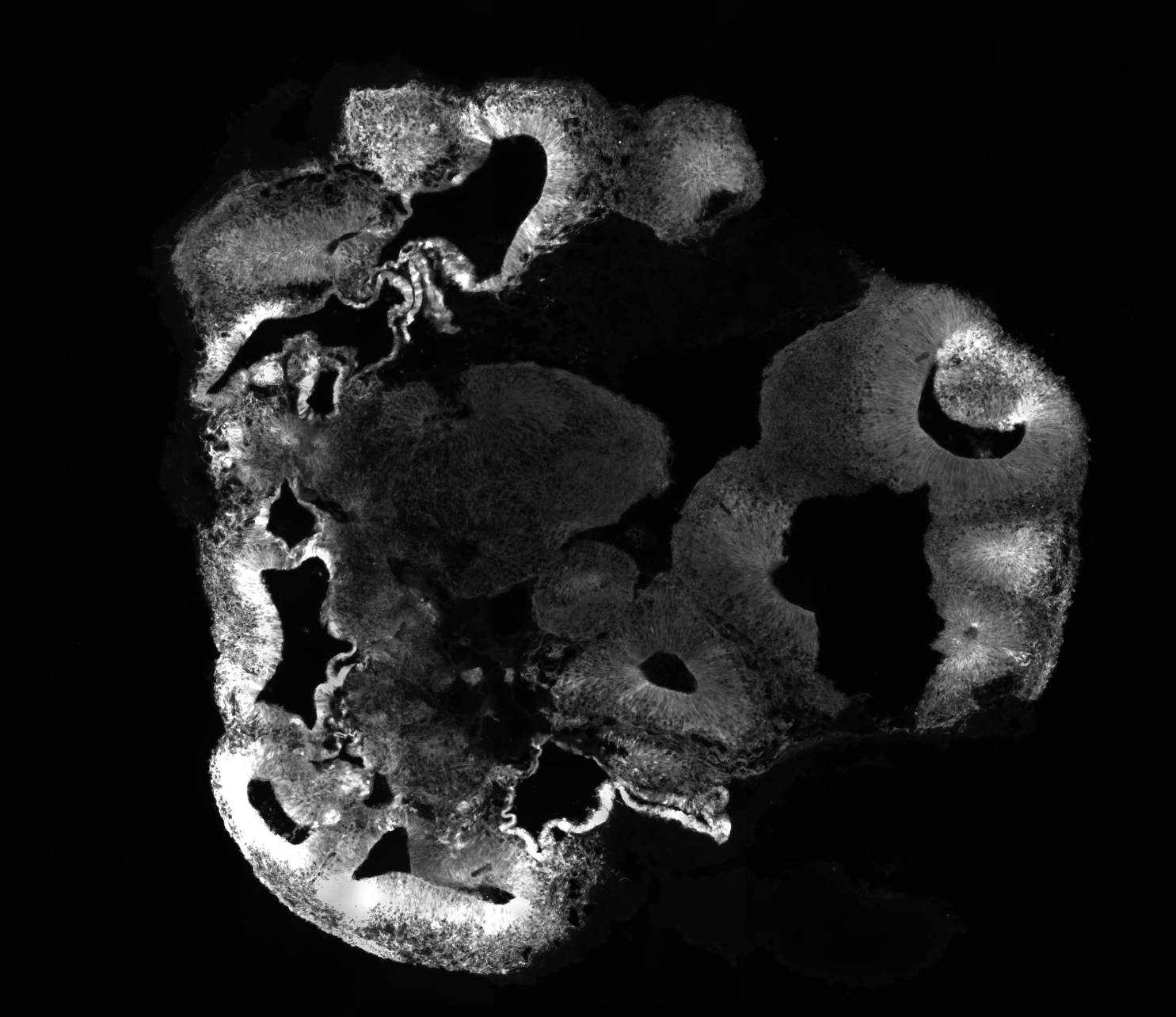

Supplement: Supplementary file 16 — Source data. [file 41556_2024_1412_MOESM16_ESM.zip › Lindenhoferetal-Fig-ED8-sourcedata-NCB/Lindenhoferetal-Fig-ED8-images-NCB/Lindenhoferetal-Fig-ED8-g-RFPWT-GFPKOPAX6/Lindenhoferetal-Fig-ED8-g-RFPWT-GFPKOPAX6-RFP.tif]

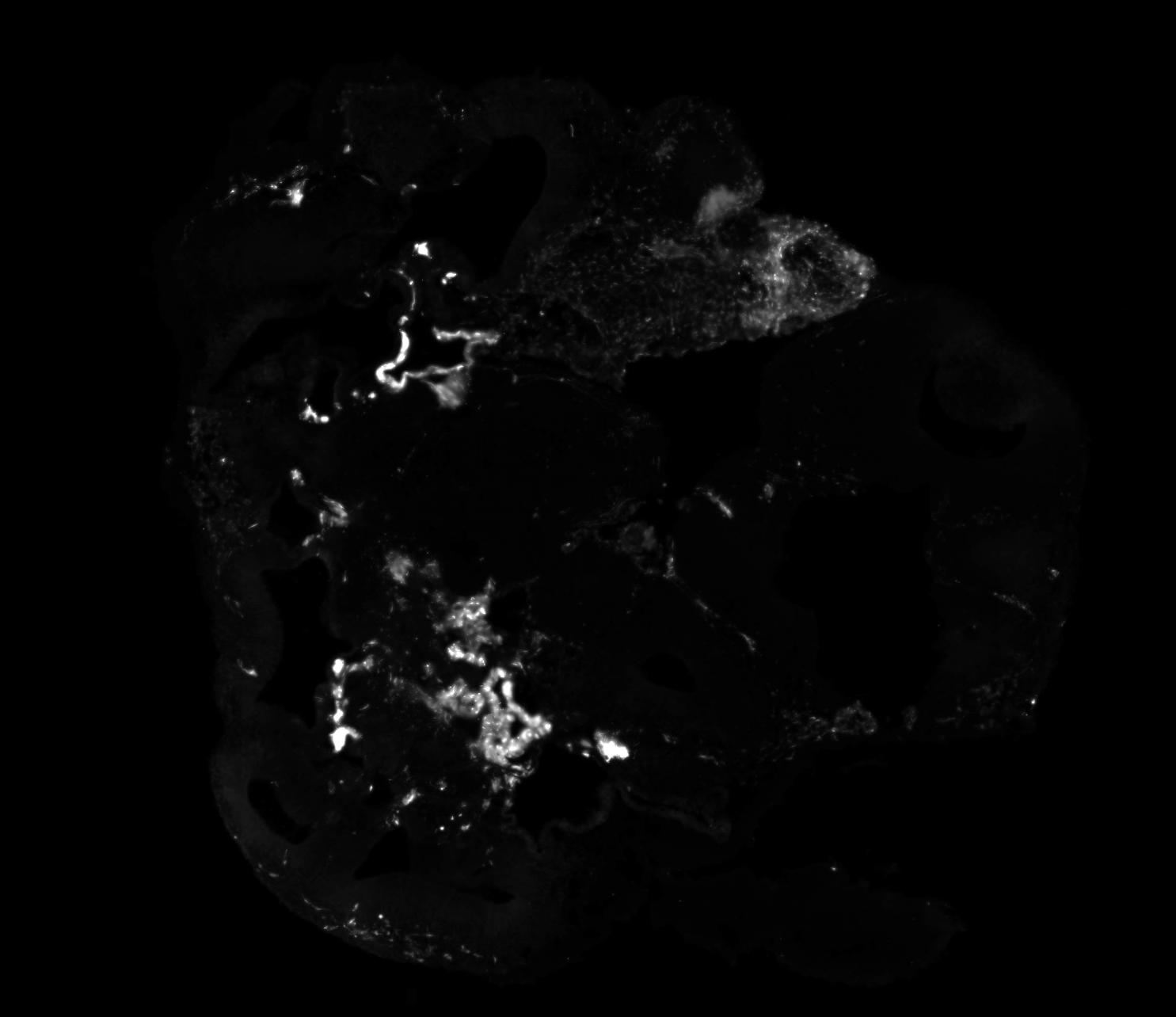

Supplement: Supplementary file 16 — Source data. [file 41556_2024_1412_MOESM16_ESM.zip › Lindenhoferetal-Fig-ED8-sourcedata-NCB/Lindenhoferetal-Fig-ED8-images-NCB/Lindenhoferetal-Fig-ED8-g-RFPWT-GFPKOPAX6/Lindenhoferetal-Fig-ED8-g-RFPWT-GFPKOPAX6-GFP.tif]

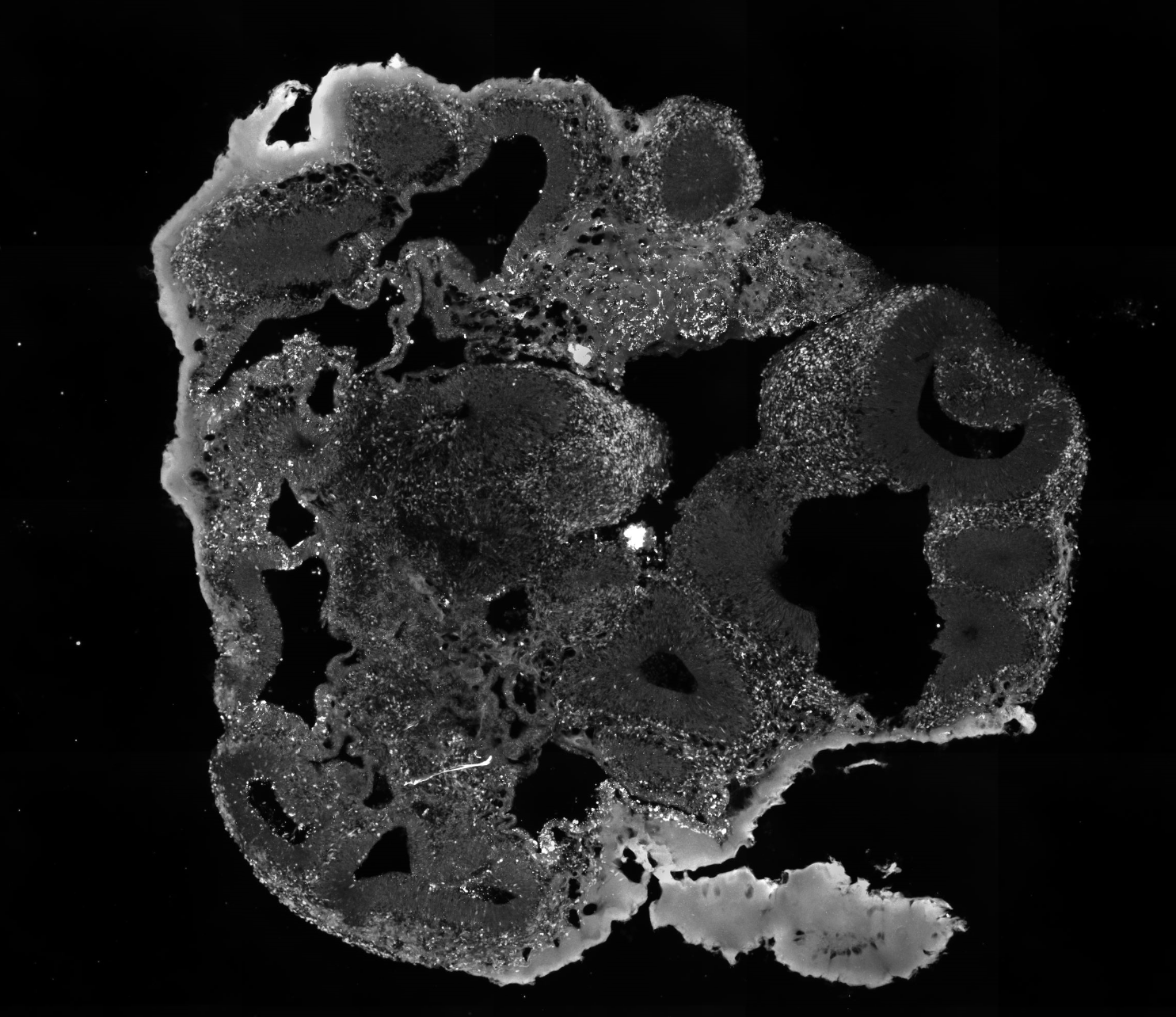

Supplement: Supplementary file 16 — Source data. [file 41556_2024_1412_MOESM16_ESM.zip › Lindenhoferetal-Fig-ED8-sourcedata-NCB/Lindenhoferetal-Fig-ED8-images-NCB/Lindenhoferetal-Fig-ED8-g-RFPWT-GFPKOPAX6/Lindenhoferetal-Fig-ED8-g-RFPWT-GFPKOPAX6-NeuN.tif]

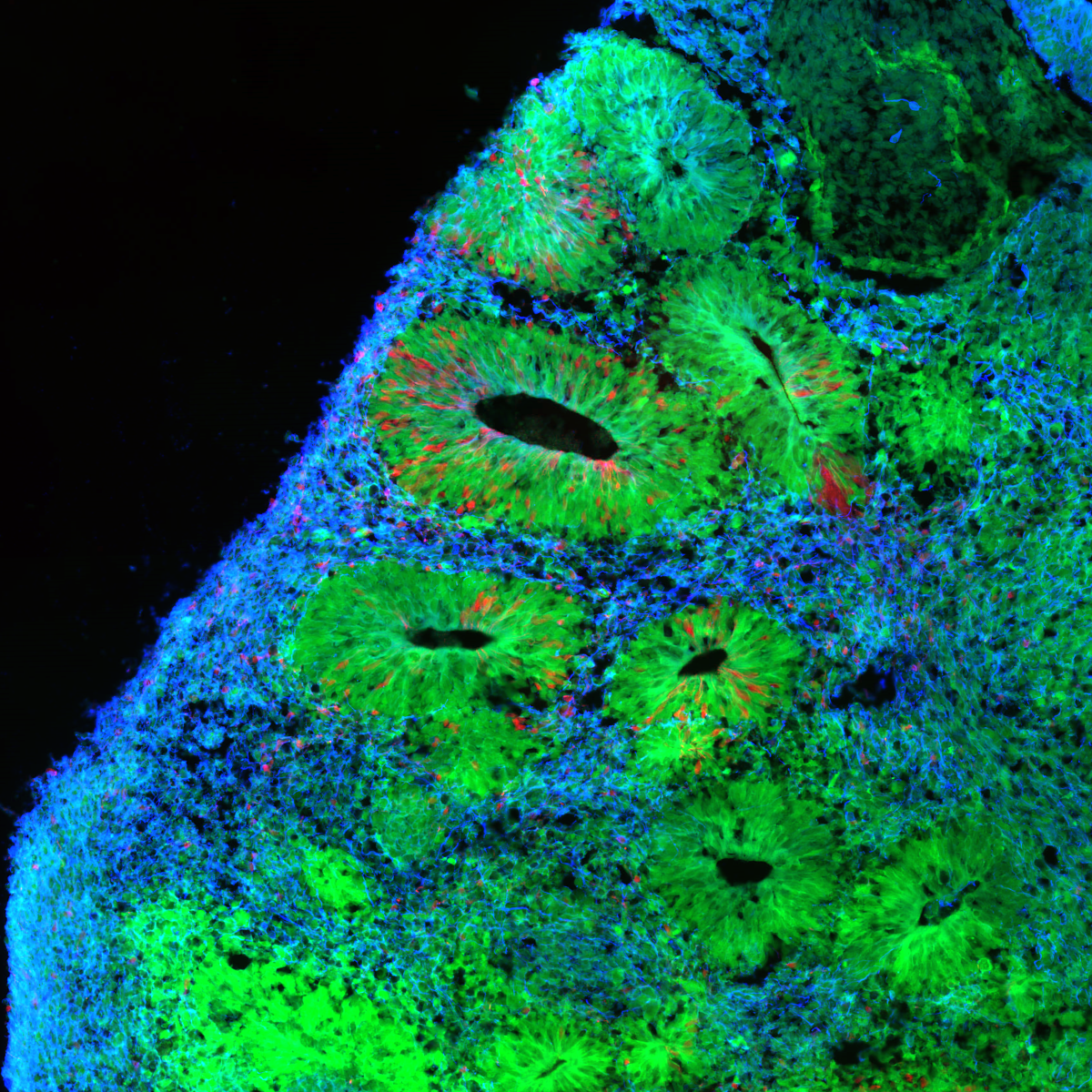

Supplement: Supplementary file 17 — Source data. [file 41556_2024_1412_MOESM17_ESM.zip › Lindenhoferetal-Fig-ED9-sourcedata-NCB/Lindenhoferetal-Fig-ED9-images-NCB/Lindenhoferetal-Fig-ED9-b.tif]

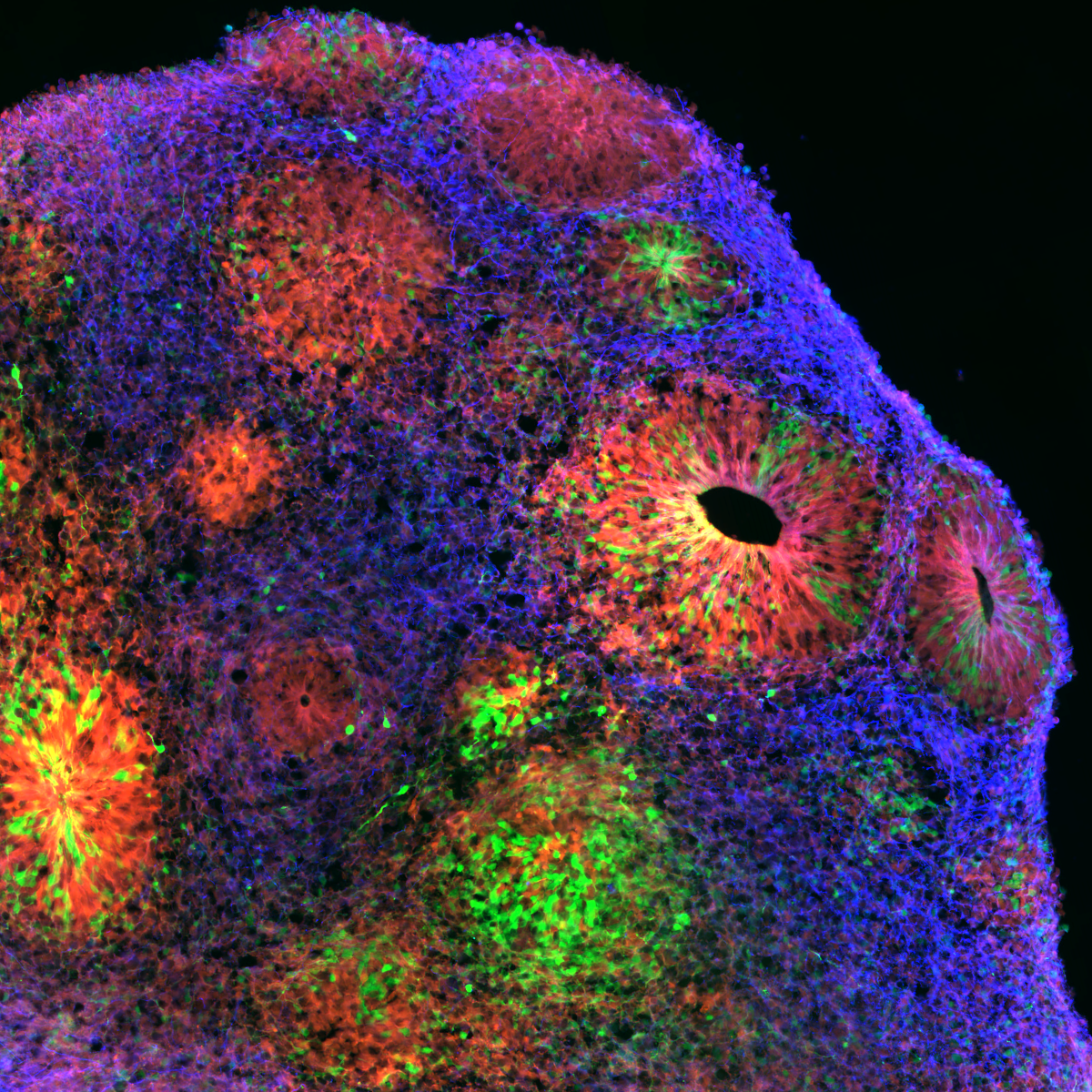

Supplement: Supplementary file 17 — Source data. [file 41556_2024_1412_MOESM17_ESM.zip › Lindenhoferetal-Fig-ED9-sourcedata-NCB/Lindenhoferetal-Fig-ED9-images-NCB/Lindenhoferetal-Fig-ED9-c.tif]

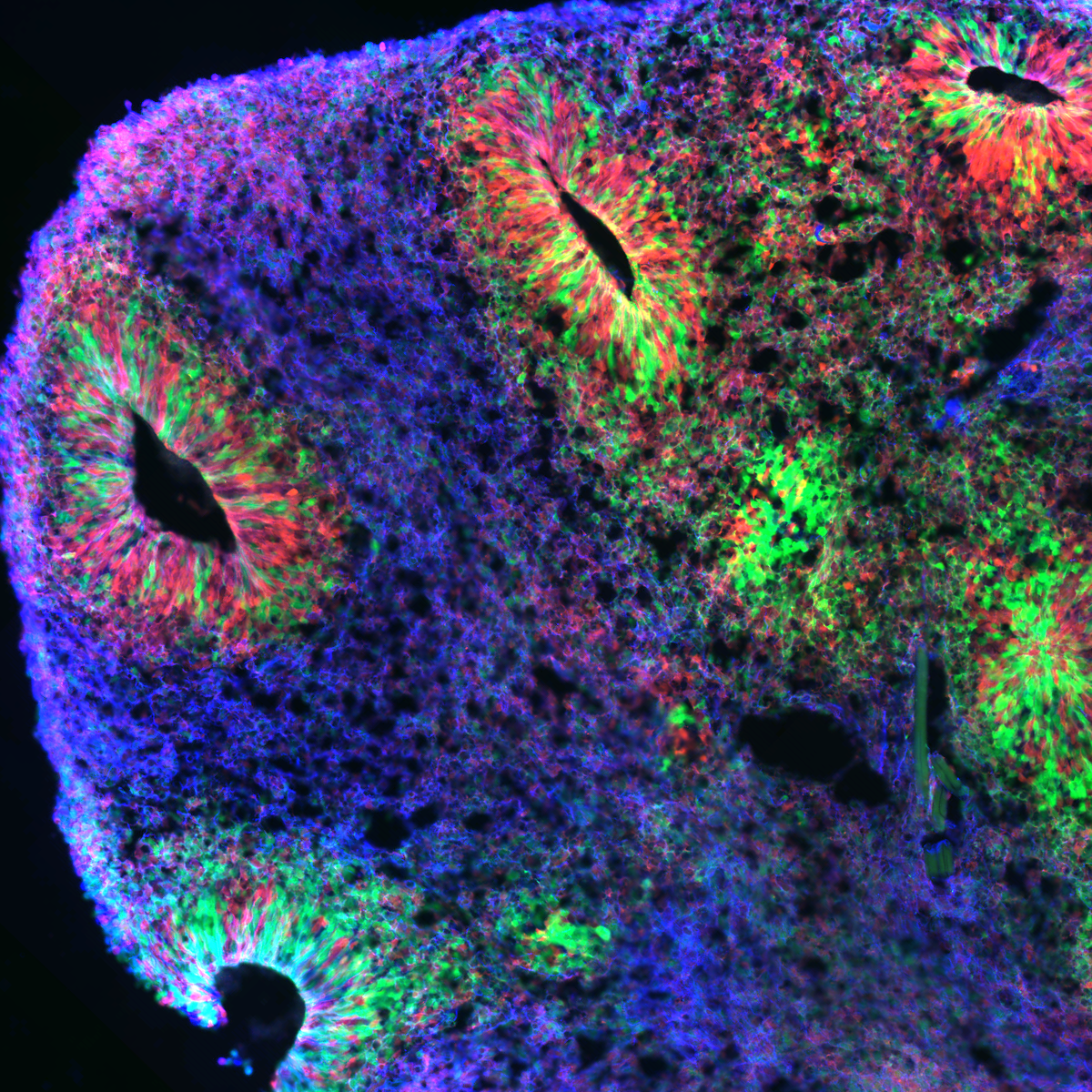

Supplement: Supplementary file 17 — Source data. [file 41556_2024_1412_MOESM17_ESM.zip › Lindenhoferetal-Fig-ED9-sourcedata-NCB/Lindenhoferetal-Fig-ED9-images-NCB/Lindenhoferetal-Fig-ED9-a.tif]

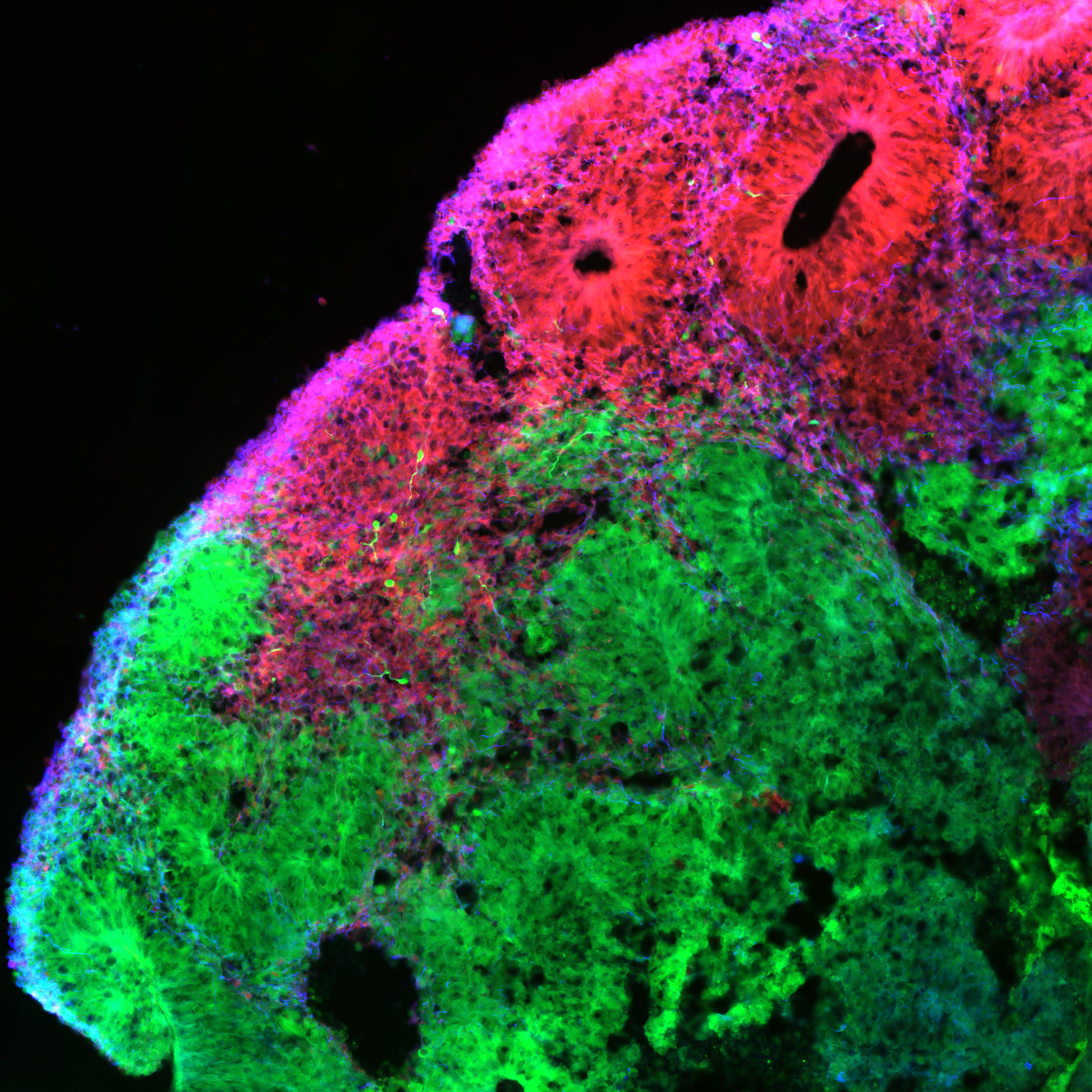

Supplement: Supplementary file 17 — Source data. [file 41556_2024_1412_MOESM17_ESM.zip › Lindenhoferetal-Fig-ED9-sourcedata-NCB/Lindenhoferetal-Fig-ED9-images-NCB/Lindenhoferetal-Fig-ED9-d.tif]

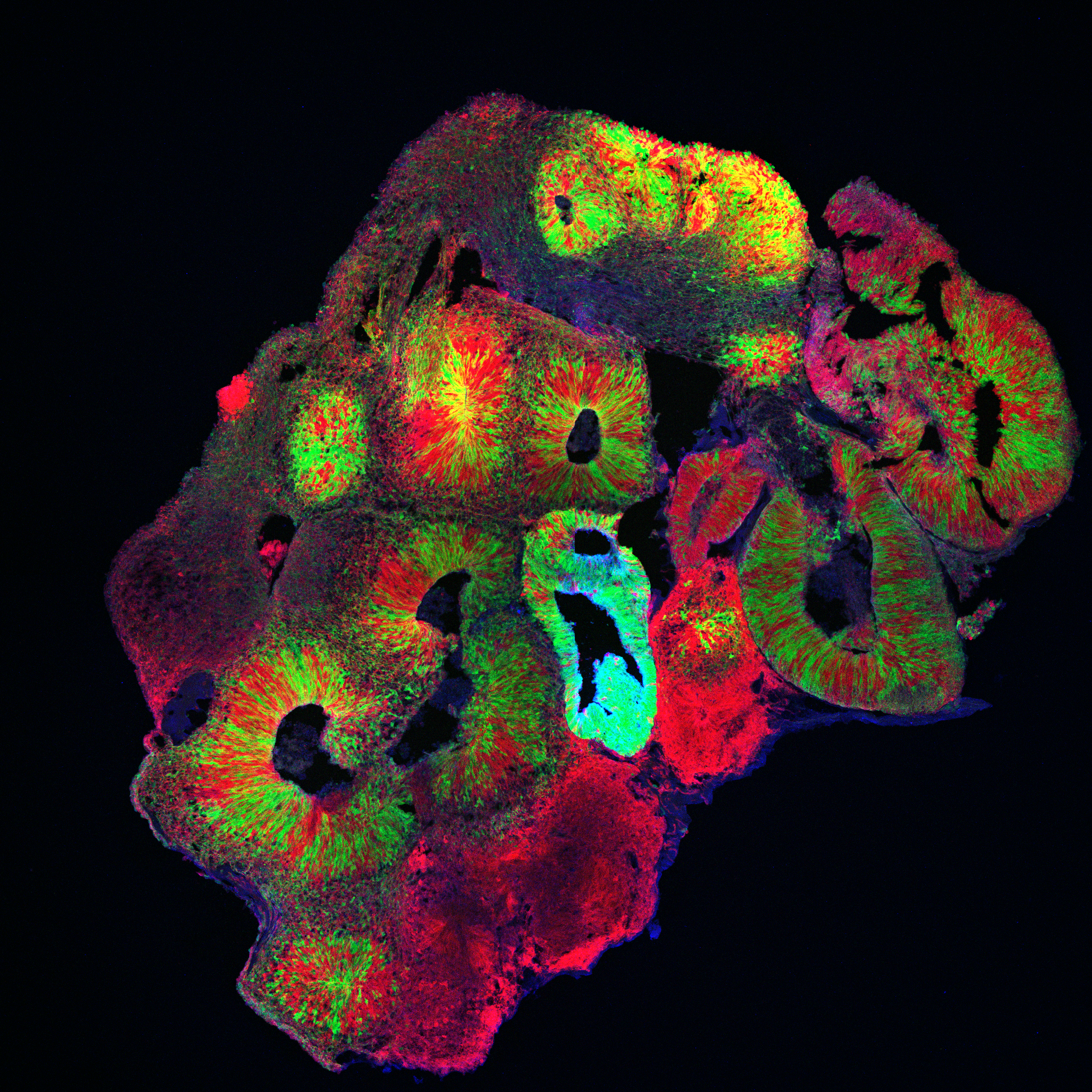

Supplement: Supplementary file 18 — Source data. [file 41556_2024_1412_MOESM18_ESM.zip › Lindenhoferetal-Fig-ED10-sourcedata-NCB/Lindenhoferetal-Fig-ED10-images-NCB/Lindenhoferetal-Fig-ED10-a-1.tif]

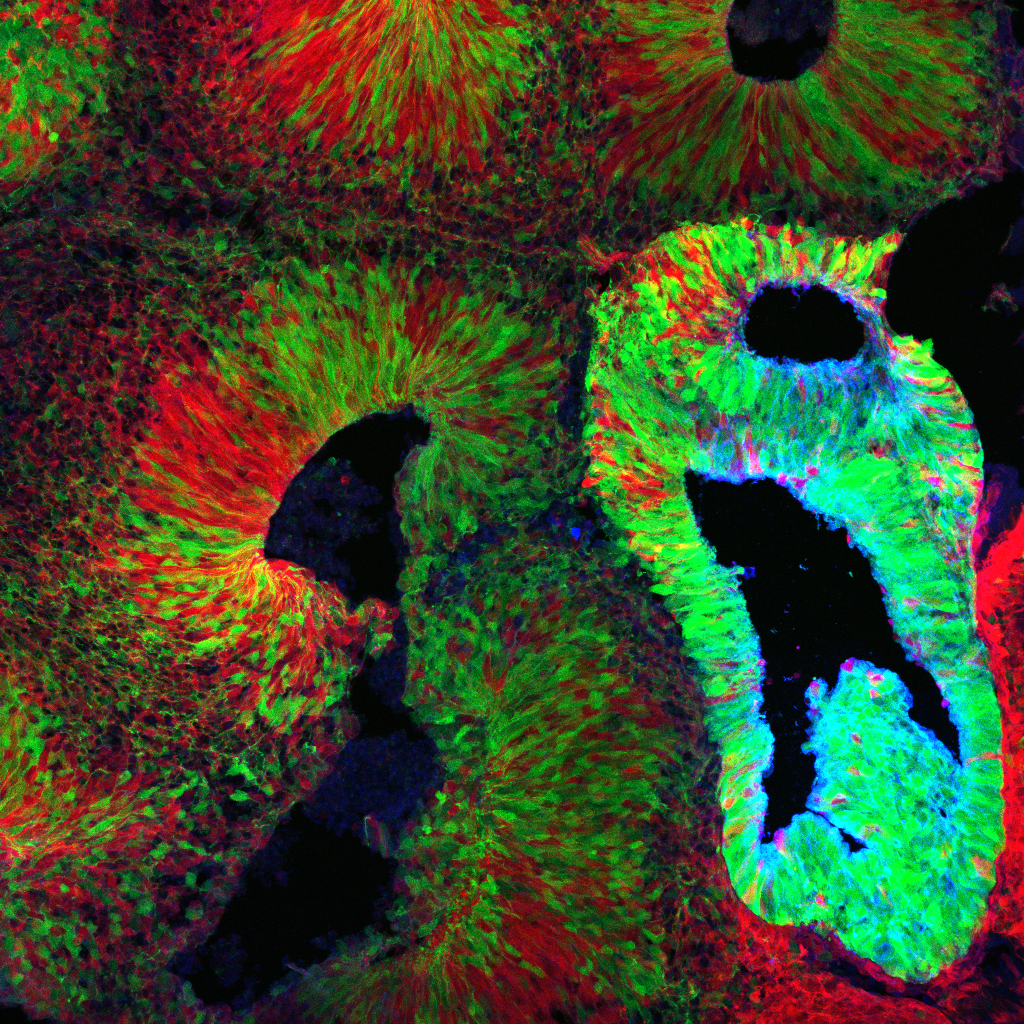

Supplement: Supplementary file 18 — Source data. [file 41556_2024_1412_MOESM18_ESM.zip › Lindenhoferetal-Fig-ED10-sourcedata-NCB/Lindenhoferetal-Fig-ED10-images-NCB/Lindenhoferetal-Fig-ED10-a-2.tif]
