# Supplementary figures and images for: Cerebral organoids display dynamic clonal growth and tunable tissue replenishment (part 2 of 2)
Source: Nat Cell Biol. 2024 May 7;26(5):710–8. doi: 10.1038/s41556-024-01412-z (PMC11098754; doi:10.1038/s41556-024-01412-z)

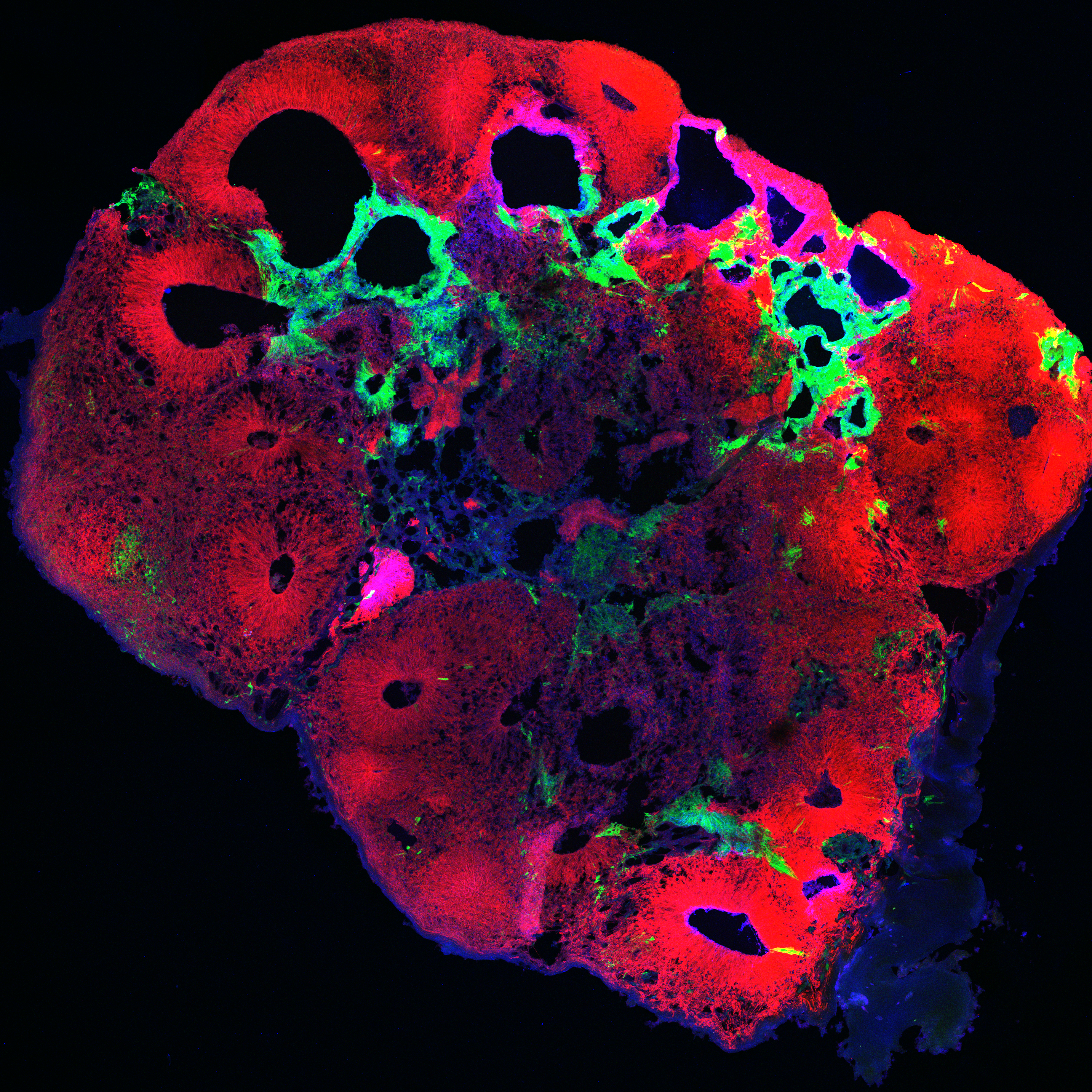

Supplement: Supplementary file 18 — Source data. [file 41556_2024_1412_MOESM18_ESM.zip › Lindenhoferetal-Fig-ED10-sourcedata-NCB/Lindenhoferetal-Fig-ED10-images-NCB/Lindenhoferetal-Fig-ED10-b-1.tif]

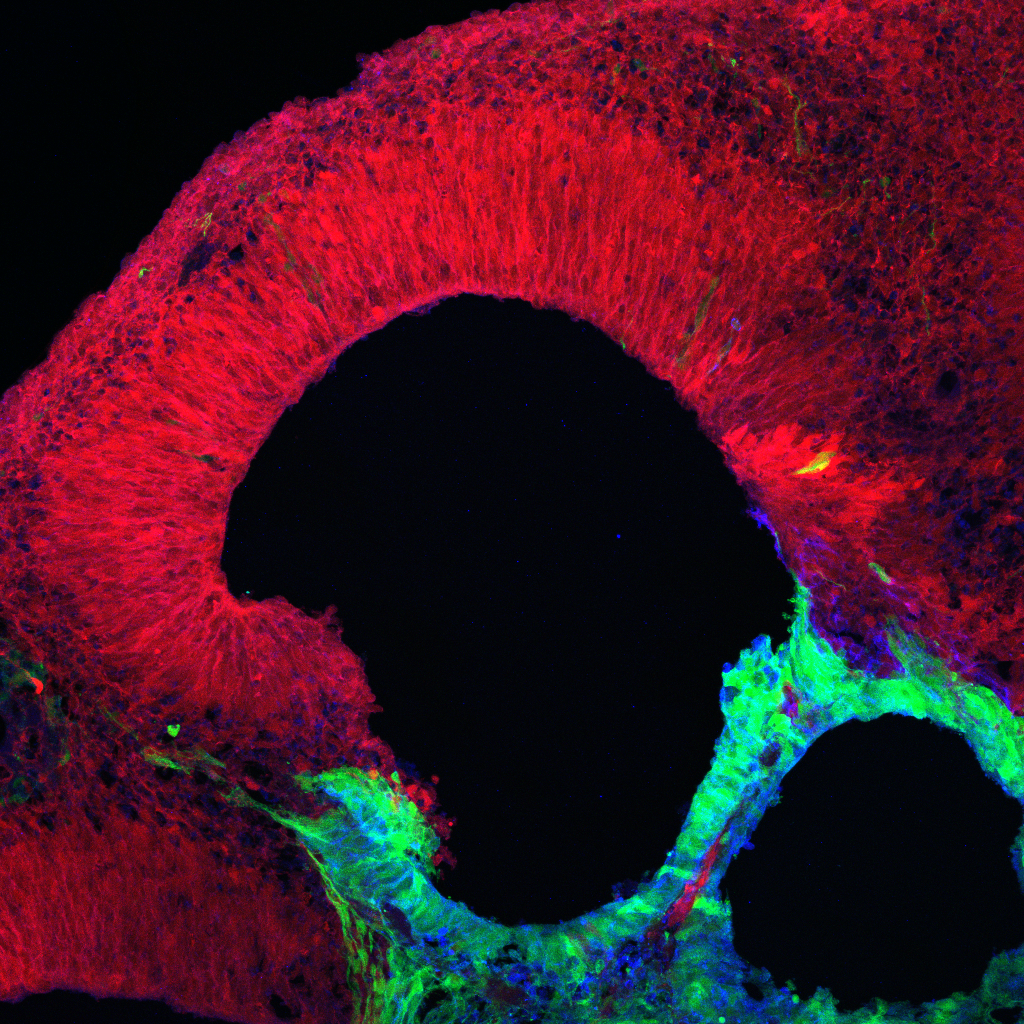

Supplement: Supplementary file 18 — Source data. [file 41556_2024_1412_MOESM18_ESM.zip › Lindenhoferetal-Fig-ED10-sourcedata-NCB/Lindenhoferetal-Fig-ED10-images-NCB/Lindenhoferetal-Fig-ED10-b-2.tif]
